# A Phase‐Separated SR Protein Reprograms Host Pre‐mRNA Splicing to Enhance Disease Susceptibility

**DOI:** 10.1002/advs.202500072

**Published:** 2025-05-08

**Authors:** Dong Yan, Jie Huang, Fengqi Tian, Haidong Shu, Han Chen, Qian Peng, Hongwei Wu, Jianlong Zhao, Anireddy S. N. Reddy, Gang Li, Yuanchao Wang, Suomeng Dong

**Affiliations:** ^1^ State Key Laboratory of Agricultural and Forestry Biosecurity College of Plant Protection Nanjing Agricultural University Nanjing 210095 China; ^2^ The Plant Chemetics Laboratory Department of Biology University of Oxford Oxford OX1 3RB UK; ^3^ State Key Laboratory of Vegetable Biobreeding Institute of Vegetables and Flowers Chinese Academy of Agricultural Sciences Beijing 100081 China; ^4^ Department of Biology and Program in Cell and Molecular Biology Colorado State University Fort Collins CO 80523 USA

**Keywords:** alternative splicing, late blight disease, phase separation, plant immunity, SR proteins

## Abstract

Alternative splicing (AS) plays a vital role in the plant–microbe interaction. Modulating host precursor‐mRNA AS is a key strategy employed by multiple pathogens to subvert plant immunity. However, the underlying mechanism by which the host splicing factor regulates plant immunity remains poorly understood. Here, a plant‐conserved serine/arginine‐rich (SR) RNA splicing factor, SR30, which negatively regulates tomato immunity against the infamous *Phytophthora infestans* (*P. infestans*) is identified. SR30 governs tomato mRNA AS at a genome‐wide level and suppresses defense‐related genes AS. During *P. infestans* infection, SR30 is induced to form nuclear condensates via liquid–liquid phase separation driven by intrinsically disordered regions. Importantly, the phase separation property is required for the function of SR30 in disease susceptibility and the regulation of genes AS. Knockout of *SR30* via CRISPR/Cas9 improves tomato disease resistance to *P. infestans*, *P. capsici*, and *P. parasitica* by promoting defense genes AS. These findings uncover a novel mechanism in a phase‐separated protein that regulates plant immunity by altering the AS of defense‐related genes and provides a new paradigm for engineering protein condensate in crop‐resistant breeding.

## Graphical abstract

This study identifies SR30, a splicing factor, as a negative regulator of tomato immunity. During *Phytophthora infestans* infection, the elevated SR30 forms nuclear condensates to suppress the alternative splicing (AS) of defense‐related genes in a phase separation manner. The knockout of *SR30* enhances disease resistance to different *Phytophthora* pathogens by promoting AS of defense genes, offering insights for crop‐resistant breeding.

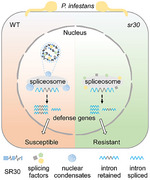

## 1 Introduction

In most eukaryotic organisms, RNA splicing represents a fundamental aspect of gene expression regulation. Precursor‐mRNA (pre‐mRNA) splicing is a process whereby introns are excised while exons are joined together. This process is catalyzed by the spliceosome, which is composed of five small nuclear ribonucleoproteins (snRNPs, U1, U2, U4, U5, and U6) and hundreds of auxiliary splicing regulatory proteins, including the serine/arginine‐rich (SR) family proteins.^[^ ^1^, ^2^ ^]^ The common types of AS events include intron retention (IR), alternative 3′ splice site (A3SS), alternative 5′ splice site (A5SS), exon skipping (ES), and mutually exclusive exons (MXE).^[^ ^2^, ^3^ ^]^ These AS events occur at different frequencies in plants and animals. IR is the most dominant event in plant species, whereas ES is the most prevalent event in animals.^[^ ^4^, ^5^ ^]^ A single gene can produce multiple transcripts via AS, thereby expanding transcriptome complexity and protein diversity.^[^ ^6^, ^7^ ^]^ Consequently, AS is a curial layer in eukaryotic gene regulation at the co‐/post transcription level.

Alternative splicing plays a vital role in plant growth, development, and response to abiotic and biotic stresses.^[^ ^8^, ^9^, ^10^ ^]^ A growing number of studies have demonstrated that AS is involved in the interplay between plants and microbes.^[^ ^9^, ^11^ ^]^ Many AS events have been identified in plant defense‐related genes and *R* genes.^[^ ^5^, ^12^, ^13^, ^14^ ^]^ Moreover, microbes secrete some effectors that target to plant splicing‐related proteins or directly bind to host mRNA to reprogram host mRNA splicing.^[^ ^9^, ^11^, ^15^, ^16^ ^]^ Collectively, these studies indicate that the modulation of host pre‐mRNA AS is a key mechanism by which various microbes regulate plant immunity. However, little is known about the role of host splicing factors in the plant–microbe interaction.

Biomolecular condensates referred to as membrane‐less organelles are formed through a process known as liquid–liquid phase separation (LLPS) and regulate diverse biochemical reactions and cellular functions.^[^ ^17^, ^18^ ^]^ The assembly of LLPS is driven by multivalent interactions mediated mainly by proteins with intrinsically disordered regions (IDRs).^[^ ^17^, ^19^ ^]^ Recently, several phase‐separated proteins have been uncovered to regulate plant immunity by activating the transcription of defense genes, sequestering negative immune regulators, controlling immune gene translation, and modulating hub condensate formation.^[^ ^20^, ^21^, ^22^, ^23^ ^]^ However, the majority of studies involved in the phase separation of plant proteins have focused on the model plant *Arabidopsis*.^[^ ^24^, ^25^, ^26^ ^]^ Despite the identification of a large number of phase‐separated protein candidates in a range of crops, including maize, soybean, and tomato, the potential functions of these phase‐separated protein candidates in crops remain largely unexplored, which has limited relevance to agricultural production.^[^ ^18^, ^26^ ^]^

Irish potato late blight caused by *P. infestans* severely threatens the quality and yield of potatoes and tomatoes worldwide.^[^ ^27^, ^28^ ^]^ Nevertheless, the impact of potential phase‐separated protein on the tomato‐*P. infestans* interaction remains to be elucidated. Here, we show that a tomato SR family protein, SR30, negatively regulates plant immunity. During *P. infestans* infection, SR30 forms nuclear condensates through phase separation and interacts with other spliceosome components. This process suppresses the AS of defense‐related genes, which ultimately leads to increased plant susceptibility. Importantly, the knockout of *SR30* enhances tomato disease resistance against multiple oomycete pathogens. Furthermore, the mechanism by which the up‐regulated expression of SR30 is induced needs further investigations. This study emphasizes the role of biomolecular condensates in regulating plant immunity against oomycete pathogens and provides an example of how to incorporate insights into plant genes related to condensate proteins for resistance breeding in crops.

## 2 Results

### 2.1 Sequence Analysis of SR Family Proteins in Tomato

To identify the SR family proteins in tomato (*Solanum lycopersicum*), we used *A. thaliana* SR proteins to search for their homologous proteins in the latest high‐quality de novo tomato genome SL4.0 with ITAG4.0 annotation. A total of 18 SR proteins were identified in the tomato (Table S1, Dataset S1, File S1, Supporting Information). This result is similar to a recent study in which 19 SR proteins were identified from an old tomato reference genome SL2.4 with ITAG2.4 annotation.^[^ ^29^ ^]^ One of these genes, *Solyc08g006430.3.1*, was previously named *SlRSZ21a*, however, this gene encoded a sarcosine oxidase in the new tomato reference genome, so this gene was removed from our tomato SR proteins list (Table S1, Supporting Information). In addition, in the current tomato SL4.0 genome, there is a 1 bp deletion in the fourth exon in *Solyc08g069120.4.1* and a 4 bp insertion in the first exon in *Solyc06g009060.4.1* when compared to our cloned gene sequences from tomato, so the coding sequences (CDS) of these two genes were corrected accordingly (File S2, Supporting Information). Based on domain prediction, these 18 SR proteins belonged to six designated subfamilies and one SR‐like subfamily (Table S1, Dataset S1, File S1, Supporting Information).

### 2.2 Overexpression of *SR30* Promotes *P. infestans* Infection

**Figure 1:**
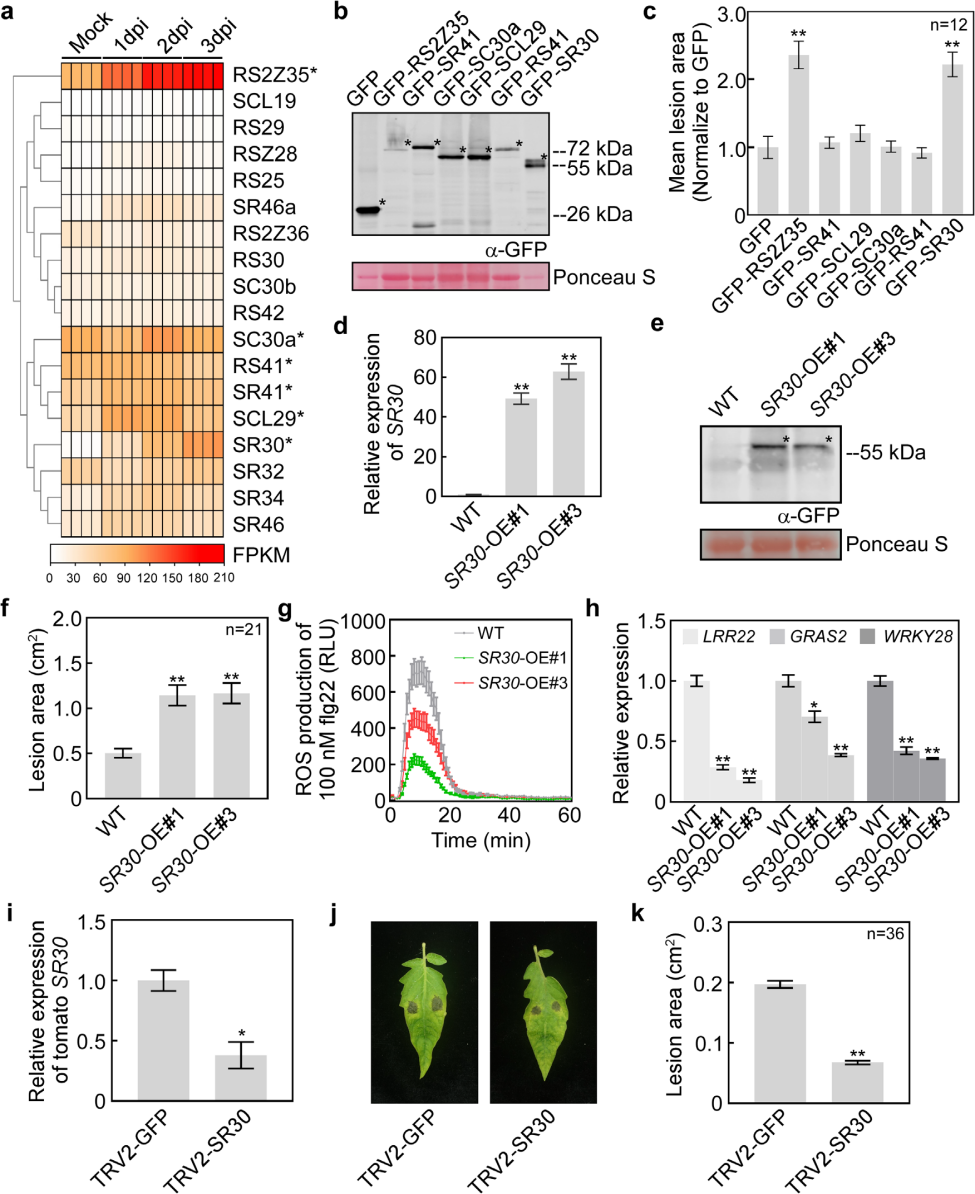
SR30 negatively regulates tomato immunity. a) The heatmap showing the transcript levels of 18 genes encoding SR family proteins in tomato leaves in response to *Phytophthora infestans* infection. The data are derived from RNA‐seq in terms of FPKM (Fragments Per Kilobase of transcript per Million mapped reads) as reported previously.^[^ ^30^ ^]^ Asterisks represent the candidate SR family proteins selected for further immunity function analysis. b) Immuno‐blot analysis of GFP and six SR proteins expressed in *Nicotiana benthamiana* leaves using anti‐GFP antibody. Asterisks indicate the protein bands for each construct. Protein loading was visualized by Ponceau S staining. c) Relative infection lesion areas of *P. infestans* inoculated *N. benthamiana* leaves expressing SR proteins and GFP. The lesion areas were measured at 5 days post‐inoculation (dpi) and then normalized to the GFP control. Data represents the mean with standard errors (SE) (*n* = 12). *P* values were analyzed by Student's *t*‐test (**, *P *< 0.01). The original results of lesion areas for each SR protein are provided in Figure S1c. d) Schematic diagram of the construct used for generating *SR30* transgenic lines. The coding sequence of tomato *SR30* was fused with the N‐terminal GFP, which was driven by the CaMV 35S promoter and followed by a terminator. e) Relative expression of *SR30* in two independent overexpressed (*SR3*0‐OE) transgenic tomato (Micro‐Tom) lines was determined by quantitative reverse transcription‐polymerase chain reaction (qRT‐PCR). Data represents the mean with SE (*n* = 3). *P* values were analyzed by Student's *t*‐test (**, *P *< 0.01). The gene *SlActin* was used as an internal control. f) Protein detection of GFP‐SR30 in two *SR30*‐OE tomato (Micro‐Tom) lines by western blotting using anti‐GFP antibody. The protein bands of GFP‐SR30 are marked with asterisks. Protein loading was visualized by Ponceau S staining. g) Lesion area (cm^2^) of tomato (Micro‐Tom) leaves infected by *P. infestans* at 4 dpi. Data represents the mean with SE (*n* = 21). *P* values were analyzed by Student's *t*‐test (**, *P *< 0.01). h) Detection of the burst of reactive oxygen species in tomato (Micro‐Tom) leaves of WT and two *SR30*‐OE lines in response to 100 nm flg22. The leaf discs were equilibrated in sterilized water in a 96‐well plate for ≈8 h before treatment with flg22. i) Relative expression levels of three tomato defense‐related genes, *LRR22, GRAS2*, and *WRKY28*, were quantified in WT and two *SR30*‐OE transgenic tomato (Micro‐Tom) lines by qRT‐PCR. The gene *SlActin* was used as an internal control. The leaf samples were collected at 1 dpi. Data represents the mean with SE (*n* = 3). *P‐*values were analyzed by Student's *t*‐test (**, *P *< 0.01). j) Relative expression of *SR30* in silencing tomato (Heinz 1706) plants. TRV2‐GFP was used as a negative control. The gene *SlActin* was used as an internal control. Data represents the mean with SE (*n* = 3). *P* values were analyzed by Student's *t*‐test (*, *P *< 0.05). k) Photographs of *P. infestans* infection assay on silenced tomato (Heinz 1706) leaves. *P. infestans* JH19 zoospores were inoculated on the detached tomato leaves 4 weeks after agroinfiltration. l) Lesion area (cm^2^) of *P. infestans*‐inoculated silenced tomato (Heinz 1706) leaves. Data represents the mean with SE (*n* = 36). The lesion areas were measured at 4–5 dpi. *P* values were analyzed by Student's *t*‐test (**, *P *< 0.01).

To determine whether SR family proteins are involved in the tomato‐*P. infestans* interaction, we analyzed the transcript levels of 18 SR protein genes during the tomato leaves response to *P. infestans*.^[^ ^30^ ^]^ This analysis revealed that *SR30*, *RS2Z35*, *SC30a*, *RS41*, *SR41*, and *SCL29* were upregulated during infection, while other SR protein‐coding genes showed low transcription levels (**Figure** **1**a; Figure S1a, Supporting Information). Therefore, these six SR proteins were selected for further investigation.

To determine the immunity function of these six SR proteins, we cloned the CDS of these six SR protein genes (Dataset S1, Supporting Information), expressed them in *Nicotiana benthamiana* (*N. benthamiana*) leaves (Figure 1b), and subsequently performed *P. infestans* infection assays. These infection assay results showed that SR30 and RS2Z35 produced larger lesions than the GFP control, while the other four SR proteins did not (Figure 1c; Figure S1b,c, Supporting Information), suggesting that both SR30 and RS2Z35 have disease susceptibility function. The elevated expression of *SR30* is also triggered by *P. capsici*, whereas *RS2Z35* expression is not altered,^[^ ^31^ ^]^ implying that SR30 may play an important role in tomato response to multiple pathogens. Thus, we continued our studies with SR30.

### 2.3 SR30 Negatively Regulates Tomato Immunity

The SR30 protein belongs to the SR subfamily and contains an RNA recognition motif (RRM), a pseudo‐RNA recognition motif (ψRRM), and a serine/arginine‐rich (SR) domain (File S1, Supporting Information). To further investigate the function of *SR30* in plant immunity, the stable transgenic tomato (Micro‐Tom) lines overexpressing the *GFP‐SR30* (*SR30‐*OE) were generated (Figure 1d; Figure S2a,b, Supporting Information). The *SR30* transcript level was found to be ≈48‐fold and 62‐fold higher in *SR30‐*OE#1 and #3, respectively, when compared to wild‐type (WT) plants (Figure 1e). Moreover, the protein level of SR30 in these two transgenic tomato lines was also confirmed by western blot analysis (Figure 1f).

Subsequently, the *P. infestans* inoculation results showed that overexpression of *SR30* significantly promotes the growth of *P. infestans* lesions compared to WT (Figure 1g; Figure S2c, Supporting Information). To investigate whether the increased expression of *SR30* affects tomato defense response, we also examined the burst of reactive oxygen species (ROS) and the expression level of three known tomato defense‐related genes, *LRR22*, *GRAS2*, and *WRKY28*.^[^ ^32^ ^]^ Upon treatment with flg22, *SR30*‐OE#1 and #3 plants exhibited a lower level of ROS than WT plants (Figure 1h). One day after inoculation with *P. infestans*, the relative expression levels of *LRR22*, *GRAS2*, and *WRKY28* were significantly reduced in both *SR30‐*OE lines compared to WT (Figure 1i). These results suggest that the overexpression of *SR30* suppresses the tomato defense response. Moreover, the transient silencing of *SR30* was performed in tomato (Heinz 1706) plants (Figure 1j). The inoculation assay showed that the silencing of *SR30* significantly enhanced tomato resistance to *P. infestans* (Figure 1k,l). Collectively, these findings indicate that SR30 functions as a negative regulator of plant immunity to *P. infestans* in tomatoes.

### 2.4 SR30 Governs Pre‐mRNA Alternative Splicing at the Genome‐Wide Level

**Figure 2:**
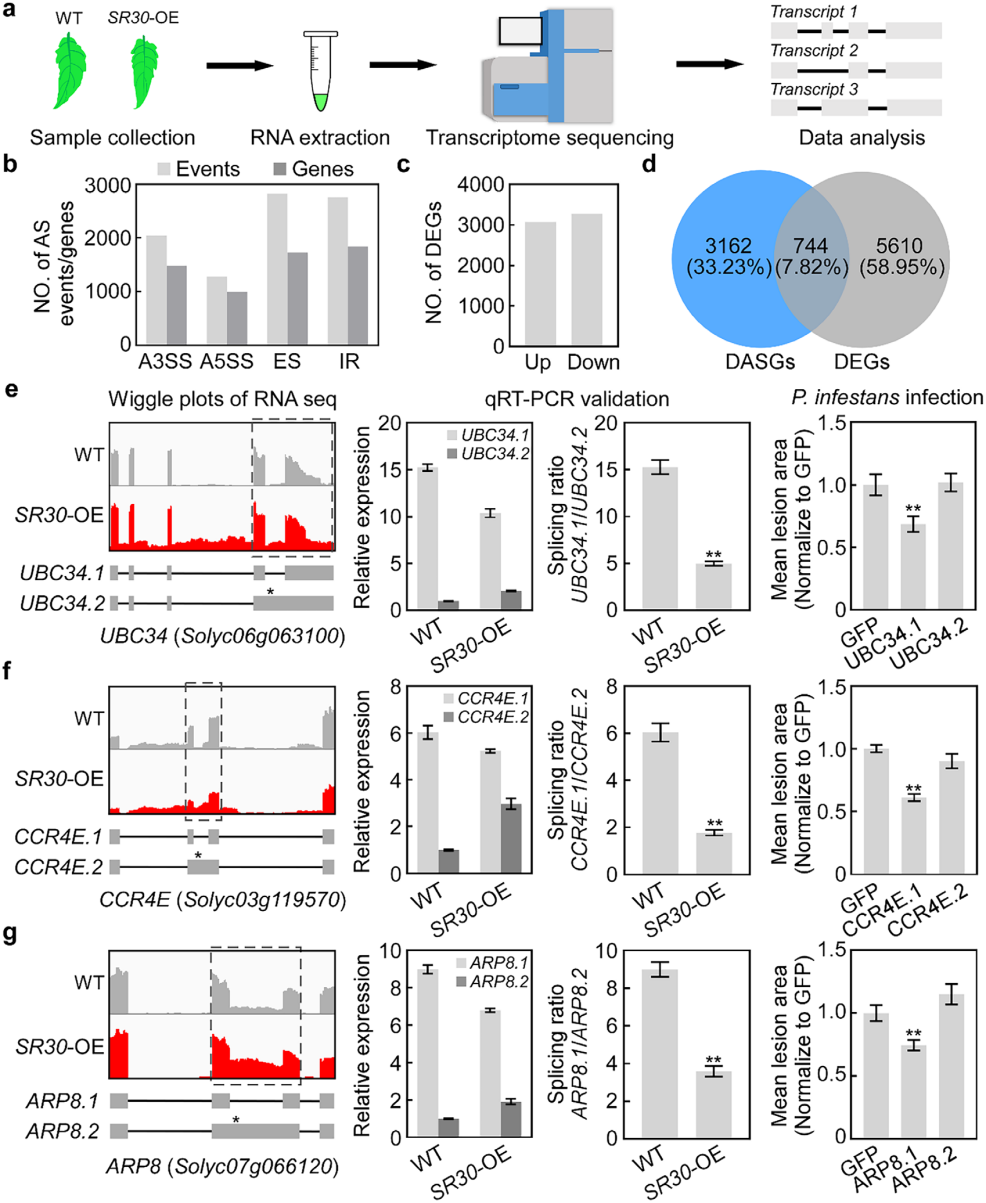
SR30 regulates AS of defense‐related genes in tomato. a) Schematic diagram of transcriptome sequencing using the Illumina platform. The tomato (Micro‐Tom) leaves of *SR30*‐OE and WT plants were sampled for RNA extraction and double‐stranded cDNA library synthesis. All RNA‐seq data were subjected to differential gene expression and differential alternative splicing analysis. b) The number (NO.) of differentially alternatively spliced events and corresponding genes identified from the comparison of *SR30*‐OE versus WT. c) NO. of differentially expressed genes (DEGs) were identified from the comparison of *SR30*‐OE versus WT. Up and down represent upregulated and downregulated DEGs, respectively. d) Venn diagram illustrating the number of differentially alternatively spliced genes (DASGs) and DEGs in the comparison of *SR30*‐OE versus WT. e–g) Validation of the splicing ratio of intron‐spliced and intron‐retained isoforms of three tomato defense‐related genes. The first panel from the left displays wiggle plots of RNA‐seq data for three selected genes, *ubiquitin‐conjugating enzyme 34* (*UBC34*) (e), *carbon catabolite repressor 4E* (*CCR4E*) (f), and *actin‐related protein 8* (*ARP8*) (g), with the schematic gene models of two different transcript isoforms. Asterisks indicate a premature termination codon (PTC). The second panel from the left shows the relative transcript level of two isoforms of three candidate genes using isoform‐specific primers by qRT‐PCR. The third panel shows the splicing ratio (transcript level of intron‐spliced isoform divided by transcript level of intron‐retained isoform) for three defense‐related genes. The tomato ubiquitin gene *UBI* was used as an internal control. The relative transcript level was normalized to the intron‐retained isoform of WT. Data represent the mean with SE (*n* = 3). *P* values were analyzed by Student's *t*‐test (** *P *< 0.01). The last panel shows the functions of different isoforms of three defense‐related genes in plant immunity against *P. infestans*. The GFP was used as a control. Lesion areas were measured at 5 or 6 dpi and then normalized to the GFP control. Data represents the mean with SE (*n* = 20). *P*‐values were analyzed by Student's *t*‐test (** *P *< 0.01).

The SR family proteins are important for the regulation of pre‐mRNA splicing.^[^ ^33^, ^34^ ^]^ To determine the effect of *SR30* on pre‐mRNA AS in tomatoes, RNA sequencing was performed on both WT and *SR30‐*OE plant leaves, followed by a global AS analysis (**Figure** **2**a). The 542127516 reads that passed the quality filters were mapped to the tomato SL4.0 genome with ITAG4.0 annotation and the mapping rate was over 98% (Table S2, Supporting Information). The result of AS analysis revealed a total of 8906 differentially alternative spliced events (DASEs) in the comparison between *SR30*‐OE and WT, consisting of 2042, 1282, 2824, and 2758 events for A3SS, A5SS, ES, and IR, respectively (Figure 2b). These DASEs corresponded to 3906 annotated genes containing 1484, 997, 1732, and 1841 differentially alternative spliced genes (DASGs) for A3SS, A5SS, ES, and IR, respectively (Figure 2b, Dataset S2, Supporting Information). To investigate a global landscape of AS change events, we further performed the percent spliced‐in (PSI) analysis to quantify the changes in these AS events. This result showed a notable alteration in AS when *SR30* was highly expressed in tomatoes (Figure S3a, Supporting Information). Taken together, these results suggest that SR30 regulates the global pre‐mRNA AS in tomatoes.

Moreover, we also analyzed differentially expressed genes (DEGs) between *SR30‐*OE and WT. The results of this analysis showed that 3079 DEGs were significantly upregulated, while 3275 DEGs were significantly downregulated (Figure 2c; Figure S4, Dataset S2, Supporting Information). To further probe the relationship between DEGs and DASGs in the *SR30*‐OE versus WT samples, we performed a pairwise comparison between DEGs and DASGs. The overlap between DEGs and DASGs was low: 7.82% (Figure 2d), indicating that most genes were unique to either DASGs or DEGs.

To validate the DASEs identified in RNA‐seq data, we randomly examined five events by performing quantitative reverse transcription polymerase chain reaction (qRT‐PCR) assays to measure the transcript level of different isoforms. For most IR events, the relative transcript level of the intron‐spliced isoforms decreased, whereas the relative transcript level of the intron‐retained isoforms increased in *SR30‐*OE when compared to WT (Figure 2e–g; Figure S3b, Supporting Information). However, for the *HGNAT* gene, the transcript level of the intron‐spliced isoform *HGNAT.1* did not change, whereas the transcript level of the intron‐retained isoform *HGNAT.2* increased in *SR30*‐OE (Figure S5a, Supporting Information). For the *Glu6* gene, the transcript level of both the intron‐spliced isoform (*Glu6.1*) and the intron‐retained isoform (*Glu6.2*) decreased in *SR30*‐OE (Figure S5b, Supporting Information). To further confirm the splicing analysis results, we examined the splicing ratios (the ratio of intron‐spliced isoforms over intron‐retained isoforms) of these five genes, the splicing ratios of all these five genes were significantly changed (Figure 2e–g; Figure S5a,b, Supporting Information). Taken together, these results indicate that the overexpression of *SR30* induces genome‐wide changes in AS.

### 2.5 SR30 Represses the AS of Tomato Defense‐Related Genes

To investigate how SR30 regulates plant immunity through AS, we analyzed the function of different isoforms of five candidate genes that undergo AS. The *UBC34* is predicted to encode a ubiquitin‐conjugating enzyme. The intron‐spliced transcript isoform *UBC34.1* produces a functional ubiquitin‐conjugating enzyme, whereas the intron‐retained transcript isoform *UBC34.2* produces a truncated protein that lacks the transmembrane domain and disrupts the UBC domain due to a premature termination codon (Figure S6a,S7a, Table S3, Supporting Information). The results of the immunity function analysis showed that overexpression of *UBC34.1* in *N. benthamiana* leaves suppressed *P. infestans* lesion growth compared to GFP control, whereas overexpression of *UBC34.2* had no significant effect on *P. infestans* lesion growth (Figure S7b,c, Supporting Information). This result indicates that the functional isoform of *UBC34* is a positive regulator of plant immunity. The splicing ratio of *UBC34* (*UBC34.1*/*UBC34.2*) was reduced in *SR30*‐OE compared to WT (Figure 2e), suggesting that SR30 regulates plant immunity by suppressing the AS of defense‐related genes.

To further investigate whether AS affects the function of these genes, we examined the function of different isoforms of *CCR4E* and *ARP8* in plant immunity. Two genes, *CCR4E* and *ARP8* are annotated to encode a carbon catabolite repressor and an actin‐related protein, respectively. The *CCR4E.1* transcript produces a functional carbon catabolite repressor and the *ARP8.1* transcript produces a functional actin‐related protein, while both the *CCR4E.2* and *ARP8.2* transcripts undergo IR, resulting in the production of truncated proteins (Figure S6b,c, Supporting Information). The *P. infestans* infection assays showed that overexpression of *CCR4E.1* and *ARP8.1* in *N. benthamiana* leaves significantly compromised *P. infestans* lesion growth, whereas overexpression of *CCR4E.2* and *ARP8.2* did not affect *P. infestans* lesion growth (Figure S7a–c, Supporting Information). These results demonstrate that the functional isoforms of *CCR4E* and *ARP8* are positive regulators of plant immunity against *P. infestans*. The qRT‐PCR data show that the splicing ratios of *CCR4E* (*CCR4E.1*/*CCR4E.2*) and *ARP8* (*ARP8.1*/*ARP8.2*) were decreased in *SR30*‐OE plants compared to WT (Figure 2f,g), which means that the production of functional isoforms of *CCR4E* and *ARP8* was reduced in *SR30*‐OE plants. In addition, the inoculation results show that overexpression of *HGNAT.1* and *Glu6.1* in *N. benthamiana* leaves did not affect the lesion growth of *P. infestans* (Figure S5c,d, Supporting Information), so these two genes are not used for further investigation. Collectively, these results demonstrate that SR30 suppresses the AS of defense‐related genes to repress plant immunity.

### 2.6 SR30 Forms Nuclear Condensates via Phase Separation

**Figure 3:**
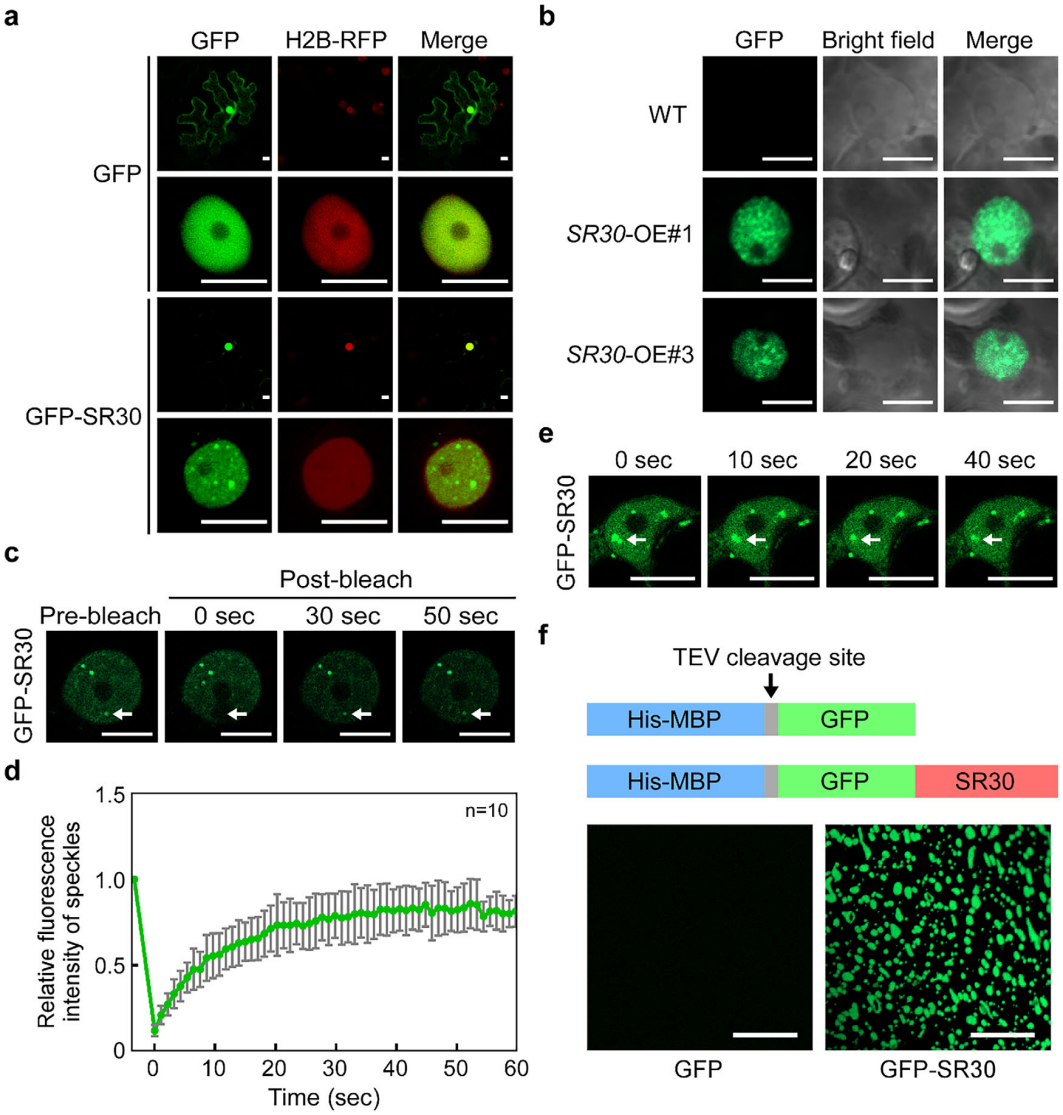
SR30 forms nuclear condensates through LLPS. a) Subcellular localization of GFP and GFP‐SR30. GFP and GFP‐SR30 were transiently expressed in transgenic *N. benthamiana* leaves expressing a nuclear marker (H2B‐RFP) for 48 h before imaging by a confocal microscope (20× and 63× objective). Scale bars represent 10 µm. b) Subcellular localization of GFP‐SR30 in *SR30*‐OE transgenic tomato leaves. Scale bars represent 5 µm. c) FRAP of GFP‐SR30 expressed in epidermal cells of *N. benthamiana* leaves. The white arrows indicate the photobleached nuclear condensate. Scale bars represent 10 µm. d) Relative fluorescence recovery curves of photobleached GFP‐SR30. Data were normalized by the web‐based tool easyFRAP. Data represents the mean with standard deviation (SD) (*n* = 10). e) Fusion of two independent nuclear condensates of GFP‐SR30. The arrow indicates two nuclear condensates undergoing fusion. Scale bars represent 10 µm. f) Droplet formation of the purified protein GFP‐SR30 in vitro. The top cartoon shows the schematic diagram of His‐MBP‐GFP and His‐MBP‐GFP‐SR30 recombinant proteins. TEV protease was used to remove the His‐MBP tag. The bottom images show the droplet formation of 1 µM GFP‐SR30 in vitro in the presence of 150 mm NaCl. The GFP protein was used as a control. Scale bars represent 10 µm.

To further address how SR30 modulates tomato immunity, we tracked its subcellular localization in *N. benthamiana* with H2B‐RFP using GFP‐tagged translational fusions. The result showed that GFP‐SR30 formed discrete nuclear condensates of various sizes, while GFP did not form any condensates in the entire cell nucleus (**Figure** **3**a). The subcellular localization of nuclear condensates was also observed in the *SR30*‐OE tomato leaf nucleus (Figure 3b). In addition, the GFP‐SR30 colocalized with AtSR45‐RFP and AtSR34‐RFP, proteins that have been shown to form nuclear speckles (Figure S8, Supporting Information).^[^ ^35^ ^]^ Furthermore, AtSR45 has been demonstrated to function as a splicing factor in an in vitro splicing assay^[^ ^36^ ^]^ and it localizes to nuclear speckles with other spliceosomal proteins including U2AF^35^a and U2AF^35^b.^[^ ^37^, ^38^, ^39^ ^]^ Thus, we infer that the SR30 is probably located in the nuclear speckles that store splicing factors. Condensates are typically formed through liquid–liquid phase separation (LLPS).^[^ ^17^, ^19^ ^]^ To determine the molecular dynamics of the SR30 protein, we performed the fluorescence recovery after photobleaching (FRAP) assay. The result showed that the nuclear condensate of GFP‐SR30 recovered rapidly within one minute after photobleaching (Figure 3c,d, Movie S1, Supporting Information). Furthermore, two nuclear condensates of SR30 were able to fuse into a single condensate (Figure 3e, Movie S2, Supporting Information). These results suggest that SR30 has liquid‐like property and internally exchanges materials with the nucleoplasm. To determine whether SR30 independently undergoes phase separation in vitro, we expressed and purified the recombinant protein GFP‐SR30 from *Escherichia coli* (*E. coli*) cells (Figure S9, Supporting Information). We observed that GFP‐SR30 was able to form spherical droplets in vitro, whereas GFP was not (Figure 3f). Taken together, these data suggest that SR30 undergoes phase separation to form condensates in vivo and in vitro.

### 2.7 Phase Separation of SR30 Is dependent on Intrinsically Disordered Regions

**Figure 4:**
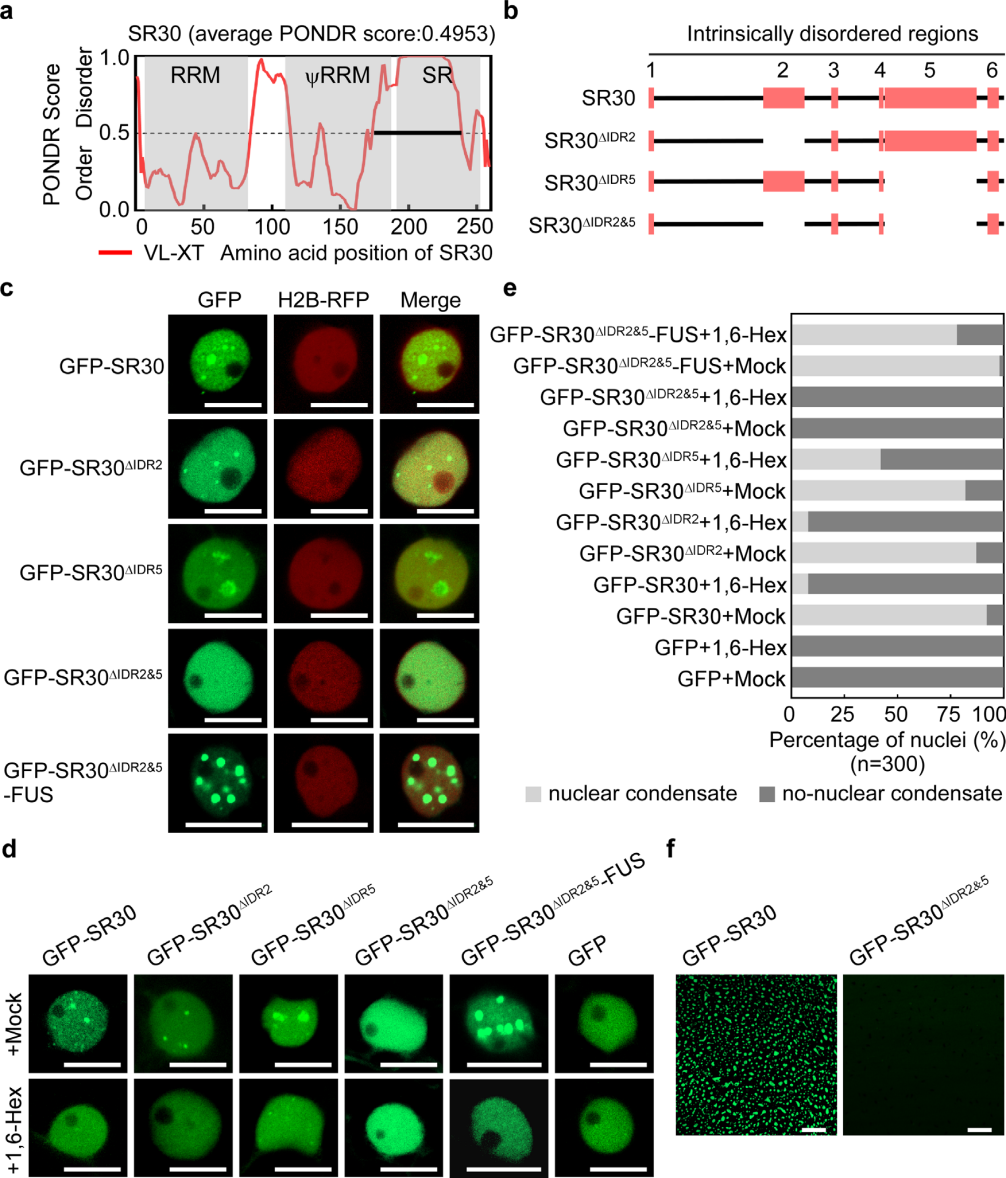
LLPS of SR30 is driven by IDRs. a) Predicted intrinsically disordered regions (IDRs) of SR30 using PONDR. Three gray boxes represent the region of RNA recognition motif (RRM), pseudo‐RNA recognition motif (ψRRM), and serine/arginine‐rich (SR) domain of SR30, respectively. b) Schematic diagrams of three designed IDR‐deletion mutants of SR30. c) Subcellular localization of IDR‐deletion variants of SR30 and complementary mutant SR30^ΔIDR2&5^‐FUS. All mutants were expressed in nuclear marker H2B‐RFP transgenic *N. benthamiana* leaves for 48 h before imaging using a confocal microscope. Scale bars represent 10 µm. d) Subcellular localization of IDR‐deletion mutants of SR30 and SR30^ΔIDR2&5^‐FUS under 1,6‐hexanediol (1,6‐Hex) treatment. All proteins were expressed in *N. benthamiana* for 36–48 h. For mock or 1,6‐Hex treatment, leaf tissues were infiltrated with Milli‐Q water or 5% 1,6‐Hex before observation. Scale bars represent 10 µm. e) Percentage of nuclei containing nuclear condensates of IDR‐deletion mutants and complementary mutant SR30^ΔIDR2&5^‐FUS treated with 1,6‐Hex compared to mock control. A total of 300 nuclei were calculated for each treatment. f) Droplet formation of 1 µM GFP‐SR30 and GFP‐SR30^ΔIDR2&5^ in vitro in the presence of 150 mm NaCl. Scale bars represent 10 µm.

The multivalent interactions driven by intrinsically disordered regions (IDRs) are critical for protein LLPS.^[^ ^17^, ^19^ ^]^ SR30 contains six different IDRs via PONDR prediction (**Figure** **4**a,b; Figure S10a, Supporting Information). Among these, we focused on two major IDRs, named IDR2 and IDR5, which contain more disordered amino acids (Figure 4a; Figure S10a, Supporting Information). The IDR2 is predominantly located between the RRM and the ψRRM domain (Figure 4a). Most of IDR5 overlaps with the SR domain (Figure 4a). Moreover, we found that IDR2 and IDR5 are localized in the linker part of SR30 based on the 3D structure (Figure S10b, Supporting Information).

To determine whether the phase separation of SR30 is mediated by IDR, we examined the nuclear condensate formation of different IDR‐deletion variants (Figure 4b; Figure S11a, Supporting Information). Like GFP‐SR30, two IDR‐deletion variants, GFP‐SR30^ΔIDR2^ and GFP‐SR30^ΔIDR5^, were also primarily located in the cell nucleus and formed nuclear condensates (Figure 4c; Figure S11b, Supporting Information). However, in terms of the nuclear condensate size, GFP‐SR30^ΔIDR2^ showed a notable reduction compared to GFP‐SR30, whereas GFP‐SR30^ΔIDR5^ exhibited an increase (Figure S11c, Supporting Information), suggesting that IDR regulates the phase separation ability of SR30 in vivo. Notably, we found that only the GFP‐SR30^ΔIDR2&5^ mutant failed to form nuclear condensates and was evenly distributed throughout the nuclei (Figure 4c). These results indicate that both IDR2 and IDR5 are required for the nuclear condensate formation of SR30 in vivo. To further determine the important role of IDR in SR30′s phase separation, we tried to rescue the phase separation of SR30^ΔIDR2&5^ by introducing a low‐complexity domain (LCD) of the RNA‐binding protein FUSED IN SARCOMA (FUS) to its C terminal (SR30^ΔIDR2&5^‐FUS).^[^ ^23^, ^40^, ^41^ ^]^ As we expected, GFP‐SR30^ΔIDR2&5^‐FUS re‐formed nuclear condensates and its condensate size was larger than GFP‐SR30 (Figure 4c; Figure S11b,c, Supporting Information), suggesting LCD of FUS can recover the phase separation ability of SR30^ΔIDR2&5^.

To support this finding, we treated GFP‐SR30 and its IDR‐deletion variants with 1,6‐hexanediol (1,6‐Hex), which dissolves biomolecular condensates by disrupting the hydrophobic interaction in vivo.^[^ ^42^, ^43^ ^]^ For GFP‐SR30, GFP‐SR30^ΔIDR2^, GFP‐SR30^ΔIDR5^, and GFP‐SR30^ΔIDR2&5^‐FUS, the nuclear condensates partially disappeared when treated with 5% 1,6‐Hex compared to mock, and the number of nuclei with nuclear condensates declined in the presence of 1,6‐Hex; however, the treatment of 1,6‐Hex had no effect on GFP‐SR30^ΔIDR2&5^ (Figure 4d,e). This result reveals that the nuclear condensates formed by GFP‐SR30, GFP‐SR30^ΔIDR2^, GFP‐SR30^ΔIDR5^, and GFP‐SR30^ΔIDR2&5^‐FUS are sensitive to 1,6‐Hex except for GFP‐SR30^ΔIDR2&5^, likely due to LLPS. In addition, to determine whether IDR2 and IDR5 are also required for the phase separation of SR30 in vitro, we also produced the purified recombinant protein GFP‐SR30^ΔIDR2&5^ (Figure S9, Supporting Information). However, it failed to form spherical droplets like GFP‐SR30 in vitro (Figure 4f), suggesting the vital role of IDRs in the SR30′s phase separation in vitro. Taken together, these data demonstrate that the phase separation of SR30 is dependent on its IDR2 and IDR5.

### 2.8 Phase Separation of SR30 Determines Its Function

**Figure 5:**
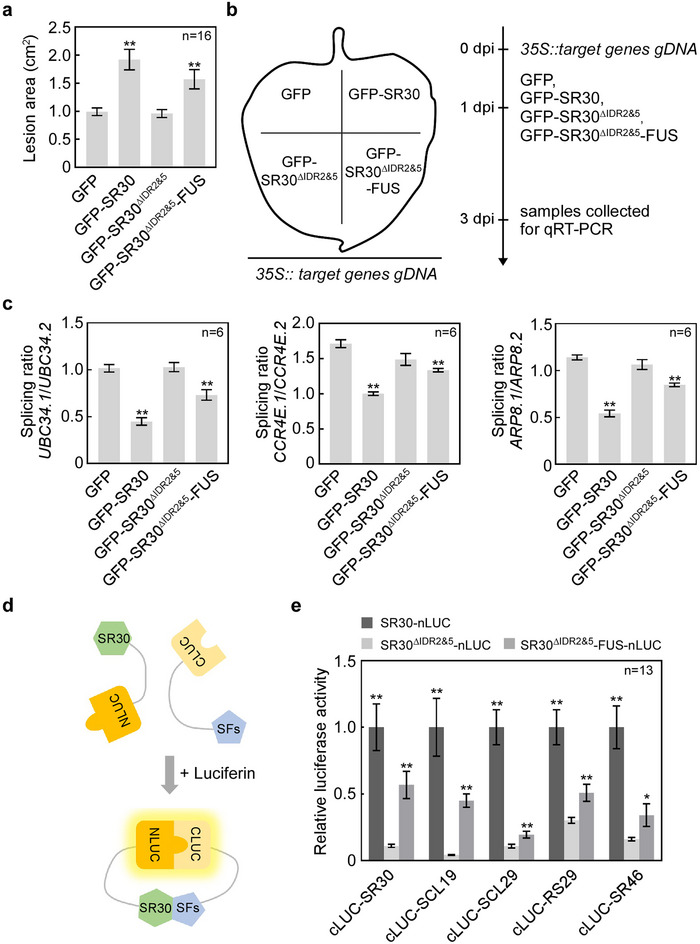
SR30 regulates the AS of defense genes through LLPS to perform susceptible functions. a) Lesion area (cm^2^) of *N. benthamiana* leaves expressing GFP, GFP‐SR30, GFP‐SR30^ΔIDR2&5^, and GFP‐SR30^ΔIDR2&5^‐FUS at 5 dpi. Data represents the mean with SE (*n* = 16). *P*‐values were analyzed by Student's *t*‐test (**, *P *< 0.01). b) Schematic diagram of the procedure to validate the effect of SR30′s phase separation mutants on the AS of target genes. Step 1, the *Agrobacterium tumefaciens* strains carrying *35S::target genes gDNA* (*35S::SlUBC34/SlCCR4E/SlARP8 gDNA*) were infiltrated into the whole leaves of *N. benthamiana*. Step 2, GFP, GFP‐SR30, GFP‐SR30^ΔIDR2&5^, and GFP‐SR30^ΔIDR2&5^‐FUS were expressed in different regions of the same *N. benthamiana* leaf expressing *35S:: target genes gDNA*, respectively, at one‐day post‐infiltration (dpi). Step 3, the *N. benthamiana* leaves expressing different proteins were collected, respectively, and the total RNA of all treatment samples was extracted to perform qRT‐PCR assays. c) The splicing ratio of two different isoforms of *UBC34*, *CCR4E*, and *ARP8* under treatment with GFP, GFP‐SR30, GFP‐SR30^ΔIDR2&5^, and GFP‐SR30^ΔIDR2&5^‐FUS by qRT‐PCR, respectively. The gene *NbActin* was used as an internal control. For each target gene, the relative transcript level of two different isoforms of different treatments is normalized to the transcript level of the intron‐spliced isoform. Data represent the mean with SE (*n* = 6). *P* values were analyzed by Student's *t*‐test (**, *P *< 0.01). This experiment was repeated twice with similar results. d) Schematic diagram of the split‐LUC assays to test the interactions between SR30 and other splicing factors (SFs). SR30, SR30^ΔIDR2&5^, and SR30^ΔIDR2&5^‐FUS were inserted into the pICH86988‐nLUC vector, while other splicing factors were inserted into the pCAMBIA1300‐cLUC vector. If two test proteins interact with each other, the luciferase will emit fluorescence in the presence of luciferin. e) Relative LUC activity of the interaction between SR30, SR30^ΔIDR2&5^, and SR30^ΔIDR2&5^‐FUS with other tomato splicing factors by split‐LUC assays, respectively. Leaf discs were used to measure the luminescence 48 h after co‐expression of the indicated proteins. The relative LUC activity of SR30^ΔIDR2&5^ and GFP‐SR30^ΔIDR2&5^‐FUS with each splicing factor was normalized to SR30 with the same splicing factor. Data represents the mean with SE (*n* = 13). *P‐*values were analyzed by Student's *t*‐test (*P < 0.05, **P < 0.01).

To determine the relationship between the disease susceptibility function of SR30 and its phase separation ability, we performed the immunity functional analysis for the SR30^ΔIDR2&5^ variant. The *P. infestans* inoculation assay showed that the overexpression of GFP‐SR30^ΔIDR2&5^ in *N. benthamiana* did not promote *P. infestans* infection compared to GFP control (**Figure** **5**a; Figure S12, Supporting Information). Conversely, overexpression of GFP‐SR30^ΔIDR2&5^‐FUS significantly promoted *P. infestans* lesion growth similar to GFP‐SR30 (Figure 5a; Figure S12, Supporting Information). This result indicates that the phase separation mediated by IDRs is required for the disease susceptibility function of SR30. Next, we investigated the function of SR30′s phase separation in modulating the AS of tomato defense‐related genes. We first transiently expressed *35S::SlUBC34 gDNA* in *N. benthamiana* leaves; one day later, we expressed GFP, GFP‐SR30, GFP‐SR30^ΔIDR2&5^, and GFP‐SR30^ΔIDR2&5^‐FUS in the same *N. benthamiana* leaf expressing *35S::SlUBC34 gDNA*; finally, we collected leaf samples with different treatments, extracted total RNA, and measured the splicing ratio of *SlUBC34* (*UBC34.1*/*UBC34.2*) by qRT‐PCR (Figure 5b). The result showed that overexpression of GFP‐SR30 reduced the splicing ratio of *SlUBC34* compared to the GFP control, whereas overexpression of GFP‐SR30^ΔIDR2&5^ did not affect the splicing ratio, and notably, overexpression of GFP‐SR30^ΔIDR2&5^‐FUS restored the repression for the AS of *SlUBC34* (Figure 5c; Figure S13, Supporting Information). To further substantiate this finding, we also examined another two genes, *SlCCR4E* and *SlARP8*, using the identical approach. Similar to *SlUBC34*, overexpression of GFP‐SR30 and GFP‐SR30^ΔIDR2&5^‐FUS resulted in a decrease in the splicing ratio of *SlCCR4E* (*CCR4E.1*/*CCR4E.2*) and *SlARP8* (*ARP8.1*/*ARP8.2*), while overexpression of GFP‐SR30^ΔIDR2&5^ did not affect their splicing ratio (Figure 5c; Figure S13, Supporting Information). Thus, these results suggest that the phase separation driven by IDRs is required for SR30 to modulate the AS of tomato defense‐related genes.

To further investigate how SR30 suppresses pre‐mRNA splicing, we tested the interactions between SR30, SR30^ΔIDR2&5^, SR30^ΔIDR2&5^‐FUS and eight splicing factors (SR30, SR41, SCL19, SCL29, RS29, RS30, SR46, U1‐70K) using the Split‐LUC assay (Figure 5d; Figure S14a, Supporting Information). This result showed SR30, SR30^ΔIDR2&5^ and SR30^ΔIDR2&5^‐FUS cannot interact with SR41, RS30, and U1‐70K (Figure S14a, Supporting Information). Besides, we also found that the interactions between SR30^ΔIDR2&5^ and the remaining five splicing factors (SR30, SCL19, SCL29, RS29, and SR46) were attenuated compared to SR30, whereas GFP‐SR30^ΔIDR2&5^‐FUS partially recovered the interacting ability (Figure 5e; Figure S14a, Supporting Information). Moreover, the result of the yeast two‐hybrid assay showed that SR30 also interacted with these five splicing factors, whereas SR30^ΔIDR2&5^ only exhibited interactions with SR30, SCL19, RS29, and SR46, with each of these interactions being notably attenuated (Figure S14b, Supporting Information). Due to the self‐activation phenomenon of SR30^ΔIDR2&5^‐FUS in yeast, we cannot verify its interaction with splicing factors in yeast. These results demonstrate a reduced interaction propensity of SR30^ΔIDR2&5^, thereby providing preliminary mechanistic insights into how SR30^ΔIDR2&5^ attenuates the suppression of pre‐mRNA splicing.

### 2.9 Loss of *SR30* Function Enhances Tomato Resistance to Multiple Oomycetes Pathogens by Promoting AS of Defense‐Related Genes

**Figure 6:**
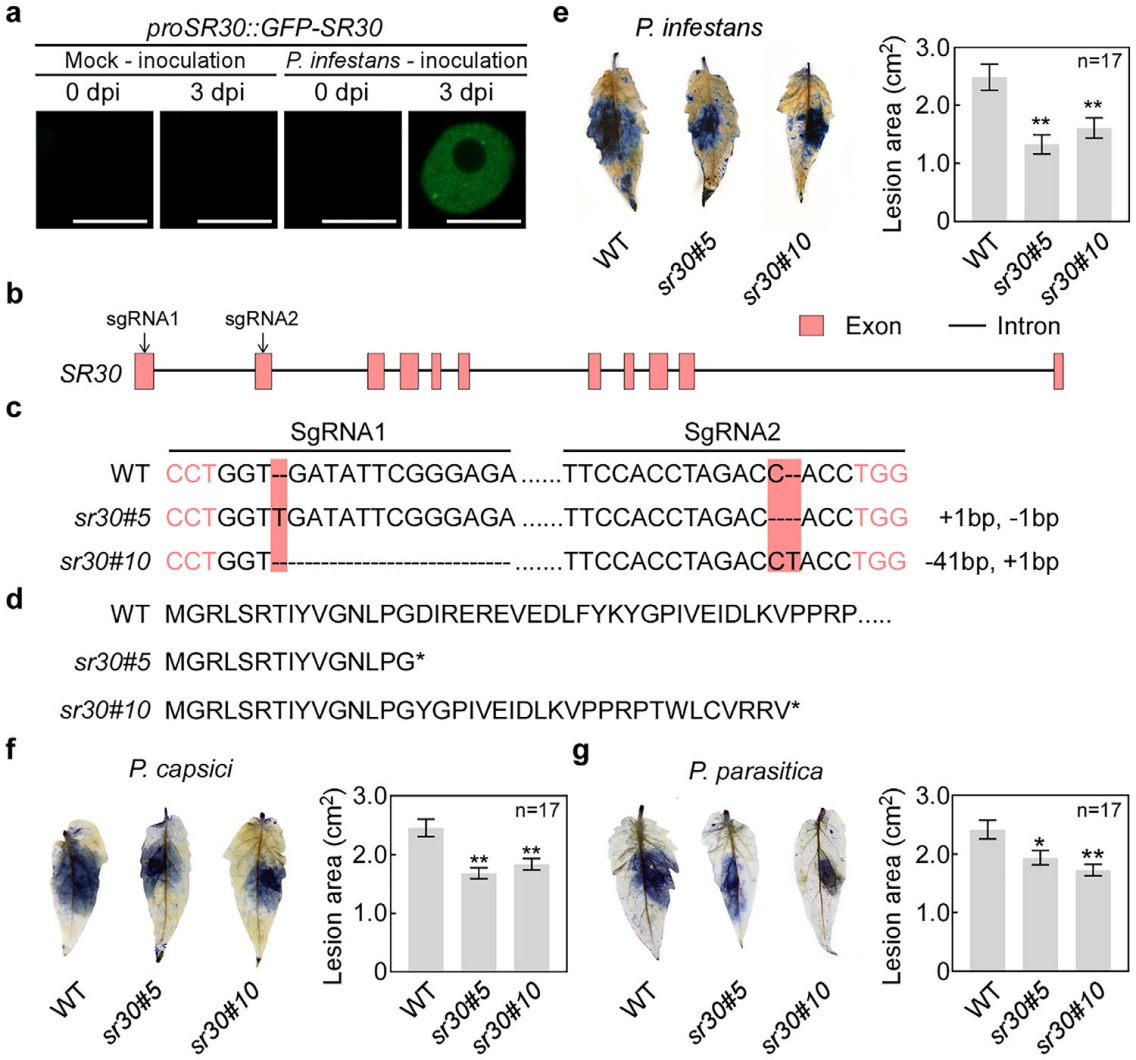
Knockout of *SR30* enhances tomato resistance against three oomycetes pathogens. a) Formation of nuclear condensates of GFP‐SR30 driven by the native *SR30* promoter was examined under *P. infestans* infection. The agrobacterium carrying *proSR30::GFP‐SR30* was infiltrated into *N. benthamiana* leaves. The infiltrated areas of leaves were inoculated with either mock (Milli‐Q water) treatment or *P. infestans* zoospores at one‐day post‐agroinfiltration. Leaf tissues were sampled at zero‐ and three‐days post‐inoculation of mock or *P. infestans*, observed under a confocal microscope, and photographed using ZEN software. Scale bars represent 10 µm. b) Schematic diagram of two small guide RNAs (sgRNAs) designed to knockout *SR30*. Arrows represent two designed sgRNA positions in the *SR30* gene. Red boxes represent exons and black lines represent introns. c) Genomic DNA (gDNA) sequence of *SR30* in two different *sr30* mutants. The red nucleobases indicate the positions of two protospacer adjacent motifs (PAM). The red regions indicate the differences of *SR30* gDNA sequences in two *sr30* mutants compared to WT by sequence alignment. d) Protein sequences of SR30 in *sr30* mutants and WT tomato. The asterisk indicates the stop codon. e–g) The image showed that knockout *SR30* in tomatoes suppressed the lesion growth of *P. infestans* (e), *P. capsici* (f), and *P. parasitica* (g). For *P. infestans*, detached leaves of *sr30* and WT were inoculated with zoospores. Infected leaves were stained with trypan blue and photographed 4 dpi. For *P. capsici* and *P. parasitica*, detached leaves of *sr30* and WT were inoculated with mycelium disc. Infected leaves were stained with trypan blue and photographed 2 dpi. The columns showed the lesion areas of tomato (Micro‐Tom) leaves infected by three *Phytophthora* pathogens. For (e–g), data represent the mean with SE (*n* = 17). *P* values were analyzed by student′s *t‐*test (*P < 0.05, **P < 0.01).

To further investigate the role of SR30 in the tomato‐*P. infestans* interaction, we monitored the nuclear condensate formation of GFP‐SR30 driven by its native promoter after inoculation with *P. infestans*. The results showed that nuclear condensates were observed upon infection with *P. infestans* while no signal was observed in control samples (**Figure** **6**a). This result indicates that SR30 can undergo LLPS to form nuclear condensates during *P. infestans* infection. Next, we designed small guide RNAs and generated two knockout mutants (*sr30*) through CRISPR‐Cas9‐mediated genome editing in Micro‐Tom tomato plants (Figure 6b,c). The *sr30* mutants only translated into a truncated protein that did not contain IDR2 and IDR5, which means a loss of phase separation ability (Figure 6d). Our current data indicate no statistically significant differences in plant height or single fruit weight between *sr30*#5 and WT plants (Figure S15, Supporting Information).

**Figure 7:**
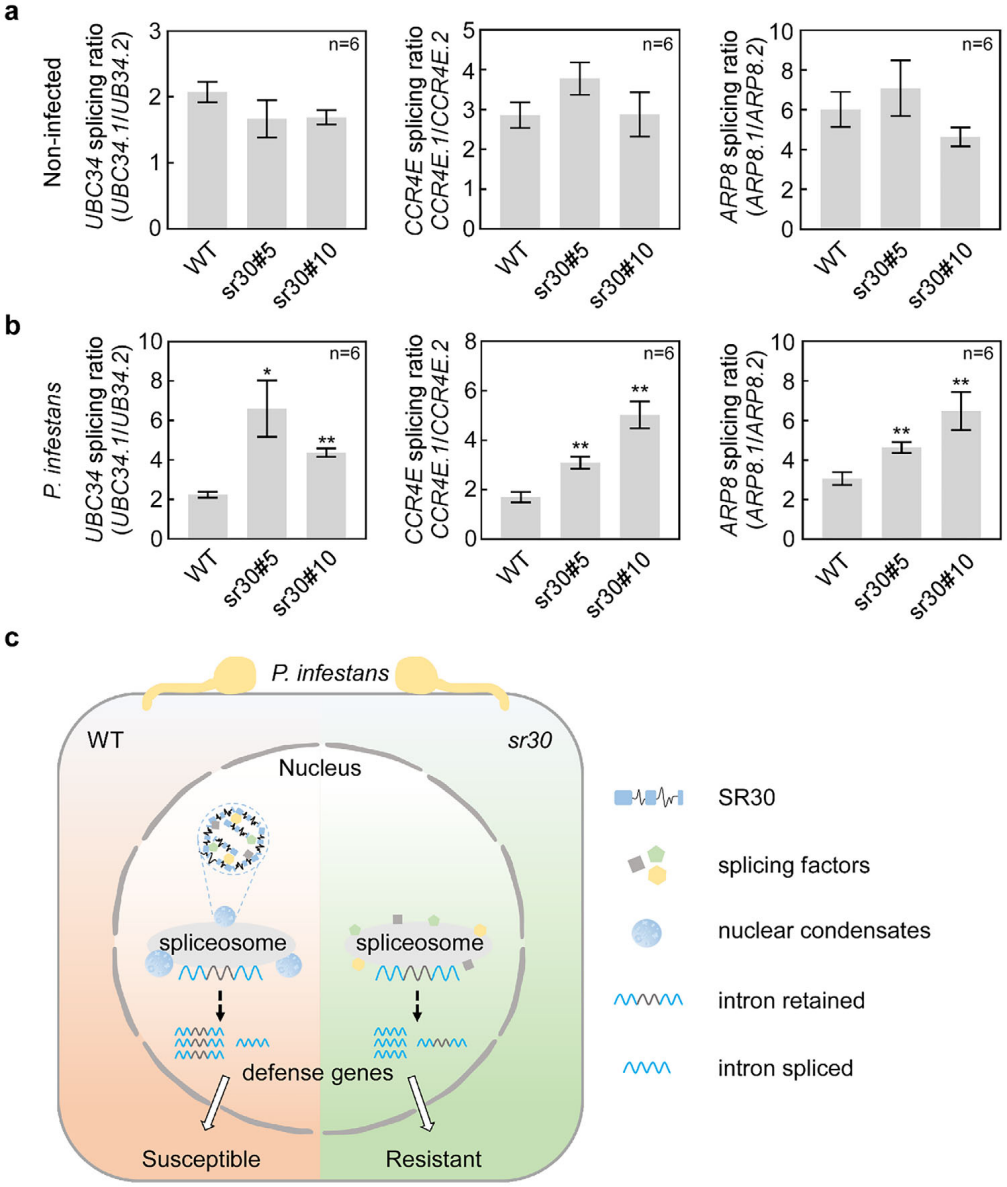
The knockout of *SR30* promotes the AS of defense‐related genes during pathogen infection. a,b) Splicing ratio of *UBC34*, *CCR4E*, and *ARP8* in *sr30* tomato mutants during the non‐infected condition (a) and *P. infestans* infection (b). The infected leaves samples were collected at 2 dpi with *P. infestans*. The tomato ubiquitin gene *UBI* was used as an internal control. Data represent the mean with SE (*n *= 6). *P*‐values were analyzed by Student's *t*‐test (*P < 0.05, **P < 0.01). For (a,b), each experiment was repeated twice with similar results. c) A proposal working model of SR30 negatively regulating tomato immunity. During *P. infestans* infection, the increased concentration of SR30 promotes the formation of nuclear condensates via LLPS where SR30 sequestrates other splicing factors and compromises spliceosome function in WT. As a result, the AS of defense‐related genes is suppressed and the plant becomes more susceptible. Conversely, the truncated SR30 cannot form condensates to destabilize defense genes AS in *the sr30* mutant. Consequently, the loss of function of *SR30* confers tomato resistance to *P. infestans*.

Inoculation assay results showed that the *P. infestans* lesion growth was significantly reduced in the *sr30* mutant compared to WT (Figure 6e). The knockout of *SR30* also exhibited a significant increase in disease resistance to both *P. capsici* and *P. parasitica* compared to WT (Figure 6f,g), but it did not enhance resistance against *Botrytis cinerea* and *Meloidogyne incognita* (Figure S16, Supporting Information). These results suggest that the inactivation of *SR30* enhances the disease resistance of tomatoes against different oomycetes pathogens. In addition, we examined the AS changes of defense genes in *sr30* mutants. Notably, there was no significant difference in the AS of the three examined defense genes between *sr30* mutants and WT plants under non‐infected conditions (**Figure** **7**a). However, during *P. infestans* infection, a marked increase in splicing ratios of three defense genes was observed in *sr30* mutants when compared to WT (Figure 7b; Figure S17, Supporting Information). These results indicate that the inactivation of the *SR30* improves tomato resistance by promoting defense‐related gene AS (Figure 7c).

## 3 Discussion

The AS of host pre‐mRNA is widespread in plant–microbe interactions.^[^ ^9^ ^]^ However, little is known about how the host splicing factors are involved in the plant–pathogen interaction. In the present study, we reveal that splicing factor SR30 negatively regulates tomato immunity against *P. infestans* by interfering with the defense‐related genes AS in a phase separation manner. In WT tomato, the transcription of *SR30* is inducible during *P. infestans* infection. As the concentration of SR30 increases, it forms the nuclear condensates through phase separation and isolates other splicing factors from the spliceosome, thereby suppressing the AS of defense‐related genes. Finally, this process results in an increased disease susceptibility of the plant (Figure 7c). In contrast, in the absence of SR30, splicing machinery normally works and produces more functional transcripts of defense‐related genes, which enhances disease resistance (Figure 7c). Notably, the loss of function of *SR30* confers broad‐spectrum disease resistance to oomycetes pathogens in tomatoes.

RNA splicing is an important process of gene expression regulation in most eukaryotic organisms. Recent transcriptome analysis based on the knockdown of many spliceosome components reveals that different core spliceosome components have different and specialized regulatory functions for the pre‐mRNA splicing network.^[^ ^44^ ^]^ Among 305 splicing factors, the knockdown of splicing factors involved in the early steps of 5′SS or 3′SS more frequently affects ES events, while the knockdown of splicing factors involved in the late stages of spliceosome assembly and activation more frequently affects IR events.^[^ ^44^ ^]^ In this study, we found that the overexpression of *SR30* leads to more than 2000 ES and IR events, implying that SR30 has a crucial role in splice site selection and spliceosome assembly.

In the pathogen‐mediated modulation of the host mRNA AS process, the effector is a sharp weapon.^[^ ^11^ ^]^ In addition to effectors, host splicing factors have equally important roles in plant immunity mediated by AS against pathogens.^[^ ^45^, ^46^, ^47^ ^]^ For example, plant immunity suppressor AtSKRP represses intron removal at genes promoting susceptibility to *P. capsici*.^[^ ^47^ ^]^ We provided evidence that increased expression of *SR30* suppresses the AS of tomato defense‐related genes *UBC34*, *CCR4E*, and *ARP8* to enhance plant susceptibility (Figure 2). Moreover, a recent study shows that splicing factor SR45 is a negative regulator of the mycorrhiza symbiosis.^[^ ^16^ ^]^ These findings, together with our results, indicate that the AS‐plant immunity mediated by splicing factors plays a key role in the plant response to pathogens or benefic microbes. Unlike pathogens, perhaps the disease susceptibility function of splicing factors such as SR30 may be useful for some beneficial microbes to colonize in host plant. The increased transcription level of *SR30* was induced by two pathogens, thus whether the expression of *SR30* is also induced by symbiotic microbes or not needs further investigations.

Although splicing factors regulate plant immunity by affecting the splicing of plant immunity genes, how they regulate these genes AS during plant–microbe interaction remains elusive. Recently, emerging evidence has shown that the biomolecular condensates organize multiple steps of the RNA life cycle, including RNA splicing.^[^ ^48^ ^]^ For example, the formation of phase‐separated nuclear condensates is essential for the RNA‐binding protein HRLP to regulate *FLC* splicing with SR45 in *Arabidopsis*.^[^ ^49^ ^]^ Here, we show that SR30 undergoes phase separation to form nuclear condensates (Figures 3, 4). This is consistent with the previous prediction that tomato SR30 contains a putative region that could drive phase separation (File S3, Supporting Information).^[^ ^24^ ^]^ Importantly, the regulation of SR30 on the AS of genes is dependent on its phase separation. This result is consistent with the recent demonstration that nuclear speckles have higher spliceosome concentrations and much of the splicing occurs closer to the speckles.^[^ ^50^ ^]^ These studies highlight the role of condensates in regulating pre‐mRNA splicing. In addition, the following lines of evidence strongly suggest that SR30 is most likely to form condensates resembling nuclear speckles: i) although GFP and GFP‐SR30 are expressed using the same promoter, only GFP‐SR30 forms the speckle‐like nuclear condensates (Figure 3a), ii) SR proteins form the typical nuclear speckles^[^ ^51^, ^52^, ^53^ ^]^ and the *Arabidopsis* ortholog of tomato SR30 has also been shown to localize to speckles.^[^ ^54^ ^]^ Notably, the SR30 co‐localized with AtSR45, a known splicing factor that interacts with other spliceosomal proteins in speckles,^[^ ^36^, ^37^, ^38^, ^39^ ^]^ and AtSR34 (Figure S8, Supporting Information), iii) the formation SR30 condensates require the intrinsically disordered region (IDR), a domain that is known to drive liquid–liquid phase separation in splicing factors and other proteins (Figure 4c), iv) SR30 condensates exhibit known liquid‐like properties of condensates (e.g., a fusion of condensates, Figure 3e), v) as with other condensate resident proteins, SR30 continuously moves from nucleoplasm to condensates as shown in FRAP studies (Figure 3c,d), vi) only GFP‐SR30 (not GFP) forms condensates both in vitro and in vivo (Figures 3, 4). However, our current data do not exclude the possibility that SR30 nuclear condensates are distinct from nuclear speckles due to certain technological limitations. Plant immunofluorescence labeling with SR30‐specific antibodies to determine the localization of endogenous SR30 in speckles or colocalization studies of SR30 fusion proteins with other endogenous splicing proteins using animal cell research systems may help to address this question in the future.

LLPS‐mediated condensates recently reported have been involved in the regulation of plant immunity in distinct ways.^[^ ^20^, ^21^, ^23^, ^43^ ^]^ For example, during ETI (effector‐triggered immunity), the formation of HEM1 condensate represses the translation efficiency of pro‐death immune genes.^[^ ^21^ ^]^ We demonstrate that the SR30 enhances tomato disease susceptibility by suppressing the AS of defense‐related genes in a phase separation manner, revealing a novel mechanism by which phase separation proteins modulate plant immunity. Similarly, condensed TaSR45a leads to dynamic AS change of plant immune genes,^[^ ^23^ ^]^ suggesting the conserve role of SR protein in AS regulation of plant immunity. Furthermore, not all SR proteins in plant species can form nuclear condensates when overexpressed. For example, TaSR45a exhibits a non‐condensate state under normal conditions or when interacting with TaHRC‐R but transforms into a condensed state upon induction by DON or when interacting with TaHRC‐S.^[^ ^23^ ^]^ Another example is that the overexpressed MtSR30 fails to form nuclear condensates.^[^ ^16^ ^]^ Thus, we speculate that the condensed states of different SR proteins need different ways to be activated. Furthermore, there is a need to further elucidate how SR30 transforms the phase separation state and affects its function.

In plants, recent studies have reported that biomolecular condensate by IDR‐containing protein is involved in plant immunity.^[^ ^23^, ^43^, ^55^ ^]^ In this study, the phase separation of SR30 is driven by both IDR2 and IDR5. The deletion of either IDR2 or IDR5 has no effect on the formation of nuclear condensates; however, the size of these condensates is altered (Figure 4; Figure S11, Supporting Information). The phase separation capability of SR30 is completely abolished only upon simultaneous deletion of both IDR2 and IDR5, while this functionality can be rescued by the incorporation of FUS's LCD domain (Figure 4). These results highlight the critical role of IDR in biomolecule condensate formation. In addition, the deletion of both IDR2 and IDR5 disrupts a large part of SR30. While the incorporation of FUS's LCD domain partially compensates for the functional deficiency caused by the absence of both IDR2 and IDR5 through condensate formation, we cannot rule out the possibility of additional mechanisms independent of phase separation. Moreover, the function of condensate is involved in its size. For example, the cellular function of EMB1579 is dependent on the formation of normal‐sized EMB1579 condensates.^[^ ^56^ ^]^ Thus, we speculate that the reason of SR30^ΔIDR2&5^‐FUS only partially rescues the function of SR30 is due to the difference in the condensate size.

There are many potential phase‐separated proteins in multiple crops, such as more than 500 potential phase‐separated proteins in tomatoes.^[^ ^57^ ^]^ However, more efforts are needed to understand their function and how they can be used to modify crop traits. A recent study shows that inactivation of the phase‐separated m^6^A reader protein SlYTH2 improves the aroma of tomato fruit for consumers' preference.^[^ ^58^ ^]^ Disrupting susceptibility (S) genes is a promising strategy for achieving broad‐spectrum and durable disease resistance in crops.^[^ ^59^ ^]^ Take an example, the knockout of *SlDMR6* confers tomato broad‐spectrum disease resistance against multiple pathogens.^[^ ^60^ ^]^ In this study, the loss of function of *SR30* enhances the disease resistance of tomatoes against multiple oomycetes pathogens (Figure 6). This finding provides an example of using phase‐separated protein and AS regulation in the modification of crop disease resistance. In addition, the specific restriction of *sr30* mutant resistance to oomycetes remains undetermined. The observed loss of disease resistance in *sr30* may be associated with either pathogen‐specific nutritional strategies or tissue‐specific expression patterns of SR30. Further investigation is warranted to elucidate the molecular mechanisms underlying the differential resistance of *sr30* against various pathogens.

## 4 Conclusion

In summary, our findings uncover a novel mechanism in which SR30, through its phase‐separation‐driven activity, negatively regulates plant immunity by modulating the alternative splicing of defense‐related genes. Remarkably, disrupting SR30's function significantly enhances tomato resistance to multiple oomycete pathogens. This study underscores the innovative role of biomolecular condensates in orchestrating plant immune responses, offering fresh insights into the interplay between phase separation and host defense mechanisms.

## 5 Experimental Section

### Plasmid Construction

For protein expression experiments in *N. benthamiana*, all SR genes, three immunity‐related genes, and two AS genes were amplified from the cDNA of *S. lycopersicum* (Heinz 1706) using Phanta Max Super Fidelity DNA Polymerase (Cat #P525, Vazyme Biotech) and subsequently ligated into the pBinGFP2 vector with the ClonExpress Ultra One Step Cloning Kit (Cat #C115, Vazyme Biotech). For phase separation assay in vitro, the GFP, GFP‐SR30, and mutant GFP‐SR30^ΔIDR2&5^ were inserted into the pMAL‐c2X vector, containing an N‐terminal His‐MBP tag, respectively. The native promoter of *SR30* and the GFP‐SR30 were fused into the pICSL86900 vector. For the silence experiments of *SR30* in tomatoes (Heinz 1706), the target region fragments of *SR30* were ligated into TRV2 and subsequently used for infiltration. All primers used for cloning and experiments are listed in Table S4 (Supporting Information). The CDS sequence the LCD of the FUS used in this study is listed in Table S5 (Supporting Information).

### Plant Material and Microbial Strain Cultivation

*N. benthamiana* plants (wild type and histone H2B‐red fluorescent protein (H2B‐RFP)) were grown in a greenhouse for 4–6 weeks under 16 h at 22 °C and 8 h at 20 °C. *Solanum lycopersicum* plants (Micro‐Tom and Heinz 1706) were grown in the greenhouse for 8–12 weeks under 14 h at 24 °C and 10 h at 22 °C. The *P. infestans* (JH19) was grown on the rye sucrose agar‐V8 medium at 18 °C. The *P. capsici* (PC35) and *P. parasitica* were grown on the V8 medium at 25 °C. The *B. cinerea* was grown on potato *D*‐glucose agar (PDA) medium at 25 °C.

### Generation of Transgenic Tomato Plants and Mutants

The pBinGFP2‐SR30 was used for *SR30* overexpression in tomatoes (Micro‐Tom). The *sr30* mutants were generated using CRISPR/Cas9‐based gene editing by the Biogle GeneTech Co. (Hangzhou, China). Two small guide RNAs were designed within *SR30* to minimize the off‐target effect. Both two designed sgRNA sequences were synthesized and cloned into the CRISPR/Cas binary vector BGK012. Two constructs were transformed into *Agrobacterium tumefaciens* (*A. tumefaciens*) strain GV3101 and introduced into the tomato cultivar Micro‐Tom via an *A. tumefaciens‐*mediated infection method.^[^ ^61^ ^]^

### *N. benthamiana* Infection Assay

For *N. benthamiana* infection, all pBinGFP2 vectors were transformed into *A. tumefaciens* strain GV3101 and used for transient expression. Cells were cultured in LB liquid medium and incubated overnight at 30 °C, then resuspended using infiltration buffer (10 mm MgCl_2_, 10 mm MES [pH = 5.6], and 150 µM acetosyringone) at a final concentration of OD_600 _= 0.1 and infiltrated into *N. benthamiana* leaves. The leaves were detached 24 h post‐agroinfiltration and inoculated with 10 µL of the *P. infestans* JH19 zoospores suspension (≈100 zoospores per microliter). The inoculated leaves were incubated in a growth chamber at 18 °C in the dark, and the lesion areas (cm^2^) were measured using ImageJ under UV light at 4–6 days after inoculation.

### Tomato Infection Assay

For gene silencing assays in tomatoes (Heinz 1706), the two‐leaf stage tomato plants were infiltrated with *A. tumefaciens* cells containing a mixture of TRV1 and TRV2 constructs or the GFP control at OD_600 _= 0.5 each. Then detached leaves were collected for pathogen infection 30–40 days following infiltration. These leaves were then inoculated with 10 µL of *P. infestans* zoospores suspension, which contained ≈50 zoospores per microliter. The tomato leaves inoculated with *P. infestans* were incubated in a growth chamber at 18 °C. The infected tomato leaves were used directly to measure lesion areas at 4 dpi. For transgenic tomato (Micro‐Tom) lines, the 8–12 weeks‐old tomato leaves were collected for *Phytophthora* pathogens infection assay. For *P. infestans*, the infected tomato leaves were stained with trypan blue at 4 dpi and then used to measure lesion area. For *P. capsici* and *P. parasitica*, the mycelium discs of 3‐day‐old were used for tomato leaves inoculation assay. The infected tomato leaves were stained with trypan blue at 2 dpi and used to measure the lesion area. For *B. cinerea*, 5 µL conidia suspension (1 × 10^8^ L^−1^) was used to inoculate tomato leaves. The disease leaves were taken photos under UV light and lesion areas were measured at 3 dpi. The *M. incognita* infected assay of tomato root was performed as previously described methods.^[^ ^62^ ^]^ All the data were analyzed by Student's *t*‐test.

### ROS Burst Assay

Tomato leaf discs (*Ø* = 0.5 cm) were cut from 8 to 12‐weeks‐old leaves and incubated for 8 h in 100 µL sterile water in a 96‐well plate without light. Leaf discs were treated with the reaction solution containing 0.5 g L^−1^ luminol, 100 nm flg22, and 0.1 g L^−1^ horseradish peroxidase. Luminescence was measured using the GloMax Discover system with Glomax Discover software (GM3000, Promega, USA). GraphPad Prism software was used for data treatment.

### RNA‐Seq Sample Collection and Data Analysis

Eight‐week‐old tomato leaves were collected and used for RNA sequencing. Total RNA was extracted using the Invitrogen PureLink RNA Mini Kit (Cat #12183018A, Invitrogen) and then used to generate RNA‐seq libraries following the manufacturer's recommendations. These libraries were sequenced using Illumina Nova in paired‐end mode with a read length of 150 bp (Berry Genomics, Beijing, China). The RNA‐seq reads with low quality were removed using trimmomatic (v0.39). Then, the remaining reads were aligned to the ITAG4.0 tomato genome (https://solgenomics.net/) using Hisat2 (v2.2.1) with default parameters. Reads mapping to multiple locations were discarded and only uniquely mapped reads were retained for subsequent analysis.

The expression levels for gene models from ITAG4.0 were measured and normalized as transcripts per million mapped reads (TPM) by stringtie (v2.2.1). DEGs were identified using the R package DESeq2 (v1.30.1). Genes with |log_2_(Foldchange)| ≥ 1 and adjusted *p*‐values (*p*‐adj) ≤ 0.05 were considered to be the threshold for identification of the DEGs. The aligned reads from each library were assembled by Cufflinks with the following option: ‐u ‐F 0.05 ‐A 0.01 ‐I 100 000 –min‐intron‐length 30.^[^ ^63^ ^]^ Then, comprehensive and unified isoform annotated files in gtf format were generated by the cuffmerge utility within the Cufflinks package to merge the above Cufflinks assemblies. Finally, different AS events were identified using rMATS (v4.1.2) with the following option: ‐t paired ‐len 150 ‐an 8 ‐c 0.0001‐analysis U ‐keep temp.^[^ ^64^ ^]^ Events with a false discovery rate < 0.05 were considered significantly differential AS events.

### Validation of AS Events and Measurement of Splicing Ratio

Total RNA was extracted using the FastPure Universal Plant Total RNA Isolation Kit (Cat #RC411, Vazyme Biotech) and quantified using a NanoDrop spectrophotometer. Approximately 1 µg total RNA was used to synthesize single‐strand cDNA using the HiScript III RT SuperMix for qPCR (Cat #R323‐01, Vazyme Biotech). The qRT‐PCR was performed using the Vazyme ChamQ Universal SYBR qPCR Master Mix (Cat #Q711‐02, Vazyme Biotech). Validation of the AS event and measurement of the splicing ratio were performed as previously reported.^[^ ^30^ ^]^ AS events were validated by determining the transcript levels of different splice isoforms using isoform‐specific primers. The primers for spliced isoforms were designed to cross the exon–exon junction, and the primers for unspliced isoforms were designed to span the intron–exon junction. These isoform‐specific primers are listed in Table S4 (Supporting Information).

### Confocal Microscopy and FRAP Assay

For gene overexpression, *A. tumefaciens* cultures were resuspended in an infiltration buffer at a final concentration of OD_600 _= 0.5 and used for infiltration of *N. benthamiana* leaves (WT and H2B‐RFP). The partial *N. benthamiana* leaves expressing the target proteins were mounted in distilled water and observed using a laser scanning confocal microscope (Carl Zeiss LSM 980 with Airyscan2 or LSM 710, Germany). For 1,6‐hexanediol (Cat #240117, Sigma‐Aldrich) treatment, *N. benthamiana* leaves were infiltrated with 5% 1,6‐hexanediol before imaging. Green and red fluorescence were observed at excitation of 488 and 561 nm, respectively.

FRAP of GFP‐SR30 condensates in epidermal cells of *N. benthamiana* leaves was performed on a Zeiss LSM microscope (Carl Zeiss LSM 980 with Airyscan2) using a 63× water immersion objective. Condensates were bleached using a laser intensity of 50% at 488 nm with 50 iterations. Images and analysis of the fluorescence intensity of the bleached region, reference region, and background region were acquired using ZEN3.3 software (Blue Edition). Fluorescence recovery curves were generated using the web‐based tool easyFRAP (http://easyfrap.vmnet.upatras.gr).^[^ ^65^ ^]^

### Phase Separation Assay In Vitro

His‐MBP‐GFP, His‐MBP‐GFP‐SR30, and His‐MBP‐GFP‐SR30^ΔIDR2&5^ plasmids were transformed into *E. coli* strain DE3 and incubated with 0.1 mm isopropyl‐β‐D‐1‐thiogalactopyranoside for 24 h at 16 °C when the cell concentration was OD_600 _= 0.4–0.6. Cells were harvested and resuspended in lysis buffer (50 mm Tris‐HCl pH = 7.5, 300 mm NaCl, 10 mm imidazole, 1 mm phenylmethanesulfonyl fluoride). The recombinant proteins were purified using the HisSep Ni‐NTA 6FF chromatography column (Cat #20504ES08, Yeasen Biotech). Recombinant Tobacco Etch Virus (rTEV) protease (Cat #C500302, Sangon Biotech) was then added to remove the purification tags. His‐tagged rTEV protease and purification tags were removed over the Ni‐NTA column while the cleaved protein was concentrated in 20 mm Tris‐HCl (pH 7.5), 50 mm NaCl, and 2 mm dithiothreitol, and stored at −80 °C.

The 1 µm purified GFP, GFP‐SR30, and GFP‐SR30^ΔIDR2&5^ were each added into a fluted slide with a coverslip at 37 °C for 1 h and then directly visualized under the laser scanning confocal microscope (Carl Zeiss LSM 710 or LSM 980 with Airyscan2, Germany). The green fluorescence was observed at 488 nm excitation.

### Western Blotting Assay

Proteins were separated by 10% SDS‐PAGE gel. The western blotting steps were performed as previously described.^[^ ^15^ ^]^ Proteins were detected using anti‐GFP antibody (1: 5000, Cat #M20004, Abmart) and IRDye goat anti‐mouse 800CW antibody (1:10 000, Cat #D20802‐25, Li‐Cor).

### Split‐LUC Assay

SR30, SR30^ΔIDR2&5^, and SR30^ΔIDR2&5^‐FUS were individually ligated into the pICH86988‐nLUC (nLUC) vector, while other tomato splicing factors were ligated into the pCAMBIA1300‐cLUC (cLUC) construct. SR30‐nLUC, SR30^ΔIDR2&5^‐nLUC, and SR30^ΔIDR2&5^‐FUS‐nLUC were co‐expressed in *N. benthamiana* leaves with cLUC each containing different splicing factors. The infiltrated concentration is OD_600 _= 0.5 for each co‐expressed protein. *N. benthamiana* leaves were collected 48 h after infiltration and incubated with 1 mm *D*‐luciferin potassium salt substrate solution (Cat #40902ES03, Yeasen Biotech). The LUC images were captured using a Tanon‐5200 Multi Chemiluminescent Imaging System (Tanon, China). To quantify the LUC signal, leaf discs of *N. benthamiana* were collected 48 h after infiltration and incubated with 1 mm *D*‐luciferin substrate solution in a 96‐well plate. Relative LUC activity was quantified using a Promega GloMax Navigator microplate luminometer (Promega, USA).

### Yeas Two‐Hybrid Assay

SR30 and its mutants were cloned into pGBKT7, and five tomato splicing factors were ligated into pGADT7 in a previous study.^[^ ^30^ ^]^ All steps in this assay were performed as previously described.^[^ ^30^ ^]^

### Bioinformatic Analysis

Domain prediction of 18 SR proteins was performed using PROSITE (https://prosite.expasy.org/). The domain prediction of UBC34 and CCR4E was conducted using the SMART tool (http://smart.embl‐heidelberg.de/), and the domain of ARP8 was predicted through the conserved domain database (https://www.ncbi.nlm.nih.gov/ Structure/cdd/wrpsb.cgi). Prediction of IDRs was carried out using the PONDR website (www.pondr.com). Venn diagrams were generated using the TBtools software (v2.096).^[^ ^66^ ^]^ The heatmap of the PSI analysis and the volcano plot analysis were performed by the tool website (https://www.bioinformatics.com.cn/).

### Statistical Analysis

All assays were conducted with at least three replicates per line. GraphPad Prism 8 was used to calculate the means (±SE). Statistical significance was determined using the Student′s *t‐*test by Microsoft Excel 2019. Significance levels were defined as follows: *, *P <* 0.05; **, *P < *0.01.

## Conflict of Interest

The authors declare no conflict of interest.

## Author Contributions

S.D., J.H., and D.Y. conceived and designed the experiments. D.Y., J.H., F.T., H.W., and Q.P. performed the experiments. H.S. and H.C. analyzed the RNA‐seq data. F.T. performed transgenic tomatoes. J.Z. performed the root‐knot nematode infection experiments of tomatoes. Y.W., G.L., and H.C. provided suggestions for experiments. D.Y. and J.H. wrote the manuscript. S.D., A.S.N.R., and G.L. modified the manuscript. All authors commented on the article before submission.

## Supporting information



Supporting Information

Supplemental Movie 1

Supplemental Movie 2

Supplemental Dataset 1

Supplemental Dataset 2

Supplemental File S1

Supplemental File S2

Supplemental File S3

## Data Availability

The data that support the findings of this study are available from the corresponding author upon reasonable request.
