# Supplementary material for: A Phase‐Separated SR Protein Reprograms Host Pre‐mRNA Splicing to Enhance Disease Susceptibility
Source: Adv Sci (Weinh). 2025 May 8;12(27):2500072. doi: 10.1002/advs.202500072 (PMC12279203; doi:10.1002/advs.202500072)
Supplement: Supplementary file 1 — Supporting Information [file ADVS-12-2500072-s001.docx]

Supporting Information

**A Phase-Separated SR Protein Reprograms Host Pre-mRNA**

**Splicing to Enhance Disease Susceptibility**

*Dong Yan*, *Jie Huang**, *Fengqi Tian*, *Haidong Shu*, *Han Chen*, *Qian Peng*, *Hongwei Wu*, *Jianlong Zhao*, *Anireddy S. N. Reddy*, *Gang Li*, *Yuanchao Wang*, *Suomeng Dong**

* E-mail: [smdong@njau.edu.cn](mailto:smdong@njau.edu.cn), [jie.huang@biology.ox.ac.uk](mailto:jie.huang@biology.ox.ac.uk)

**This supporting information contains:**

| **Items** | **Content** | **Page** |
| --- | --- | --- |
| Figure S1 | Screening of SR family proteins involved in plant immunity | 3 |
| Figure S2 | The overexpression of *SR30* promotes *P. infestans* infection | 5 |
| Figure S3 | The overexpression of *SR30* led to alternative splicing change in tomato | 6 |
| Figure S4 | Analysis of differentially expressed genes between *SR30*-OE versus WT | 7 |
| Figure S5 | Experimental validation of AS events by qRT-PCR | 8 |
| Figure S6 | The domain schematic diagrams of predicted proteins produced by different transcript isoforms | 10 |
| Figure S7 | Function analysis of different transcript isoforms of three defense-related genes | 11 |
| Figure S8 | Co-localization of SR30 protein with other splicing proteins in *N. benthamiana* | 12 |
| Figure S9 | Detection of purified GFP, GFP-SR30, and GFP-SR30^ΔIDR2&5^ protein *in vitro* | 13 |
| Figure S10 | IDR2 and IDR5 are located in the flexibility region of SR30 | 14 |
| Figure S11 | The effect of IDR on the phase separation of SR30 | 15 |
| Figure S12 | The *P. infestans* infection assay of phase separation mutants of SR30 | 16 |
| Figure S13 | Relative expression level of different transcript isoforms of three DASGs under different treatments | 17 |
| Figure S14 | The associations between SR30 mutants and other splicing factors via split-LUC assays and yeast two-hybrid assays | 18 |
| Figure S15 | The growth phenotype of tomato *sr30* mutant | 20 |
| Figure S16 | The knockout of *SR30* did not improve tomato resistance against *Botrytis* *cinerea* and *Meloidogyne incognita*. | 21 |
| Figure S17 | The relative expression level of different transcript isoforms of three DASGs in *sr30* mutant under *P. infestans* infection. | 22 |
| Table S1 | Information of the SR family proteins identified in *Solanum lycopersicum* | 24 |
| Table S2 | Information of RNA-seq data | 25 |
| Table S3 | Protein sequences of different transcript isoforms of three DASGs | 26 |
| Table S4 | Primer sequences used in this study. | 27 |
| Table S5 | The CDS sequence of the LCD of the FUS used in this study | 30 |
| References |  | 31 |
| The legends for supplementary files |  | 32 |


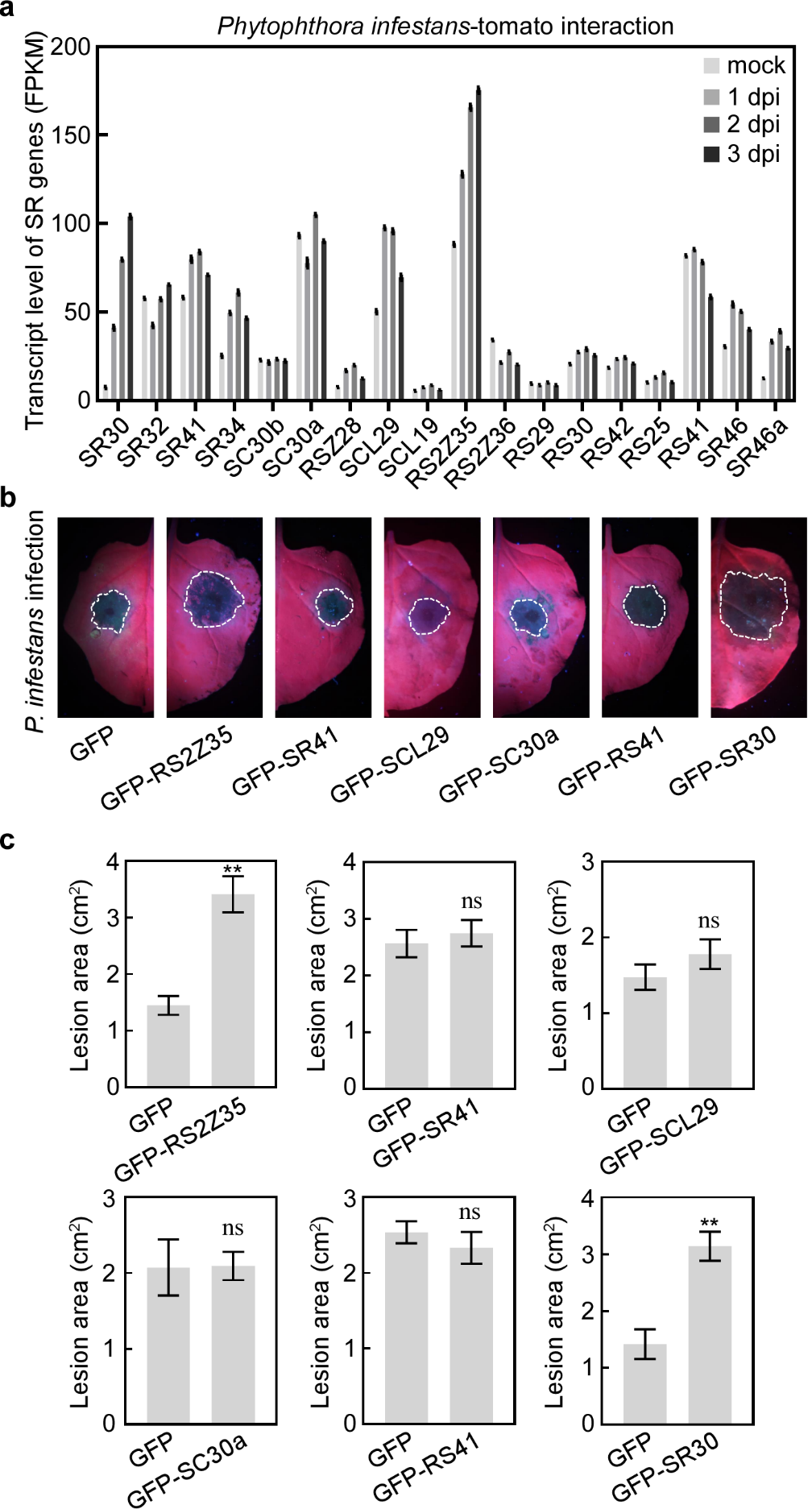


**Figure S1.** Screening of SR family proteins involved in plant immunity.

a) Transcript levels of 18 SR protein genes based on RNA-seq analysis of tomato response to the *Phytophthora infestans* infection^[1]^. The vertical coordinate represents represent the FPKM (Fragments Per Kilobase of transcript per Million mapped reads) value. b) Infection assays on the *Nicotiana benthamiana* leaves expressing SR proteins. *Phytophthora infestans* JH19 zoospores were inoculated on the infiltrated leaves one day post-agroinfiltration. Photographs were taken 5 days post-inoculation (dpi) under the UV light. The dashed lines indicate the lesion areas. The GFP was used as a control. c) Lesion area of *N. benthamiana* leaves expressing SR proteins infected by *P. infestans*. Data represents the mean with standard errors (SE) (n=12). *P* values were analyzed by Student's *t*-test (**, *P*<0.01; ns: no significant difference, *P>*0.05).


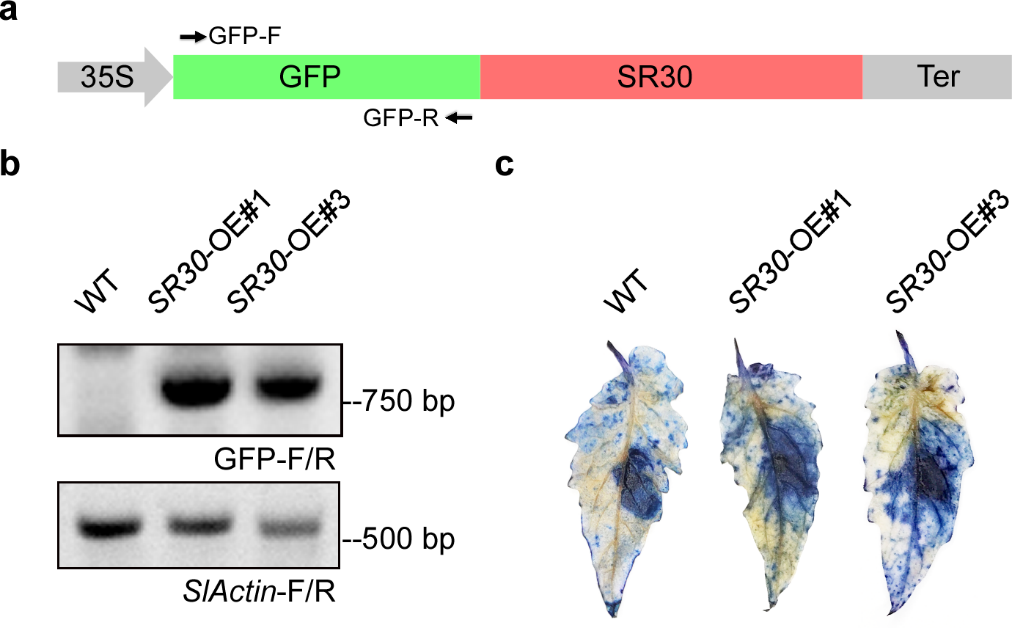


**Figure S2.** The overexpression of *SR30* promotes *P. infestans* infection.

a) Schematic diagram of the *35S::GFP-SR30* construct used to generate transgenic tomatoes. Arrows represent the position of specific primers used for PCR detection of the introduced gene in transgenic tomatoes in (b). b) PCR detection of GFP in wild-type (WT) and two *SR30*-OE transgenic tomato (Micro-Tom) lines using specific primers in (a). Tomato gene *SlActin* was used as an internal control. c) The image showed that the overexpression of *SR30* promoted the growth of *P. infestans* lesion. Detached leaves of *SR30*-OE and WT were inoculated with *P. infestans* JH19 zoospores. Infected leaves were stained with trypan blue and photographed at 4 dpi.


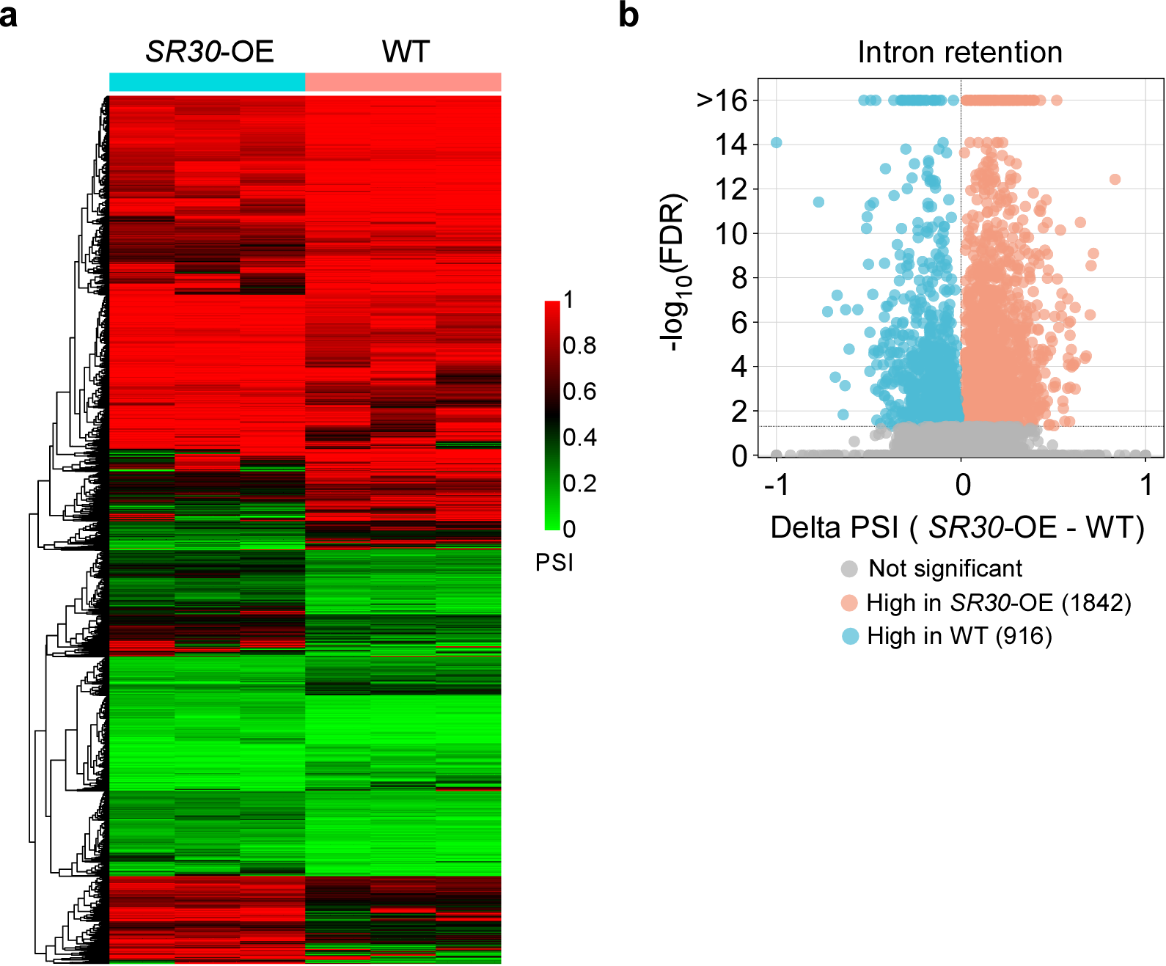


**Figure S3.** The overexpression of *SR30* led to alternative splicing change in tomato.

a) Heatmap of percent spliced in (PSI) values for differentially alternative splicing (DAS) events comparing *SR30*-OE versus WT. DAS events with |ΔPSI| ≥0.05 are shown. PSI= splice_in / (splice_in + splice_out). The figure was generated by the tool website (<https://www.bioinformatics.com.cn/>). b) Volcano plot of intron retention events identified in the comparison of *SR30*-OE versus WT. Blue dots represent IR events with ΔPSI<0, orange dots represent IR events with ΔPSI>0, and grey dots represent the IR events that are not significantly differentially alternative spliced. The numbers in parentheses indicate the number of IR events. IR events with a false discovery rate (FDR) <0.05 were considered significantly differentially IR events.


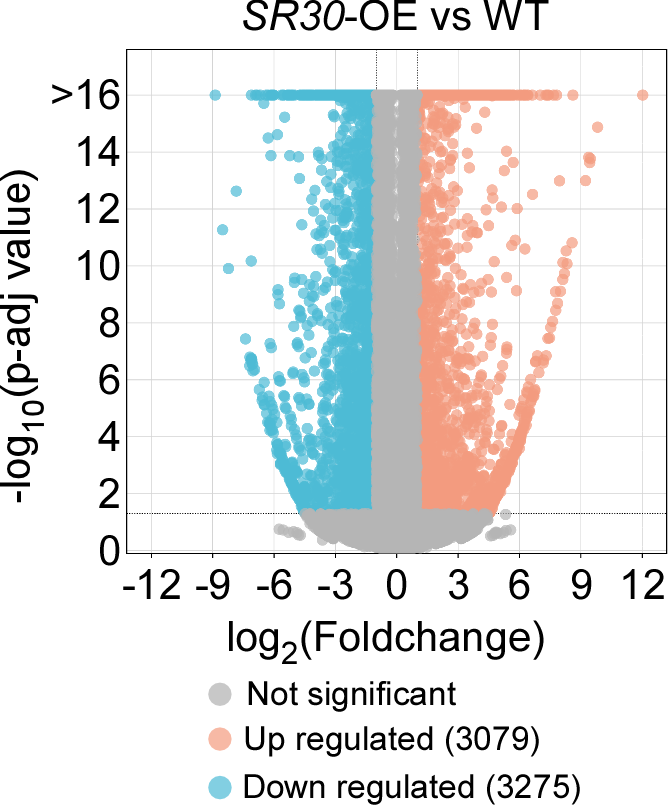


**Figure S4.** Analysis of differentially expressed genes between *SR30*-OE versus WT.

Blue dots represent downregulated genes, orange dots represent upregulated genes, and grey dots represent genes that were not differentially expressed. The numbers in parentheses indicate the number of genes. Genes with |log_2_(Foldchange)| ≥1 and adjusted *p* value (*p*-adj) <0.05 were considered as significantly differentially expressed genes.


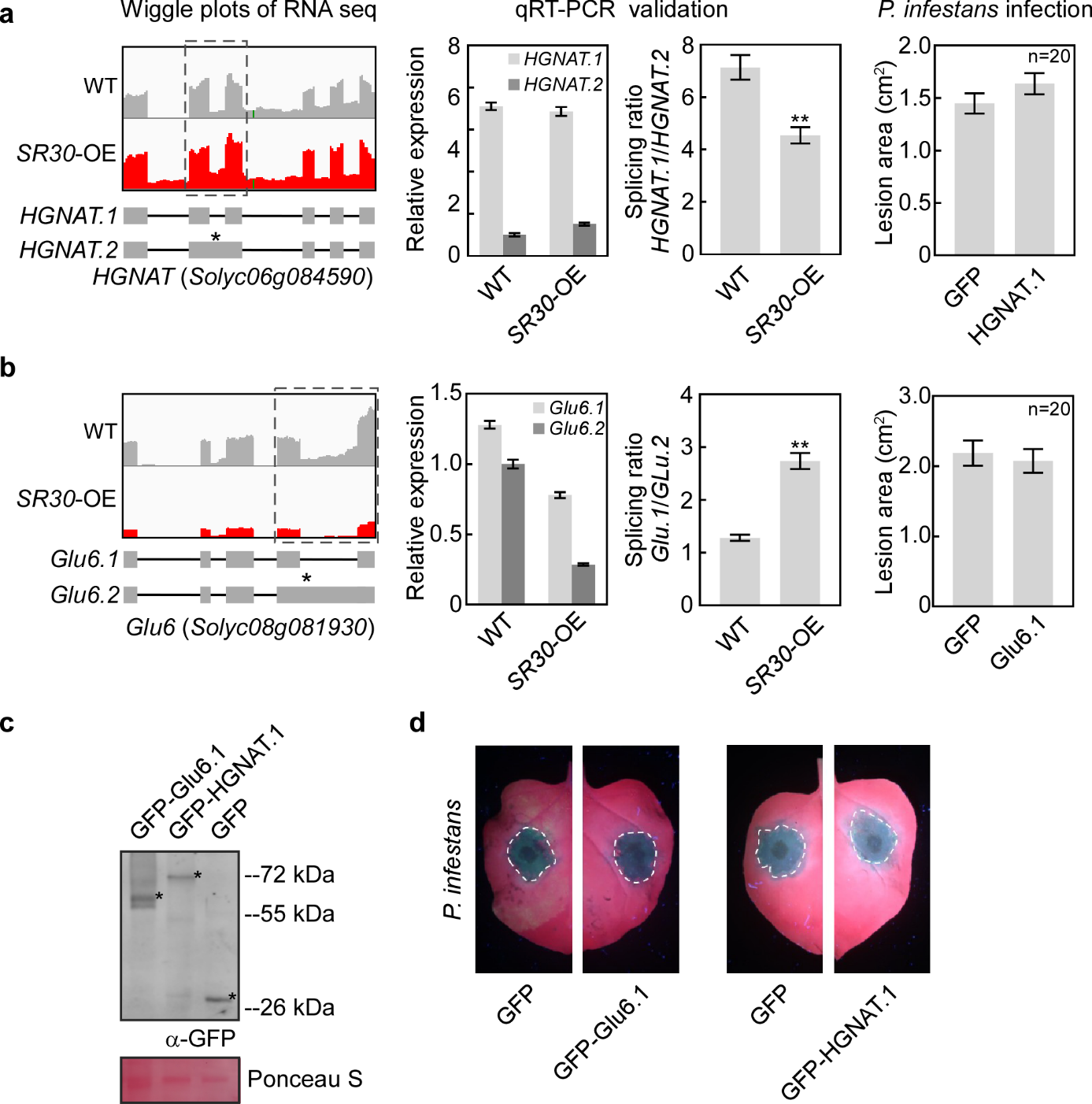


**Figure S5.** Experimental validation of AS events by qRT-PCR.

a-b**)** The qRT-PCR assays were performed to validate the ratio of different transcript isoforms of *HGNAT* (*hepara-alpha-glucosaminide N-acetyltransferase*) (a) and *Glu6* (*glucose-6-phosphate 1-epimerase*) (b). The left panel shows wiggle plots of RNA-seq data for two selected genes with schematic gene models of different isoforms. The asterisks indicate premature termination codons. The second panel from the left shows the relative transcript level of two isoforms of *HGNAT* and *Glu6*. The third panel from the left shows the splicing ratio of *HGNAT* (*HGNAT.1/HGNAT.2*) and *Glu6* (*Glu6.1/Glu6.2*). Data represent the mean with SE (n=3). *P* values were analyzed by Student's *t*-test (**, *P*<0.01). The tomato ubiquitin gene, *UBI*, was used as an internal control gene. The right panel shows the *P. infestans* lesion areas of *N. benthamiana* leaves expressing HGNAT.1 and Glu6.1. Data represent the mean with SE (n=20). *P* values were analyzed by Student's *t*-test (*P*>0.05). c) Western blot detection of GFP-Glu6.1 and GFP-HGNAT.1 using anti-GFP body. Asterisks indicate predicted protein bands corresponding to each construct. The protein loading was visualized by Ponceau S staining. d) The image showing the *N. benthamiana* leaves expressing GFP-Glu6.1 and GFP-HGNAT.1, respectively, infected by *P. infestans* were taken at 5 dpi under UV light. The dashed lines show the lesion areas.


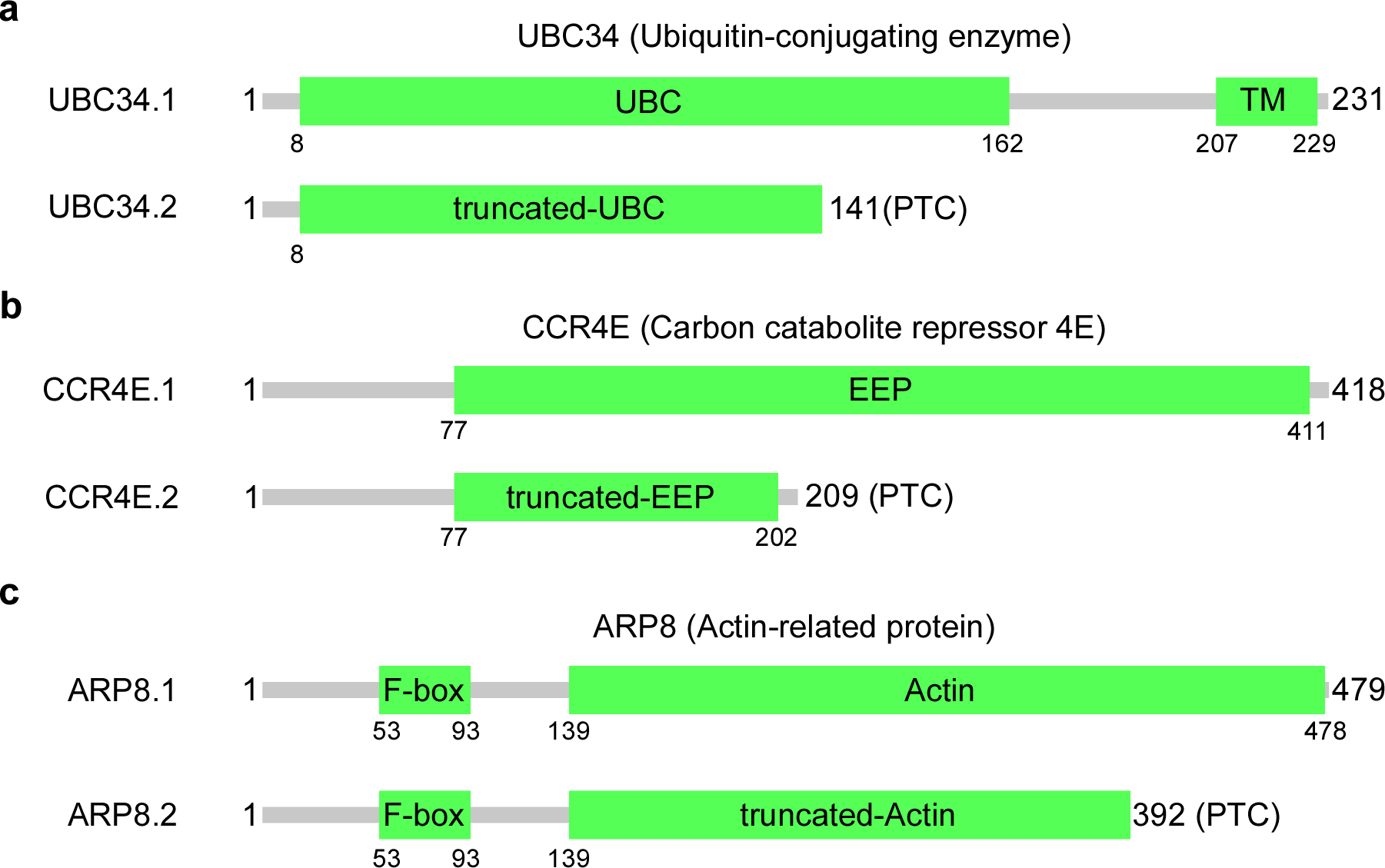


**Figure S6.** The domain schematic diagrams of predicted proteins produced by different transcript isoforms.

a) *UBC34* is predicted to encode a ubiquitin-conjugating enzyme. The *UBC34.1* transcript will produce a functional ubiquitin-conjugating enzyme, whereas *UBC34.2* produces a truncated protein that lacks a transmembrane (TM) domain and disrupts the intact UBC domain due to intron retention. PTC, premature termination codon. b) *CCR4E* is predicted to encode a carbon catabolite repressor. The *CCR4E.1* transcript will produce a functional carbon catabolite repressor containing an exonuclease-endonuclease-phosphatase (EEP) domain. However, the *CCR4E.2* transcript produces a truncated protein that disrupts the intact EEP domain due to PTC caused by intron retention. c) *ARP8* is predicted to encode an actin-related protein. The *ARP8.1* transcript will produce a functional protein containing an F-box and an actin domain, whereas the *ARP8.2* transcript undergoes intron retention, resulting in a truncated protein lacking the intact actin domain. For a-c, the numbers indicate the animo acid position of each protein.


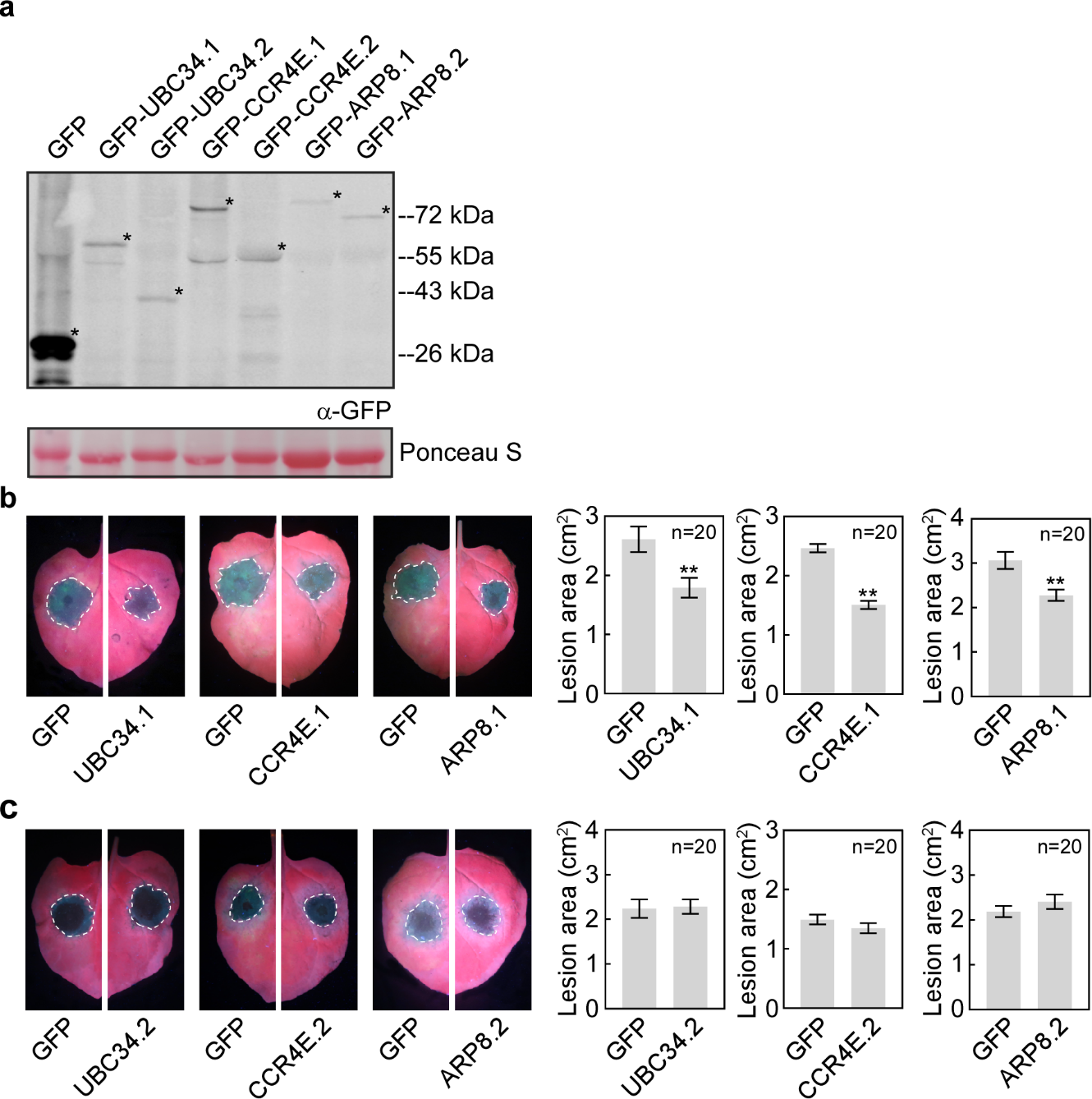


**Figure S7.** Function analysis of different transcript isoforms of three defense-related genes.

a) Western blot detection of protein produced by different transcript isoforms of three defense-related genes using the anti-GFP body. Asterisks indicate predicted protein bands corresponding to each construct. The protein loading was visualized by Ponceau S staining. b) The *P. infestans* infection assay on *N. benthamiana* leaves expressing functional isoforms of defense-related genes. The GFP vector was used as a control. The images showing the *N. benthamiana* leaves infected by *P. infestans* were taken at 5 dpi under UV light. The dashed lines indicate the lesion areas. The columns showed the lesion areas of these infection assays. Data represents the mean with SE (n=20). *P* values were analyzed by Student's *t*-test (**, *P*<0.01). c) Immunity function analysis of intron-retained isoforms that produce a truncated protein. The photos showed *N. benthamiana* leaves expressing intron-retained transcripts of three defense-related genes infected by *P. infestans*. The photographs were taken at 5 dpi under UV light. The dashed lines indicate the lesion areas. The columns displayed the lesion area measurement of these infection assays. Data represents the mean with SE (n=20). *P* values were analyzed by Student's *t*-test (*P*>0.05).


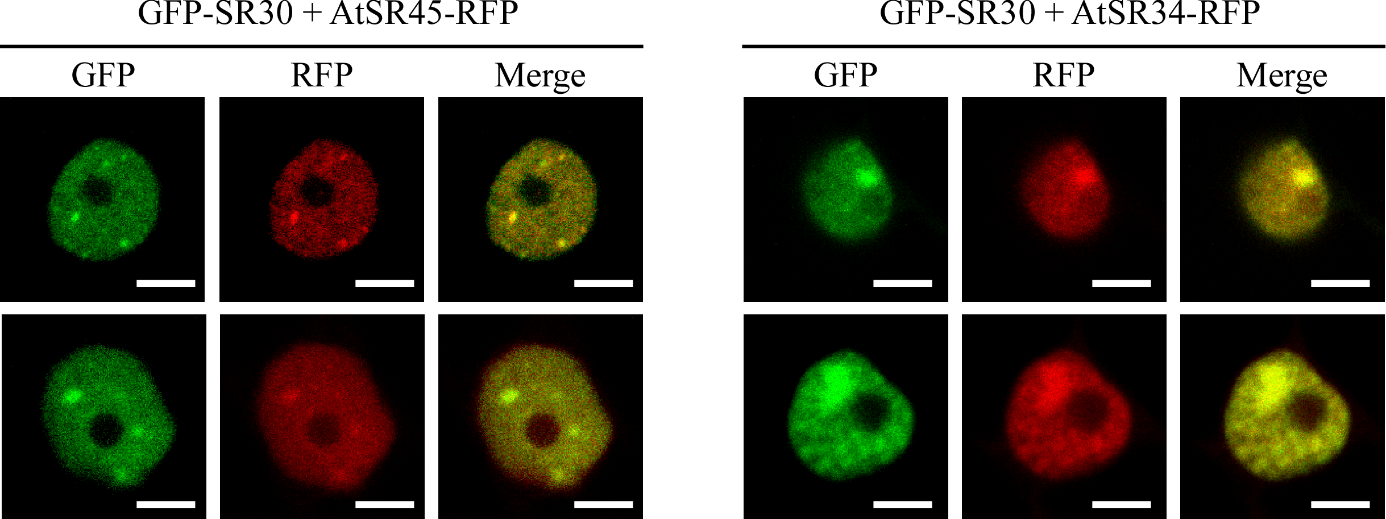


**Figure S8.** Co-localization of SR30 protein with other splicing proteins in *N. benthamiana*.

The confocal imaging shows that GFP-SR30 colocalizes with AtSR45-RFP and AtSR34-RFP in the nuclear speckles. GFP-SR30 and AtSR45-RFP or AtSR34-RFP were transiently co-expressed in a *N. benthamiana* leaves for 48 h before imaging by a confocal microscope (63× objective). The scale bar represents 5 μm. AtSR45 has been demonstrated to function as a splicing factor in an *in vitro* splicing assay ^[2]^ and it localizes to nuclear speckles with other spliceosomal proteins including U2AF^35^a and U2AF^35^b ^[3-5]^.


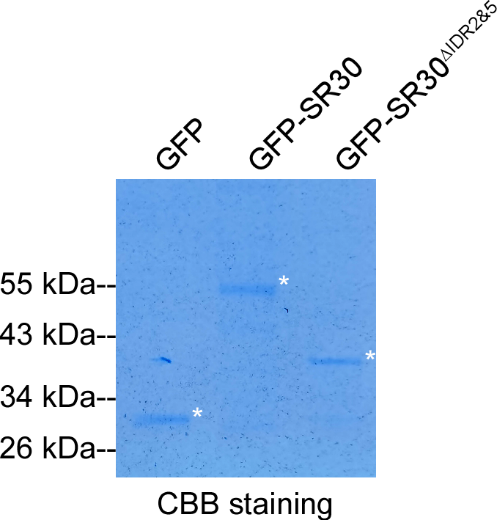


**Figure S9.** Detection of purified GFP, GFP-SR30, and GFP-SR30^ΔIDR2&5^ protein *in vitro*.

Coomassie brilliant blue (CBB) staining of the purified GFP, GFP-SR30, and GFP-SR30^ΔIDR2&5^ proteins. White asterisks indicate the size of target protein bands.


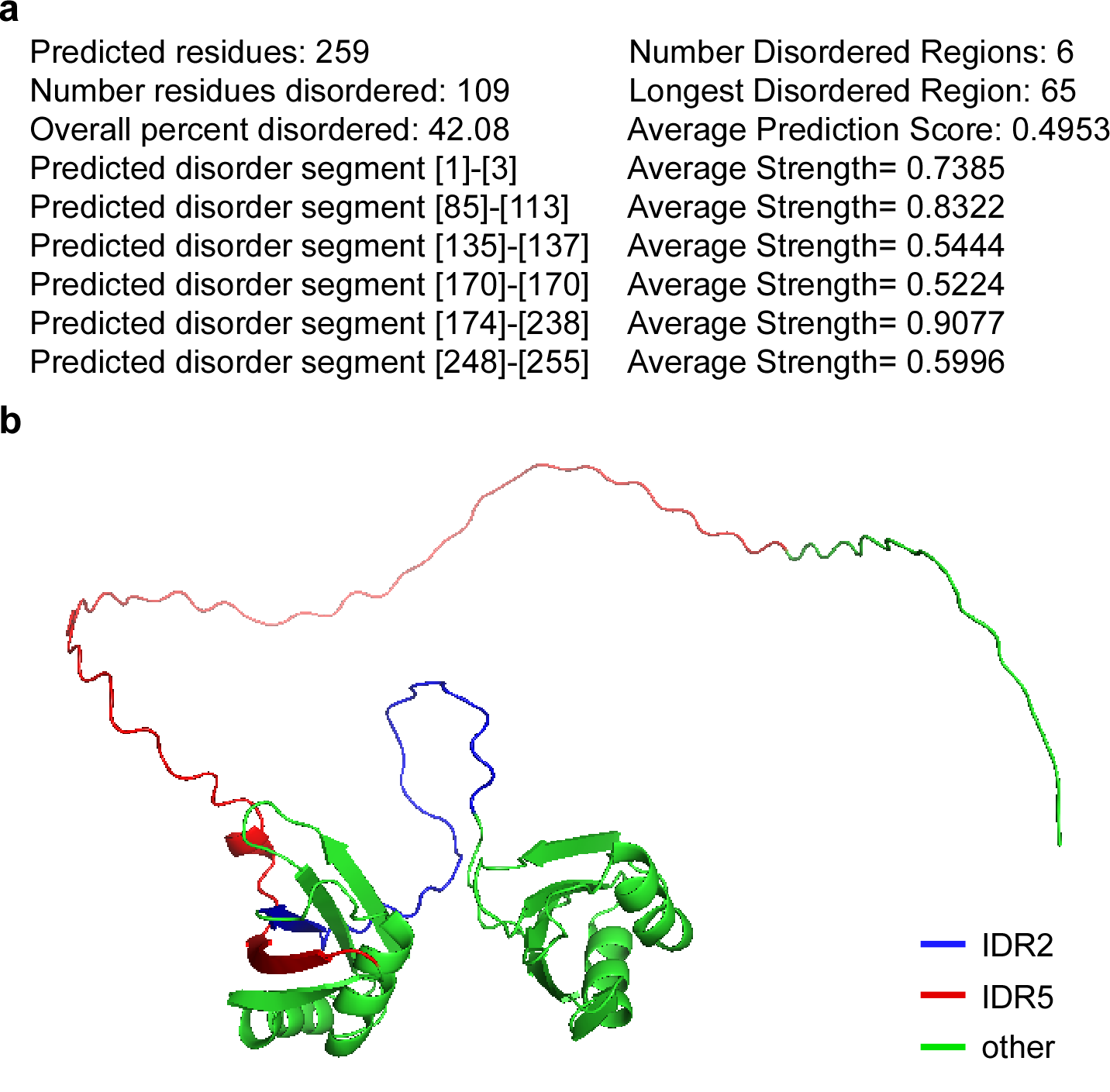


**Figure S10.** IDR2 and IDR5 are located in the flexibility region of SR30.

a) Information on the SR30′s IDRs. IDR2 and IDR5 contain more disordered amino acids, while the remaining IDRs only have a few disordered amino acids. b) The three-dimensional structure of SR30 protein predicted using AlphaFold 3. The blue and red regions represent IDR2 and IDR5, respectively, and the remaining region is shown in green. The image was generated using PyMOL2 software (V3.0.3).


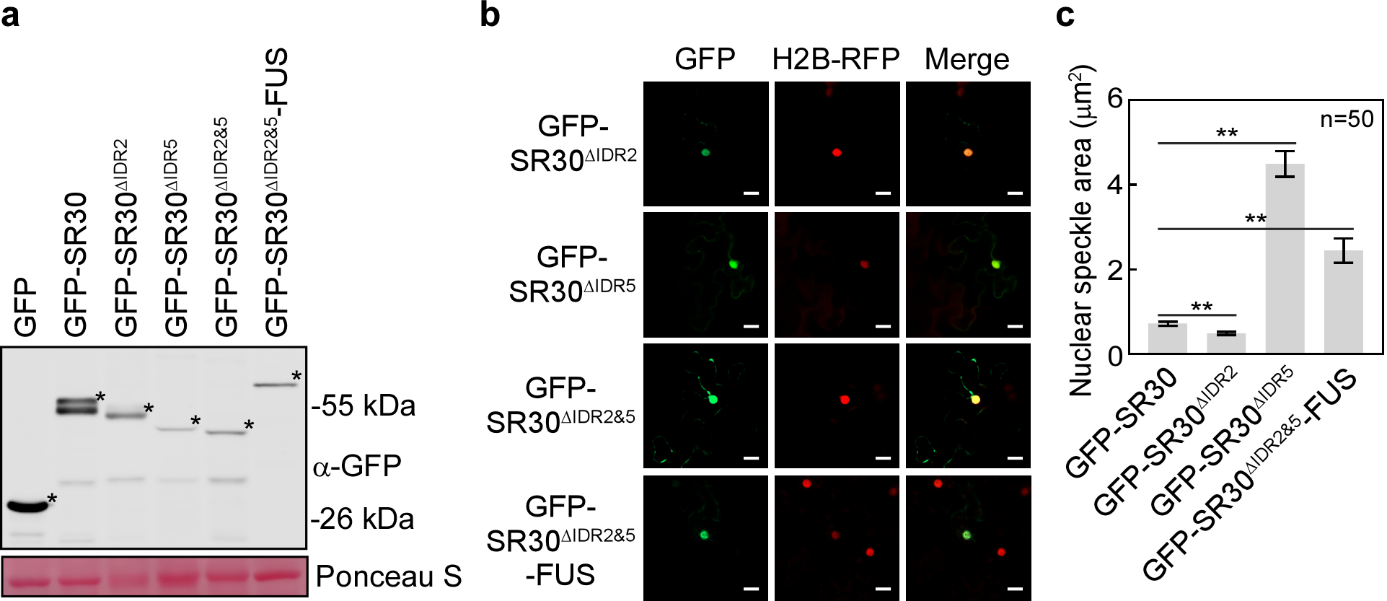


**Figure S11.** The effect of IDR on the phase separation of SR30.

a) Protein detection of GFP, GFP-SR30, IDR-deletion mutants, and GFP-SR30^ΔIDR2&5^-FUS using the anti-GFP antibody. Asterisks indicate the predicted protein bands corresponding to each construct. Protein loading was visualized by Ponceau S staining. b) Subcellular localization of IDR mutants of SR30 using a 20× objective. The observation was performed using a confocal microscope when the IDR-deletion mutants were expressed in a transgenic *N. benthamiana* leaves expressing a nuclear marker H2B-RFP for 48 h. Scale bars represent 20 μm. c) The area of nuclear condensates formed by GFP-SR30, GFP-SR30^ΔIDR2^, GFP-SR30^ΔIDR5^, and GFP-SR30^ΔIDR2&5^-FUS expressed in *N. benthamiana* leaves. The ZEN3.3 software (Blue edition) is used to delineate condensates directly, and it automatically calculates the area for each. Data represent the mean with SE (n=50). The *P* values were analyzed by Student's *t*-test (**, *P*<0.01).


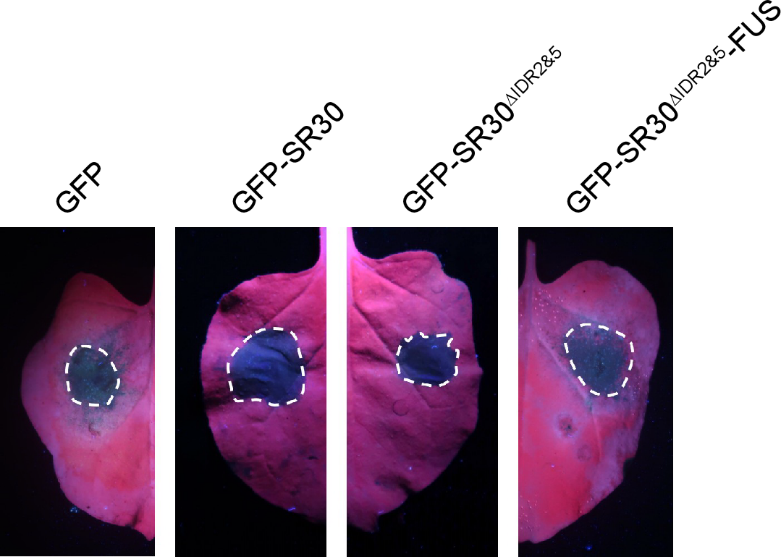


**Figure S12.** The *P. infestans* infection assay of phase separation mutants of SR30.

The image showed the *P. infestans* lesions of on *N. benthamiana* leaves expressing GFP, GFP-SR30, GFP-SR30^ΔIDR2&5^, and GFP-SR30^ΔIDR2&5^-FUS, respectively. Photographs were taken at 5 dpi under UV light. The dashed lines indicate the lesion areas.


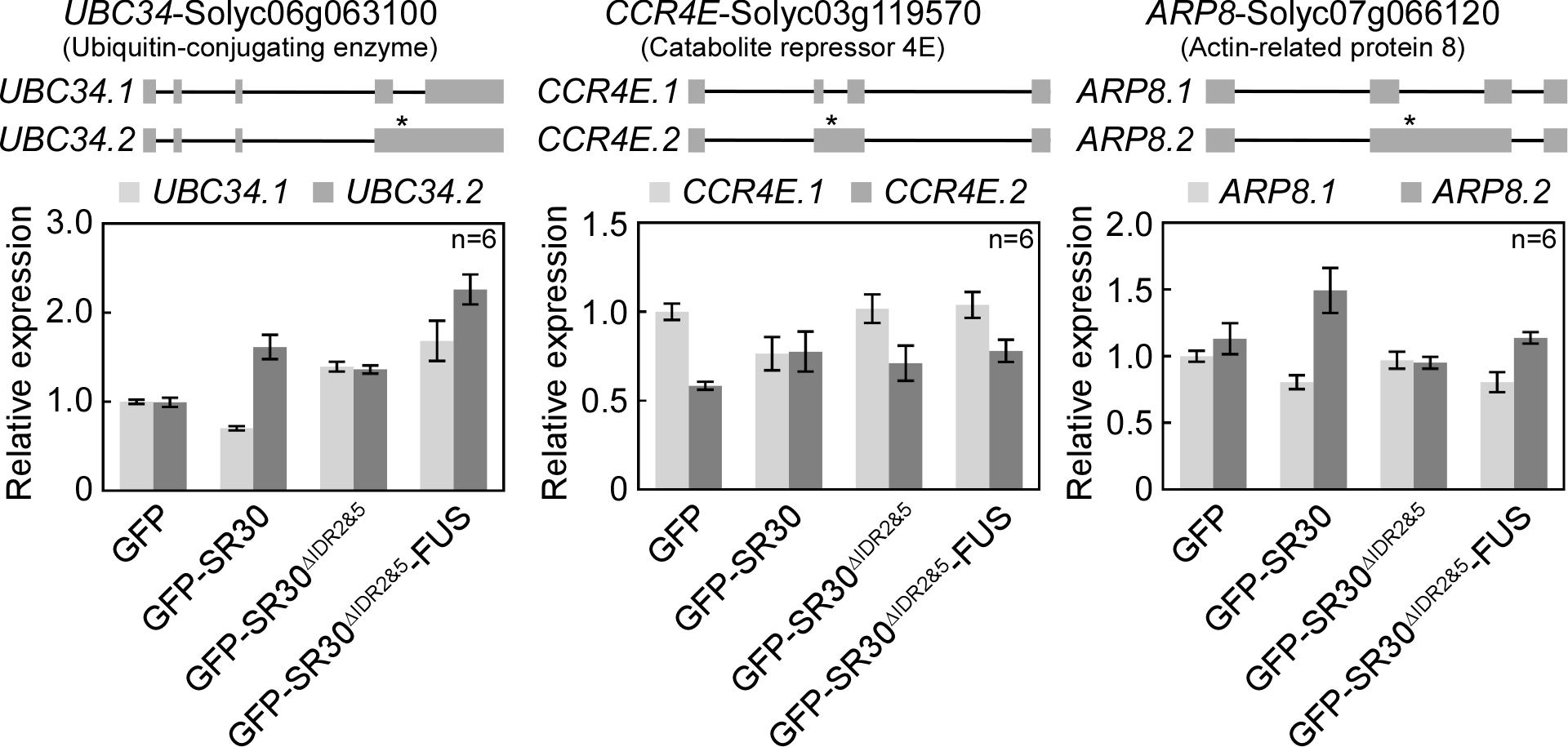


**Figure S13.** Relative expression level of different transcript isoforms of three DASGs under different treatments.

The qRT-PCR assays were performed to measure the relative expression level of different transcript isoforms of three DASGs under treatments of GFP, GFP-SR30, GFP-SR30^ΔIDR2&5^, and GFP-SR30^ΔIDR2&5^-FUS using isoform-specific primers. The relative express levels of transcript isoforms under different treatments are normalized to the transcript level of *UBC34.1*, *CCR4E.1*, or *ARP8.1* in the GFP control, respectively. The gene *NbActin* was used as an internal control. Asterisks represent the position of a premature termination codon caused by intron retention. All experiments were repeated twice with similar results.


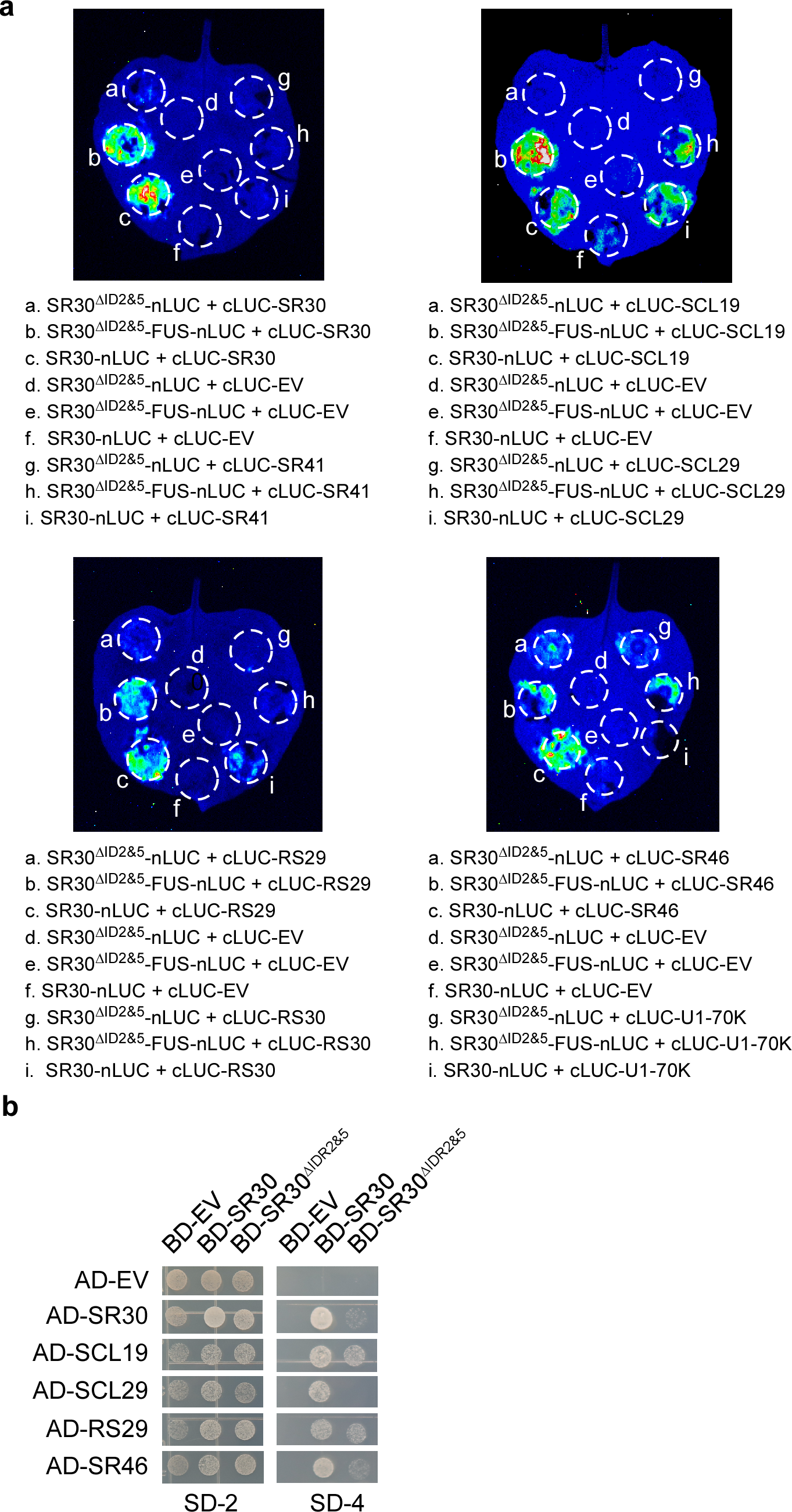


**Figure S14.** The associations between SR30 mutants and other splicing factors via split-LUC assays and yeast two-hybrid assays.

a) Split-LUC assays were performed to test the interaction of SR30, SR30^ΔIDR2&5^ and SR30^ΔIDR2&5^-FUS with other tomato splicing factors. Leaves were used to measure the LUC activity 48 h after co-expression of the indicated protein using a chemiluminescent imaging system. The LUC image was captured using the Tanon Chemiluminescent Imaging System. b) Yeast two-hybrid assays were performed to test the interaction of SR30, SR30^ΔIDR2&5^ and SR30^ΔIDR2&5^-FUS with different tomato splicing factors. The yeast cells were diluted 100-fold and then cultured on selective media to evaluate potential protein-protein interactions.


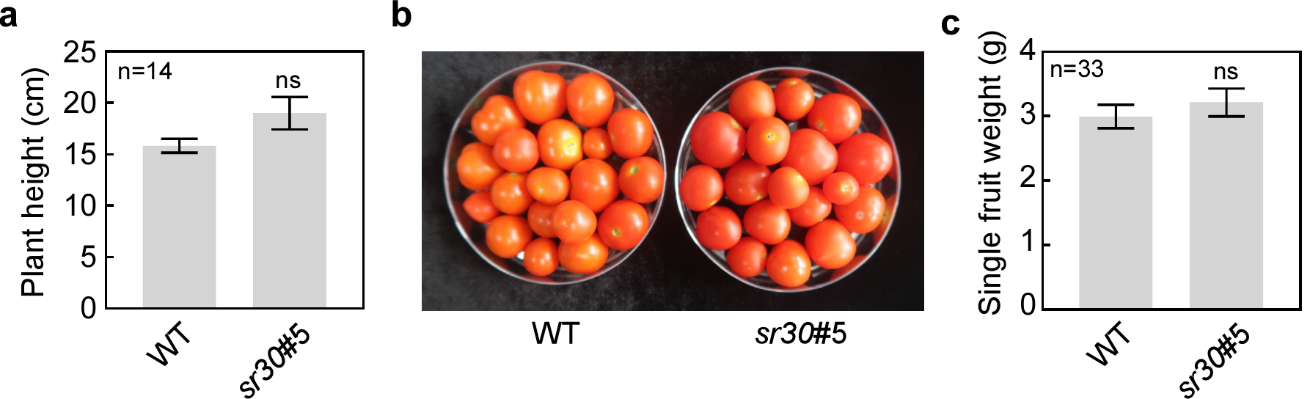


**Figure S15.** The growth phenotype of tomato *sr30* mutant.

a) The plant height of *sr30*#5 and WT tomato plants at 100 days old. Data represent the mean with SE (n=14). b) Photos showing the fruits of *sr30*#5 and WT. c) The single fruit weight of *sr30*#5 and WT plant. Data represent the mean with SE (n=33). *P* values were analyzed by the Student′s *t*-test. ns: no significant difference (*P*>0.05).


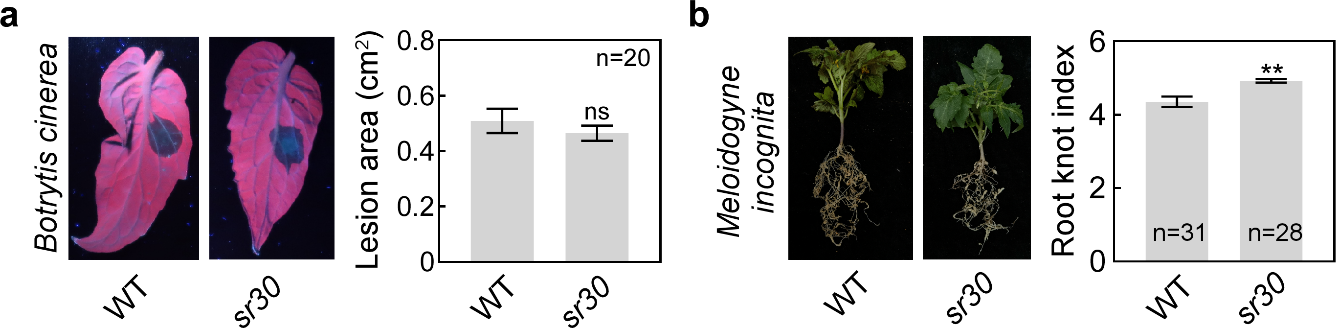


**Figure S16.** The knockout of *SR30* did not improve tomato resistance against *Botrytis* *cinerea* and *Meloidogyne incognita*.

a) The knockout of *SR30* cannot significantly suppress *B. cinerea* infection. The images showing the lesions of *B. cinerea* of WT and *sr30* tomato leaves were taken under UV light at 3 dpi. The column displayed the lesion area measured at 3 dpi. The data represents the mean with SE (n=20). *P* values were analyzed by the Student′s *t*-test (ns: no significant difference, *P*>0.05). b) The knockout of *SR30* cannot repress *M. incognita* infection for tomato roots. Both WT and *sr30* tomatoes are susceptible to *M. incognita*. The images exhibited the root-knots of WT and *sr30* tomato roots at 40 dpi with *M. incognita*. The column showed the disease index of WT and *sr30* tomato evaluated at 40 dpi. The data represents the mean with SE (n=31 for WT; n=28 for *sr30*). *P* values were analyzed by the Student′s *t*-test (**, *P*<0.01).


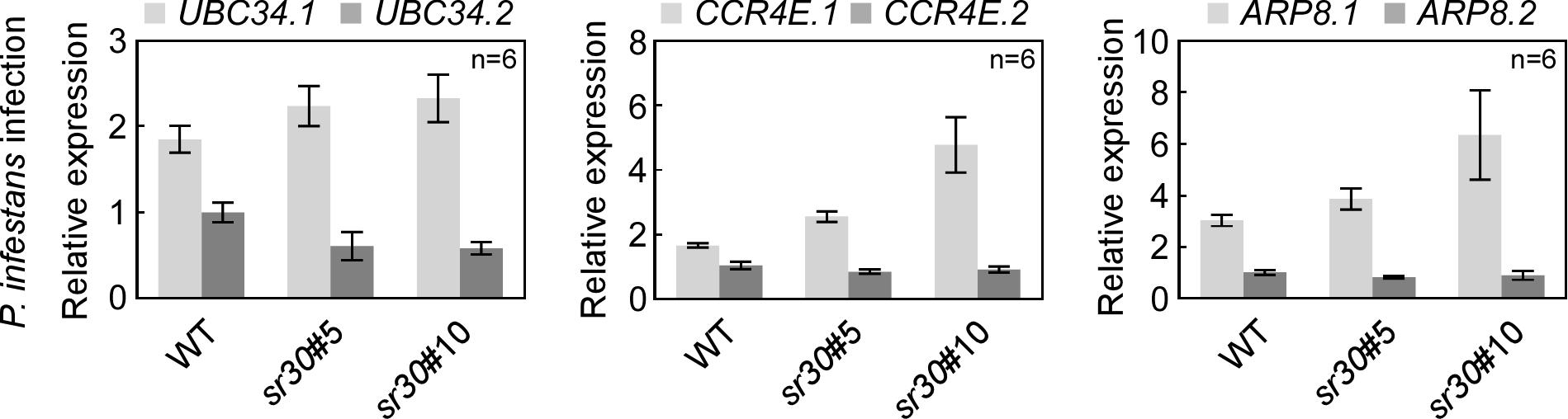


**Figure S17.** The relative expression level of different transcript isoforms of three DASGs in *sr30* mutant under *P. infestans* infection.

The qRT-PCR assay was performed to measure the relative expression level of different transcript isoforms of three DASGs in *sr30* mutant leaves at 2 days post-inoculated by *P. infestans* using isoform-specific primers (n=6). The relative expression levels of transcript isoforms under different treatments are normalized to the transcript level of *UBC34.2*, *CCR4E.2*, and *ARP8.2* in the WT, respectively. The gene *UBI* was used as an internal control. This experiment was repeated twice with similar results.

**Table S1****.** Information of the SR family proteins identified in *Solanum lycopersicum.*

|  | Subfamily | Gene ID | Name | Previous name | Protein size | Molecular weight |
| --- | --- | --- | --- | --- | --- | --- |
| Animal/Plant subfamily | SR | Solyc01g099810.3.1 | SR30 | SR33 | 265 aa | 30.15 kDa |
|  |  | Solyc03g082380.4.1 | SR32 | SR32 | 281 aa | 31.60 kDa |
|  |  | Solyc06g009060.4.1^a)^ | SR41 | SR41 | 361 aa | 40.89 kDa |
|  |  | Solyc09g075090.1.1 | SR34 | SR35 | 306 aa | 34.34 kDa |
|  | SC | Solyc01g105140.3.1 | SC30b | SC30b | 255 aa | 30.02 kDa |
|  |  | Solyc04g074040.3.1 | SC30a | SC30a | 259 aa | 30.12 kDa |
|  | RSZ | Solyc08g069120.4.1^a)^ | RSZ28 | RSZ21b | 244 aa | 27.5 kDa |
| Plant specific subfamily | SCL | Solyc01g005820.4.1 | SCL29 | SCL29 | 253 aa | 29.04 kDa |
|  |  | Solyc01g080660.3.1 | SCL19 | SCL31 | 167 aa | 19.39 kDa |
|  | RS2Z | Solyc05g054920.5.1 | RS2Z35 | RS2Z35 | 307 aa | 34.74 kDa |
|  |  | Solyc09g005980.4.1 | RS2Z36 | RS2Z36 | 316 aa | 35.94 kDa |
|  | RS | Solyc01g096180.4.1 | RS29 | RS29 | 246 aa | 29.13 kDa |
|  |  | Solyc01g091750.3.1 | RS30 | RS30 | 251 aa | 29.63 kDa |
|  |  | Solyc03g026240.4.1 | RS42 | RS42 | 371 aa | 42.29 kDa |
|  |  | Solyc10g009330.3.1 | RS25 | RS28 | 212 aa | 24.90 kDa |
|  |  | Solyc11g072340.2.1 | RS41 | RS41 | 355 aa | 40.96 kDa |
|  | SR-like | Solyc10g005590.5.1 | SR46 | SR46 | 417 aa | 46.27 kDa |
|  |  | Solyc06g076670.3.1 | SR46a | SR46a | 387 aa | 46.15 kDa |

**Note:** The information of these SR family proteins is derived from the *S. lycopersicum* genome SL4.0. The previous names of these SR proteins were taken from the result of R. R. E. Rosenkranz et al ^[6]^.

^a)^ (the coding sequence is corrected by PCR (Supplementary File 2)).

**Table S2.** Information of RNA-seq data.

| Sample | Total reads | Mapped reads | Percentage mapped |
| --- | --- | --- | --- |
| WT#1 | 83630372 | 82505179 | 98.65% |
| WT#2 | 81650382 | 80585885 | 98.70% |
| WT#3 | 85489502 | 84407383 | 98.73% |
| *SR30*-OE#1 | 108270954 | 106431666 | 98.30% |
| *SR30*-OE#2 | 82157578 | 80752557 | 98.29% |
| *SR30*-OE#3 | 100928728 | 99269919 | 98.36% |

**Table S3.** Protein sequences of different transcript isoforms of three DASGs.

| Isoform name | Protein sequence |
| --- | --- |
| SlUBC34.1 | MAEKACVKRLQKEYRALCKEPVSHVVARPSPNDILEWHYVLEGSEGTPFAGGLYYGKIKFPPEYPFKPPGISMVTPNGRFMTHKKICLSMSDFHPESWNPMWSVSSILTGLLSFMMDTSPTTGSVTTTVAEKQKLAKTSLAFNCKNPTFRKLFPEYVEKYEEQQLLVHPDQEQVSSMPTQAEISSPLLDGLNSVEPHKDMENQRRKSLPTWLLLLLVSIFGVVMALPLLQL* |
| SlUBC34.2 | MAEKACVKRLQKEYRALCKEPVSHVVARPSPNDILEWHYVLEGSEGTPFAGGLYYGKIKFPPEYPFKPPGISMVTPNGRFMTHKKICLSMSDFHPESWNPMWSVSSILTGLLSFMMDTSPTTGSVTTTVAEKQKLAKTSLAFNCKK* |
| SlCCR4E.1 | MGRTGDGRDQPAEQPSRFSAMKSRIRQHDIGKHRSKKNRGKEKRHKGKAKRAIADDKRKWVYSTHDVSPNQDRVILMSYNILGVKNAAAHEDLYRNVSPKYLDWDYRKKLICKEIRDYNPDIMCFQEVDRFDDLDYLLQKEGFKGVYQARTGDASDGCAIFWNNKLFDILHEESIEFQNFNLRNNVCQLCVFKMNVKSSTKDVSASNSESVSSPSFLVGNIHVLYNPNRGDIKLGQVRLFLESAQRLSHEWGDIPVVLAGDLNSMPQSAMYQFLTSNKLDIQMHDRKQISGQIYPLQNRSFNPRLSYRWSNEELLLATGTGASQLIHQLQLRSAYAGAPGSSRTRENSGEPLATSYHSKFLGTVDYIWHTTEFVPVRVLDTLPVDILRRTGGLPSEKWGSDHLSLVCELAFADEGSET* |
| SlCCR4E.2 | MGRTGDGRDQPAEQPSRFSAMKSRIRQHDIGKHRSKKNRGKEKRHKGKAKRAIADDKRKWVYSTHDVSPNQDRVILMSYNILGVKNAAAHEDLYRNVSPKYLDWDYRKKLICKEIRDYNPDIMCFQEVDRFDDLDYLLQKEGFKGVYQARTGDASDGCAIFWNNKLFDILHEESIEFQNFNLRNNVCQLCVFKMNVKSSTKDVSASNSE* |
| SlARP8.1 | MAMLLRKVWESVSTRSTSSNSTSTSISTVNSMDRFDQMMMYIQTASTGEFDRIPLDIFIQILKILGPKESAKLTSVCKSWKYIVSDNRLWIYFLQNHHEPWDSTFFSETHLRSGPLRTFPNSVPELSFMSIYGQRAQVPGAIIIDGGSGYCKFGWSKYSAPSGRSATFLEFGNIESPMYSRLRHFFSTIYTRMQVKTSTQPIIVSIPICHYDDTESDKAARTQLKDAIHSALFDMNVPAVCAVNQAVLALFAARKVSGIVVNIGFNQTSIVPILYGKVMHQVGVEVVGIGALKLTGFLKEQMQQKNIYFGSLYTVRTLKENLCYIALDYEAELSKDTNASFQIGSEGCFTLSEERFKTGEILFQPRIAGVRAMGLQNAVALCMEHCHDAELMVDDSWYKTVVLAGGSACLPGLAERLEKEVCELLPPCMSNGIRVLPPPYGVDSAWYGAKLIGNLSTFPSSWCVMKKQFRHRSRRKFMW* |
| SlARP8.2 | MAMLLRKVWESVSTRSTSSNSTSTSISTVNSMDRFDQMMMYIQTASTGEFDRIPLDIFIQILKILGPKESAKLTSVCKSWKYIVSDNRLWIYFLQNHHEPWDSTFFSETHLRSGPLRTFPNSVPELSFMSIYGQRAQVPGAIIIDGGSGYCKFGWSKYSAPSGRSATFLEFGNIESPMYSRLRHFFSTIYTRMQVKTSTQPIIVSIPICHYDDTESDKAARTQLKDAIHSALFDMNVPAVCAVNQAVLALFAARKVSGIVVNIGFNQTSIVPILYGKVMHQVGVEVVGIGALKLTGFLKEQMQQKNIYFGSLYTVRTLKENLCYIALDYEAELSKDTNASFQIGSEGCFTLSEERFKTGEILFQPRIAGVYVHITLFGLLVRLLNNSSFNYFP* |

**Note:** The asterisks represent the stop codon.

**Table S4.** Primer sequences used in this study.

| **Primer name** | **5'-3' sequence** |
| --- | --- |
| pBinGFP2-SlSR30-F | TACAAGGGTACCCCCATGGGTCGTCTAAGTCGG |
| pBinGFP2-SlSR30-R | GGATCCGTCGACCCCCTATATAGCAATTCCCAG |
| pBinGFP2-SlSCL29-F | TACAAGGGTACCCCCATGAGGAGGAGAAGTTAC |
| pBinGFP2-SlSCL29-R | GGATCCGTCGACCCCTCATGGTGAGTAGGGAGC |
| pBinGFP2-SlSR41-F | TACAAGGGTACCCCCATGAGTCGTTCAAGTAGGAC |
| pBinGFP2-SlSR41-R | GGATCCGTCGACCCCTCACCTCGACAAACTCCC |
| pBinGFP2-SlSC30a-F | TACAAGGGTACCCCCATGTCTCACTTCGGTAGA |
| pBinGFP2-SlSC30a-R | GGATCCGTCGACCCCTCAATCATCAGCATCAGA |
| pBinGFP2-SlRS41-F | TACAAGGGTACCCCCATGAGGCCAATTTTCTGTG |
| pBinGFP2-SlRS41-R | GGATCCGTCGACCCCCTAGGAGCGGGATCTTTC |
| pBinGFP2-SlRS2Z35-F | TACAAGGGTACCCCCATGCCGCGGTATGATGAC |
| pBinGFP2-SlRS2Z35-R | GGATCCGTCGACCCCTTAGGGTGACTCACTGCC |
| pBinGFP2-SlSR30^ΔIDR2^-F2 | TGGGCGAGGATGCCTCATGGCAAGAC |
| pBinGFP2-SlSR30^ΔIDR2^-R1 | AAGTCTTGCCATGAGGCATCCTCGCCCACCATGTGC |
| pBinGFP2-SlSR30^ΔIDR5^-F2 | CGCAATCAAGCTTTTTCCCCTGCGCTC |
| pBinGFP2-SlSR30^ΔIDR5^-R1 | GGGGAAAAAGCTTGATTGCGAAACAGAGAGTCATC |
| PBinGFP2-F | AAGACCCCAACGAGAAGC |
| PBinGFP2-R | GAACCCTAATTCCCTTATCTG |
| qRT-SlSR30-F | ACCACCTGGTTATGCGTTCGT |
| qRT-SlSR30-R | TCGCCCACCATGTGCAAGTT |
| qRT-SlActin-F | TGTGGGAGATGAAGCTCAATCG |
| qRT-SlActin-R | TCAAACTATCAGTGAGGTCACG |
| qRT-SlLRR22-F | AAGATTGGAGGTTGCCATTGGAGC |
| qRT-SlLRR22-R | ATCGCGATGAATGATCGGTGGAGT |
| qRT-SlWRKY28-F | ACAGATGCAGCTACCTCATCCTCA |
| qRT-SlWRKY28-R | GTGCTCAAAGCCTCATGGTTCTTG |
| qRT-SlGRAS2-F | AATCCAAGGGATGAGCTTCT |
| qRT-SlGRAS2-R | CCACCAACGTGACCACCTT |
| TRV2-SlSR30-F | CGACAAGACCCTGCAAATTCTCTCGAGCATATA |
| TRV2-SlSR30-R | GAGAAGAGCCCTGCACTATATAGCAATTCCCAG |
| TRV2-F | AGTCTATCATTCTGTTACTA |
| TRV2-R | CGTAGGTTTAAATTGAACCT |
| qRT-SlSR30-F2 | ACGGGCATCGCTTGCGAGTT |
| qRT-SlSR30-R2 | TGCCATGAGGCAGAAGACGGT |
| GFP-F | ATGGTGAGCAAGGGCGA |
| GFP-R | TTGTACAGCTCGTCCATGCC |
| qRT-SlUBI-F | TCGTAAGGAGTGCCCTAATGCTGA |
| qRT-SlUBI-R | CAATCGCCTCCAGCCTTGTTGTAA |
| qRT-SlUBC34.1-F | TGCTTTCAATTGTAAGAACCCTACC |
| qRT-SlUBC34.1-R | CAGCAACAACCAAGTTGGCA |
| qRT-SlUBC34.2-F | TGCTTTCAATTGTAAGAAGTGAGTCA |
| qRT-SlUBC34.2-R | GATGCAAAATTAGTGAGTTAAAAAGGT |
| qRT-SlCCR4E.1-F/  Nb-SlCCR4E.1-qRT-F | TCGGAGAGTGTATCCTCGCC |
| qRT-SlCCR4E.1-R/  Nb-SlCCR4E.1-qRT-R | TCAAAAACTGATACATTGCACTCTGA |
| qRT-SlCCR4E.2-F/Nb-SlCCR4E.2-qRT-F | TCAAATTCGGAGTAAGTCTGCTCT |
| qRT-SlCCR4E.2-R/  Nb-SlCCR4E.2-qRT-R | CACGATTGGGGTTGTAGAGTACA |
| qRT-SlARP8.1-F | CAGGAGTGCGTGCTATGGG |
| qRT-SlARP8.1-R | TGGGGGAGGAAGTACTCTGA |
| qRT-SlARP8.2-F | CGCATTGCAGGAGTGTACGT |
| qRT-SlARP8.2-R/  Nb-SlARP8.2-qRT-R | CGGCATCATCAGTTGACCATAG |
| pBinGFP-SlUBC34.1-F | TACAAGGGTACCCCCATGGCAGAAAAGGCATGTGTAA |
| pBinGFP-SlUBC34.1-R | AGGATCCGTCGACCCCTCAAAGCTGAAGTAGCGGCAG |
| pBinGFP-SlUBC34.2-R1 | CGTAGTTGACTCACCATAAAAGAGAGCAGTCCA |
| pBinGFP-SlUBC34.2-F2 | TCTTTTATGGTGAGTCAACTACGAGTTCAACGC |
| pBinGFP-SlUBC34.2-R2 | AGAGGATCCGTCGACCCCTTATCTGAAACAAATACAAGGTATGCA |
| pBinGFP-SlCCR4E.1-F | TACAAGGGTACCCCCATGGGCCGAACCGGCGAC |
| pBinGFP-SlCCR4E.1-R | AGGATCCGTCGACCCCTCAAGTCTCACTCCCTTCATCA |
| pBinGFP-SlCCR4E.2-R | AGAGGATCCGTCGACCCCTTACTCCGAATTTGAAGCACTCAC |
| pBinGFP-SlARP8.1-F | TACAAGGGTACCCCCATGGCTATGTTGTTGCGCAA |
| pBinGFP-SlARP8.1-R | GGATCCGTCGACCCCTCACCACATAAACTTGCGTCTCG |
| pBinGFP-SlARP8.2-R | AGAGGATCCGTCGACCCCCTAAGGGAAGTAATTGAAACTGGAGTT |
| 86900-proSlSR30-F1 | CAAGAATTCAAGCTTGGAGTGACGTTTCAAATATAG |
| 86900-proSlSR30-R1 | CTTGCTCACCATATTGCCTGAAATCATTACCCAAA |
| 86900-proSlSR30-F2 | CAGGCAATATGGTGAGCAAGGGCGAGG |
| 86900-proSlSR30-R2 | TGTGCATCCTCTAGTAGCGTCTATATAGCAATTCCCAGATCGCC |
| His-MBP-GFP-F | CTGTATTTTCAGGGCGAATTCGAAAACCTCTACTTCCAATCGAGCATGGTGAGCAAGGG |
| His-MBP-GFP-R | CAGGTCGACTCTAGAGAATTCTCATCTAGAGGATCCGTCGACC |
| His-MBP-GFP-SlSR30-F | CTGTATTTTCAGGGCGAATTCGAAAACCTCTACTTCCAATCGAGCATGGTGAGCAAGGG |
| His-MBP-GFP-SlSR30-R | CAGGTCGACTCTAGAGAATTCCTATATAGCAATTCCCAG |
| pMAL-F | ACAATAACAACAACCTCG |
| pMAL-R | GTAAAACGACGGCCAGT |
| pBinGFP2-SlSR30^ΔIDR2&5^-FUS-R1 | CGTTTGAGGCCATTATAGCAATTCCCAGATCGCCG |
| pBinGFP2-SlSR30^ΔIDR2&5^-FUS-F2 | TGCTATAATGGCCTCAAACGATTATACCC |
| pBinGFP2-SlSR30^ΔIDR2&5^-FUS-R2 | GGATCCGTCGACCCCCTAGCTGTTGTACTGGTTCTGC |
| 35S::SlUBC34gDNA-F | TTACAATTATCGATACAATGGCAGAAAAGGCATGTGT |
| 35S::SlUBC34gDNA-R | CTCATTAAAGCAGGACAAGCTCAAAGCTGAAGTAGCGGCAG |
| 35S::SlCCR4EgDNA-F1 | TTACAATTATCGATACAATGGGCCGAACCGGCGA |
| 35S::SlCCR4EgDNA-R1 | GTTATACCTGTCCCAATTTGATGTCTCCAC |
| 35S::SlCCR4EgDNA-F2 | CAAATTGGGACAGGTATAACTTTTAAGCAACAACTAAT |
| 35S::SlCCR4EgDNA-R2 | CTCATTAAAGCAGGACAAGCTCAAGTCTCACTCCCTTCATCA |
| 35S::SlARP8gDNA-F1 | TTACAATTATCGATACAATGGCTATGTTGTTGCGCA |
| 35S::SlARP8gDNA-R1 | CTTACTTGGAACAATAGATGTCTGGTTAAACC |
| 35S::SlARP8gDNA-F2 | CTATTGTTCCAAGTAAGTTTTTTATTCATGTACTGGAC |
| 35S::SlARP8gDNA-R2 | CTCATTAAAGCAGGACAAGCTCACCACATAAACTTGCGTCTC |
| Nb-SlUBC34.1-qRT-F | ATTGTAAGAACCCTACCTTTAG |
| Nb-SlUBC34.1-qRT-R | TTGGTTCTCCATGTCCTT |
| Nb-SlUBC34.2-qRT-F | CGAGTTCAACGCTGCATACC |
| Nb-SlUBC34.2-qRT-R | AGGTAGGGCTGAGATGCAAA |
| Nb-SlARP8.1-qRT-F | GACTCTGAAAGAGAACCTTTGTTATA |
| Nb-SlARP8.1-qRT-R | CACGCACTCCTGCAATGC |
| Nb-SlARP8.2-qRT-F | GCAGGAGTGTACGTTCATAT |
| qRT-NbActin-F | ACCATCAATGATCGGAATGGAA |
| qRT-NbActin-R | GCTCATCCTATCAGCAATGCC |
| SlSR30-nLUC-F | AGGACGACGACGACAAATCCATGGGTCGTCTAAGTCGGAC |
| SlSR30-nLUC-R | CCAGATGAGCCACCGCCCCGTATAGCAATTCCCAGATCGCCG |
| SR30^ΔIDR2&5^-FUS-nLUC-R | CCAGATGAGCCACCGCCCCGGCTGTTGTACTGGTTCTGCT |
| cLUC-SlSR30-F | TACGCGTCCCGGGGCGGTACCATGGGTCGTCTAAGTCGG |
| cLUC-SlSR30-R | ATTTGTTGGATGCCGGGTACCCTATATAGCAATTCCCAG |
| cLUC-SlRS29-F | TACGCGTCCCGGGGCGGTACCATGAGGCCTTTATTCGTG |
| cLUC-SlRS29-R | ATTTGTTGGATCCCGGGTACCTTACCTCCGAACAGGAAAC |
| cLUC-SlSCL29-F | TACGCGTCCCGGGGCGGTACCATGAGGAGGAGAAGTTAC |
| cLUC-SlSCL29-R | ATTTGTTGGATCCCGGGTACCTCATGGTGAGTAGGGAGC |
| cLUC-SlSCL19-F | TACGCGTCCCGGGGCGGTACCATGGGAAGGTACAGAAGC |
| cLUC-SlSCL19-R | ATTTGTTGGATCCCGGGTACCTTAGCATCTTGAGTCACG |
| cLUC-SlSR46-F | TACGCGTCCCGGGGCGGTACCATGGCGAAACCAGGTCGAGG |
| cLUC-SlSR46-R | ATTTGTTGGATCCCGGGTACCCTAGGGCTTACGAGGTGGTG |
| 1300-cLUC-F | AGTCAAGTAACAACCGCGA |
| 1300-cLUC-R | CGATGATACGAACGAAAGCTC |
| 86988-nLUC-F | GGACACGCTCGAGTATAAGAGCTC |
| 86988-nLUC-R | TCTATCCGCTGGAAGATGGA |
| BD-SR30-F | GGAGGCCGAATTCCCGATGGGTCGTCTAAGTCGG |
| BD-SR30-R | GGAGGCCGAATTCCCGATGGGTCGTCTAAGTCGG |
| BD- SR30^ΔIDR2&5^-FUS-R | GGTCGACGGATCCCCCTAGCTGTTGTACTGGTTCT |
| T7 | TAATACGACTCACTATAGGG |
| 3′BD | TAAGAGTCACTTTAAAATTTGTAT |
| SlSR30-KO-F | AATGTTTGAACAAGCTGCTTA |
| SlSR30-KO-R | CCTAATAGAATGATTCCCAAGGA |

**Note:** The primer sequences of qRT-LRR22-F/R, qRT-WRKY28-F/R, and qRT-GARS2-F/R are obtained from the Kim et al. study ^[7]^.

**Table S5** The CDS sequence of the LCD of the FUS used in this study.

| **Gene name** | **5'-3' sequence** |
| --- | --- |
| The LCD of FUS | ATGGCCTCAAACGATTATACCCAACAAGCAACCCAAAGCTATGGGGCCTACCCCACCCAGCCCGGGCAGGGCTATTCCCAGCAGAGCAGTCAGCCCTACGGACAGCAGAGTTACAGTGGTTATAGCCAGTCCACGGACACTTCAGGCTATGGCCAGAGCAGCTATTCTTCTTATGGCCAGAGCCAGAACACAGGCTATGGAACTCAGTCAACTCCCCAGGGATATGGCTCGACTGGCGGCTATGGCAGTAGCCAGAGCTCCCAATCGTCTTACGGGCAGCAGTCCTCCTACCCTGGCTATGGCCAGCAGCCAGCTCCCAGCAGCACCTCGGGAAGTTACGGTAGCAGTTCTCAGAGCAGCAGCTATGGGCAGCCCCAGAGTGGGAGCTACAGCCAGCAGCCTAGCTATGGTGGACAGCAGCAAAGCTATGGACAGCAGCAAAGCTATAATCCCCCTCAGGGCTATGGACAGCAGAACCAGTACAACAGC |

**Supplementary References**

[1] J. Huang, X. Lu, H. Wu, Y. Xie, Q. Peng, L. Gu, J. Wu, Y. Wang, A. S. N. Reddy, S. Dong, *Phytophthora* Effectors Modulate Genome-wide Alternative Splicing of Host mRNAs to Reprogram Plant Immunity, *Mol Plant* **2020**, *13* (10), 1470.

[2] G. S. Ali, S. G. Palusa, M. Golovkin, J. Prasad, J. L. Manley, A. S. Reddy, Regulation of plant developmental processes by a novel splicing factor, *PLoS One* **2007**, *2* (5), e471.

[3] G. S. Ali, M. Golovkin, A. S. Reddy, Nuclear localization and in vivo dynamics of a plant-specific serine/arginine-rich protein, *Plant J* **2003**, *36* (6), 883.

[4] I. S. Day, M. Golovkin, S. G. Palusa, A. Link, G. S. Ali, J. Thomas, D. N. Richardson, A. S. Reddy, Interactions of SR45, an SR-like protein, with spliceosomal proteins and an intronic sequence: insights into regulated splicing, *Plant J* **2012**, *71* (6), 936.

[5] G. S. Ali, A. S. Reddy, ATP, phosphorylation and transcription regulate the mobility of plant splicing factors, *J Cell Sci* **2006**, *119* (Pt 17), 3527.

[6] R. R. E. Rosenkranz, S. Bachiri, S. Vraggalas, M. Keller, S. Simm, E. Schleiff, S. Fragkostefanakis, Identification and Regulation of Tomato Serine/Arginine-Rich Proteins Under High Temperatures, *Front Plant Sci* **2021**, *12*, 645689.

[7] J. G. Kim, X. Li, J. A. Roden, K. W. Taylor, C. D. Aakre, B. Su, S. Lalonde, A. Kirik, Y. Chen, G. Baranage, H. McLane, G. B. Martin, M. B. Mudgett, *Xanthomonas* T3S Effector XopN suppresses PAMP-triggered immunity and interacts with a tomato atypical receptor-like kinase and TFT1, *Plant Cell* **2009**, *21* (4), 1305.

**The legends for supplementary files**

**Dataset S1.** Sequence information of the SR family proteins in tomato.

**Dataset S2.** The lists of DASGs and DEGs in the comparison of *SR30*-OE versus WT.

**File S1.** The predicted domain of the SR proteins in tomato.

**File S2.** Gene sequences of two SR protein genes corrected.

**File S3.** IDR sequences of SR30.

**Movie S1****.** FRAP of nuclear condensates formed by GFP-SR30.

**Movie S2.** Fusion of two nuclear condensates formed by GFP-SR30.

File S1:

**SR subfamily** RRM ψRRM SR-rich

**>Solyc01g099810.3.1**

MSYSNMGRLSRTIYVGNLPGDIREREVEDLFYKYGPIVEIDLKVPPRPPGYAFVEFEDPRDADDAIRGRDGYDFDGHRLRVELAHGGRGSSSYDRHSSYSSASRSGLSRRSDYRVLVSGLPSSASWQDLKDHMRRAGDVCFSQVFRDRDGMRGIVDYTNYDDMRYAIKKLDDSLFRNQFSRAYIRVDKYDKRHSYSRSPSPYNSRSRSYSRSRSPRRSYSSQSGSVSPRGKYSRRSVSISPSRAFSPALSLSRSGFRGDLGIAI*

**>Solyc03g082380.4.1**

MSGRFSRSIYVGNLPADIKELEVEDLFYKYGRILDIELKIPPRPPCYCFVEFESSRDAEDAIRGRDGYNFDGCRLRVELAHGGRGPSSSSDRRGSYGSGGGGGGGRHGISRHSDYRVIIRGLPSSASWQDLKDHMRKAGDVCFAEVSRDSEGTFGLVDYTNYEDMKYAIRKLDDTEFRNPWTRTYIRVREYKGSPSRSRSRSRSRSRSRSRSRRSPARSISRSPPPKSRSASPVKSTRSRSLSRSMSRSRSRSRSRSRSKSRSPSRSRSASPQQARSNSG*

**>Solyc06g009060.4.1 correction**

MSRSSRTIYVGNLPGDIREREVEDLFYKYGPIAHIDLKIPPRPPGYAFVEFEEARDAEDAIRGRDGYEFDGHRLRVELAHGGRGNSSSDRYNSGNNSGHNGGRNNHKFGAPKRTEYRVLVTGLPHSASWQDLKDHMRRAGDVCFSQVFREGSGTTGIVDYTNYDDMKYAIKKLDESEFRNAFSRSTIRVKEHDSRSRSRSRSYSRGKSGSRSRSRSYSRSRSRSKSPKAKSSKRTRSRSRSVSSQPRSGLKGRSLSRSPSRSRSPVPSRPKRVSKSPKPRDSRRSQSLSKSPKPRDSRRSESPSKSPKLRDSRRSKSMSKSPEPRNSRRSPSRSKSPKPRNSRRSPSRSRSRSRSGSLSR*

**>Solyc09g075090.1.1**

MRNMLFASLYPARLHFLKTLIFKISSSSTLSALDLPSRSSRTLYVGNLPGDVREREVEDLFYKYGPIAHIELKIPPRPPGYAFVEFEEARDAEDAIRGRDGYDFDGHRLRVELAHGGRGNSSSNDRYGGGGGGRGQRGGGVSRRSDYRVLVTGLPHSASWQDLKDHMRRAGDVCFSQVFRDGSGTTGIIDYTNYDDMKYAIKKLDDSEFRNAFSRATIRVKEYDRSRSRSRSRSRSYSRGKSVSRSRSRSRSRSRSKSKSKSPKVKSSKRSRSRSRSVSSQSRSGSKGRPVSRFWICAAVCGFAY*

**SC subfamily** RRM SR-rich

>**Solyc01g105140.3.1**

MSHFGRTGPPDIADTYSLLVLNITFRTSADDLFPLFDKYGKVVDIFIPRDRRTGESRGFAFVRYKYAEEAQKAVDRLDGRVVDGREMAVQFAKYGPNAERIHQGRIIEKVPGFKGSSRSRSPRRRYRDDYHRDREYRRSRSRSVDRYERDRYRQRERDYRHRSRSRSLSPDYDRDRGRRRDRKHYRRSPSVDSASPSRRSPSPHRKESPPRSLSPTKGSPVRRVRNERSPTPRSRSPPGRAMDSRSPSPRVDED*

>**Solyc04g074040.3.1**

MSHFGRSGPPDIKDTYSLLVLNVTFRTTADDLFPLFDKYGKVVDVFIPRDRRTGDSRGFAFVRYKYQDEAQKAVEKLDGRVVDGREIMVRFAKYGPNAERIDKGRILEPVQRPKGRSRSRSPRPRHRDHRDKDSRRRSRSRSRSRSRGRYERDQYRGRDRDNRHRSRSRSPDYHRGRGRGKYDEDRRSRSRSHGRSASPARRSPSPRRSPSPRRTTPPRDASPDGRNHKDRSPTPKSISPRGRRAGSRSPLPRSDADD*

**RSZ subfamily** RRM ZF-CCHC SR-rich

**> Solyc08g069120.4.1 correction**

MHVEPHRRALSVERWVCLGRAIGRLGPHFLFQQNTKSSSFFFTALLFSARVSGISRCVLKMSRVYVGNLDPRVSERELEDEFRIFGVIRSVWVARRPPGYAFIDFDDRRDAQDAIKELDGKNGWRVELSHNSRGGGGGGRGGGRGRSGGSDLKCYECGESGHFARECRTRGGPGAGRRRSRSPPRYRRSPSYGRRSYSPRGRSPRRRSPSPRGRSYSRSPYRGREEAPYVNGNGLRECHRSRS*

**SCL subfamily** N-terminal extension RRM SR-rich

**>****Solyc01g005820.4.1**

MRRRSYSPSPPRGYGRRGRSPSPRGRYAGHGRDGPTSLLVRNLRHDCRPEDLRRPFGQFGPVKDIYLPKDYYTGEPRGFGFVQFVDPADAADAKYQMDGQGFQGRQLTVVFAEENRKKPTEMRSRERSGSHRSSRSYDRRRTPPSRYARPGSHSRDYSPKRRPYSRSVSPEEKRYSRERSYSRSPPRDLSPPPHNGSRSRSQTPVREHPPYNGSPRSRSRSPVRRERSPVRGHSRSPSRSRSRSPGCAPYSP*

**>Solyc01g080660.3.1**

MGRYRSRSRSLSRSYSPVRRKRHDEPRDRRRERRSPGPSGLLVRNIPLSARPEDLRVPFERYGPIRDVYLPKNYHTGEPRGFGFVKFRYAEDAAEAKAHLNNTVIGGRDIRIVFAEDNRKTPREMRKVLSTSGPSARGSYWRHSSPSRRYHSYSRSASPARRDSRC*

**RS2Z subfamily** RRM ZF-CCHC SR-rich

**>Solyc05g054920.5.1**

MPRYDDRYGGTRLYVGHLSSRTRSRDLEDVFSRYGRVRDVDMKRDYAFVEFSDPRDADDARYGLNGRDVDGSRVIVEFAKGVPRGPGGSREFGGRGPPPGTGRCFNCGIDGHWARDCKAGDWKNKCYRCGDRGHIERNCQNSPKKLKRDRSYSRSPSPRRGRSRSRSRSYSRGRSYSRSRSPVKRDRSIEREEKRSRSPRHHRSSPPPSKGRKHSLSPDERSPVERGTPSPRDDRATNGSDRSRSPKDDVRMDERGDISPVEENGRSRSNSPIHREDRSPVEDGSPTGDYENHGSPRGSPRGSESP*

**>Solyc09g005980.4.1**

MPRYDDRVGNSTRLYVGHLSSRTRSRDLERAFSKYGRVRDVDMKHDYAFVEFSDPRDADDARYYLDGRDIDGRRIIVEFAKGVPRGPGGSREYLGKGPAPGSGRCFNCGLEGHWARDCKAGDWKNKCYRCGERGHIERKCPNSPKKLSRRSYSRSPARSKSRSRSRSRSPRRSYSRSRSYSQSRSPPPKREQVDQVKRSRSYSRSPEPRKDSPSPPPKTRKRSPTPEEGSPMEAKSPSSPMREEGAYSQSPRERSVSPSSTRRDSPAPRKYDDDSPAEANGGSRSPSPKYQRNHEDDEDEGEFRNQRSGRESQSP*

**RS subfamily** RRM SR-rich

**>Solyc01g096180.4.1**

MRPLFVGNIEYDIRQPELERLFSKYGRIERLDMKSGLHILAGFAFVYFEDERDAADAIRCLDNMPFGYDKRRLSVEWAKGDRVQPRDDSKVSANQRPTRSLFVINFDPIRTRVRDIERHFEPYGKILNVRIRRNFAFVQFENLEDASKALECTHMSEILDRVVSVEYALRDDGERGDRYDSPRRDYIRHGDSPYRRSPSPMYRRGRPSPDYGRPGIPAYDKYNGSSYDRYRSPEYGSYRRFPVRR*

**>Solyc01g091750.3.1**

MRAIFCGNLEFDARQSDVERLFRRYGKVDRVDMKSGFAFIYMEDERDADDAIRRLDRIEFGKKGRRLRVEWTKDRGSRRPEISRKPAANTRPSKTLFVINFDPVHTQTRDIEKYFEPYGRISNVRIRKNFAFVQYESVDDASRALEATNMSKFMDRVISVEFAIRDDDDRRNGRSPDRRGRDMSPDRRGYDRRRSPSPYRRDRGSPDYGRGAPLNSRPQTRRSPEYGRAESPVNERYHSRSPPPRERSRS*

**>Solyc03g026240.4.1**

MRPIFCGNFEFETRQPELERLFKRYGKVDRVDMKSGFAFVYMDDERDAKDAIQGLDRIEFGRKGRRLRVEWSKEERSRKPEGSKKSSSSFRVSKTLFVINFDPYNTRTRDLERHFDPYGKILNIRIRRNFGFIQFETQEDATRALDATNMSKLMDRVITVEYAIRDDDDRKNGYGPGKTYNQSPRRGYDRGRSRSPRGRDRLSPDYGRGRDRPSPDYGRGRDRPSPDYGRGRDRPISDFDRGRDRPNSDFGRGRDQLSPDYGRGPSRSPKHREGNSEYGRGHSPAVGKERNPGHGNVRSPSPRRERTGPGNGLMSSPLNISPGYGDGPSPSAQRERRDKYSPDGHNRGSSPGPKPEPVGSPVRDGRGSSE*

**>Solyc10g009330.3.1**

MPTTTNKGYAFVYFEDDRDAADAIRGTDNMPFGYERRRLSVEWAKGERGRHHDGGPKSGGNQRPTKTLFVINFDPIRTRVRDIEKHFEPHGKVLHVRIRRNFAFVQFENQEEATRALECTHMSKVLDRVVSVEYALKDDDERGDKYNSPRRDYGRQRDSPYRRSPSPVYRRNRPSPDYGRPRSPVHNGPSYDRYRSPQYGRYRSRSPVRRS*

**>Solyc11g072340.2.1**

MRPIFCGNVEYNARQSELERLFRRYGRVDRVDMKSGFAFVYMDDERDAEDAIRGLDRIEFGRKGRRLRIEWSKEERNGRRPETSRKSSSSVKPSKTLFVINFDPYSTRSRDIERHFDPYGKILNIRIRRNFAFVQYETQEDATRALDATNMSKLMDQVITVEYANKDDDDRRNGFSPDRNRDRGLKRGYDRGRSRSPYGRERGSPDYGRGRARSPSPIRQGRSSPDYGRRPSPNPNHRERDSEYGSGRSPNMRKERNPDHGNGHSPNPRRLRAGSENGEVHSPPEEGLLESGPSPPRVGRRGKYSPDDYRGRSRSPSPRSKPEEIGSPRYGAAESPLPERHRSLSPPTRERSRS*

**SR-like subfamily** RRM SR-rich

**>Solyc10g005590.5.1**

MAKPGRGRAASPSGSSSRSRSRSRSRSRSYTPSNSRSSSSRSPSRSRSRSRSISSSSSASRSASSRSPSRRPPSQRKSPAGVSKRGRSPPPPPESKKASPPPRKVSPIPESRVIHVDQLSRNVNENHLKEIFGNFGEILHVQLAIDHVVNLPKGFAYVEFKTRIDAEKAQLHMDGAQIDGKVVHARFTLPERKKAASPPRAVATSSRRDAPRTDNAPVDVEKDGPKRQQELSPRRKPASSPRRSPIGRRGSPRRGPDSPVRRRANSPFRRGSPPPHRRRPASPMRRRSPSPPLRRHRTPPRGSPRRIRGSPVRRRSPLPPRRRSPRRARSPPRRSPIGRRSRSPIRRPLRSRSRSISPRRGRGAAARRGRSSSYSSSPSPRKGPRKISKSRSPKRRPLRGRSPSNSNSSSSPPRKP*

**>Solyc06g076670.3.1**

MSYSRRSRYSRSPSYDRYSKSVSRSRCVSRSRSRSCDSSDVENPGNNLYVTGLSTRVKERDIEKHFSAEGKVEDVRLVLDPWTHESRGFGFVTMSSVEEADRCIKSLNRSILEGRVITVEKARRRRGRTPTPGKYLGLRTVRVRRESRTYPHYSRNHSPCYSSESYRSRSRSYSPYYRQEHRSYSYYRGRQRSHSSYYSRHHCYSESPYSPYYSRGRSYSRSLSPYNGRDRSYSPDDCYYRRSRYHDYSPDNHRRDRSYSPDDRYYRRSRYRDYSPESHDLSDSPDVRDNRMSRYRDYSPNNSYYYRRNRYRSISRSISPRYRRSYSRSVSPRWSKRSYSRSVSRSSCSRSSYSPNQKKSSKKSRSVSASSRFVSRSVTPRSSPSS*

File S2:

There is a 1 bp deletion in Solyc08g069120.4.1 of the current tomato SL4.0 genome compared to gene application results, the position was indicated with red.

>Solyc08g069120.4.1

ATGCATGTTGAACCCCACAGGCGAGCCCTGAGCGTTGAGCGTTGGGTGTGCTTAGGGCGTGCAATCGGACGCTTAGGGCCCCACTTTTTGTTTCAACAGAACACCAAATCGAGCTCTTTCTTCTTCACTGCACTGTTGTTTTCCGCTAGGGTTTCAGGTATTAGTAGGTGTGTATTGAAAATGTCAAGAGTCTATGTTGGAAATCTGGACCCTAGGGTCAGTGAAAGAGAGCTTGAAGATGAATTCCGCATCTTTGGAGTTATAAGAAGTGTTTGGGTTGCAAGACGCCCCCCTGGCTACGCTTTTATTGACTTCGATGATCGGCGGGATGCACAAGATGCAATCAAAGAGCTGGATGGTAAGAATGGATGGAGAGTGGAGCTTTCACATAATTCTAGAGGTGGAGGTGGTGGGGGCCGTGGAGGAGGTCGAGGTCGATCTGGAGGCTCTGATTTGAAGTGCTACGAATGTGGTGAATCAGGTCATTTTGCTCGTGAGTGCAGAACGCGTGGGGGTCCAGGAGCTGGAAGACGTAGAAGTCGGAGCCCTCCTAGATACCGCAGGAGCCCAAGTTATGGTCGTAGGAGTTACAGTCCACGTGGGCGTTCCCCTAGGCGCCGAAGCCCGTCACCACGTGGTCGCAGCTATAGCCGTTCTCCATATCGAGGTCGAGAAGAAGCTCCATATGTTAATGGAAATGGACTTAGAGAGTGTCACAGAAGCCGAAGCTGA

There are 4 bp insertions in Solyc06g009060.4.1 of the current tomato SL4.0 genome when compared to gene application results, the position was indicated with red.

>Solyc06g009060.4.1

ATGAGTCGTTCAAGTAGGACGATTTATGTTGGTAATCTTCCTGGTGATATTCGTGAGCGAGAAGTGGAGGATCTGTTCTACAAGTATGGCCCGATAGCACATATTGATCTGAAAATTCCACCAAGACCCCCAGGTTATGCTTTTGTTGAGTTTGAAGAGGCACGCGATGCTGAGGATGCTATTCGTGGGCGCGATGGCTATGAATTTGATGGGCATCGTTTGAGGGTTGAGCTTGCACATGGTGGGCGTGGTAACTCATCGTCAGATCGTTATAATAGTGGCAATAATAGTGGCCATAATGGTGGTCGTAATAATCACAAATTTGGAGCGCCCAAACGTACCGAGTATCGAGGCACTTTTAGTTACCGGATTGCCCCATTCAGCATCCTGGCAGGATCTCAAGGATCATATGCGTCGAGCTGGGGATGTTTGTTTCTCACAAGTTTTCCGTGAGGGGAGTGGGACCACTGGGATTGTGGATTATACCAACTATGACGACATGAAATATGCTATAAAAAAACTTGATGAATCTGAGTTTCGGAATGCTTTTTCTCGTTCAACAATTCGGGTGAAGGAACATGATTCTAGAAGCCGCAGCCGCAGCCGTTCTTACTCGAGAGGAAAGAGTGGTAGCCGTAGCCGCAGTCGAAGTTACAGTCGCAGCCGGAGCAGAAGCAAATCTCCTAAAGCTAAGTCGTCAAAGCGTACAAGATCTCGTTCTAGATCTGTCTCTTCTCAGCCCCGTTCTGGGTTAAAAGGACGCTCTTTGTCAAGATCTCCATCAAGATCCAGATCCCCAGTACCATCTCGCCCAAAACGTGTGAGCAAAAGCCCAAAACCTCGCGACTCCAGGAGAAGCCAGAGCTTGAGTAAAAGCCCAAAACCTCGTGATTCCAGGAGAAGCGAGAGTCCAAGCAAGAGCCCCAAACTGCGTGATTCCAGGAGAAGCAAGAGCATGAGTAAAAGCCCCGAACCACGCAATTCCAGGAGGAGCCCCAGCAGGAGCAAAAGCCCCAAACCACGCAATTCCAGGAGAAGCCCTAGCAGGAGCCGCAGCCGGAGTCGCAGTGGGAGTTTGTCGAGGTGA

File S3:

**Information on SR30 IDRs in detail**


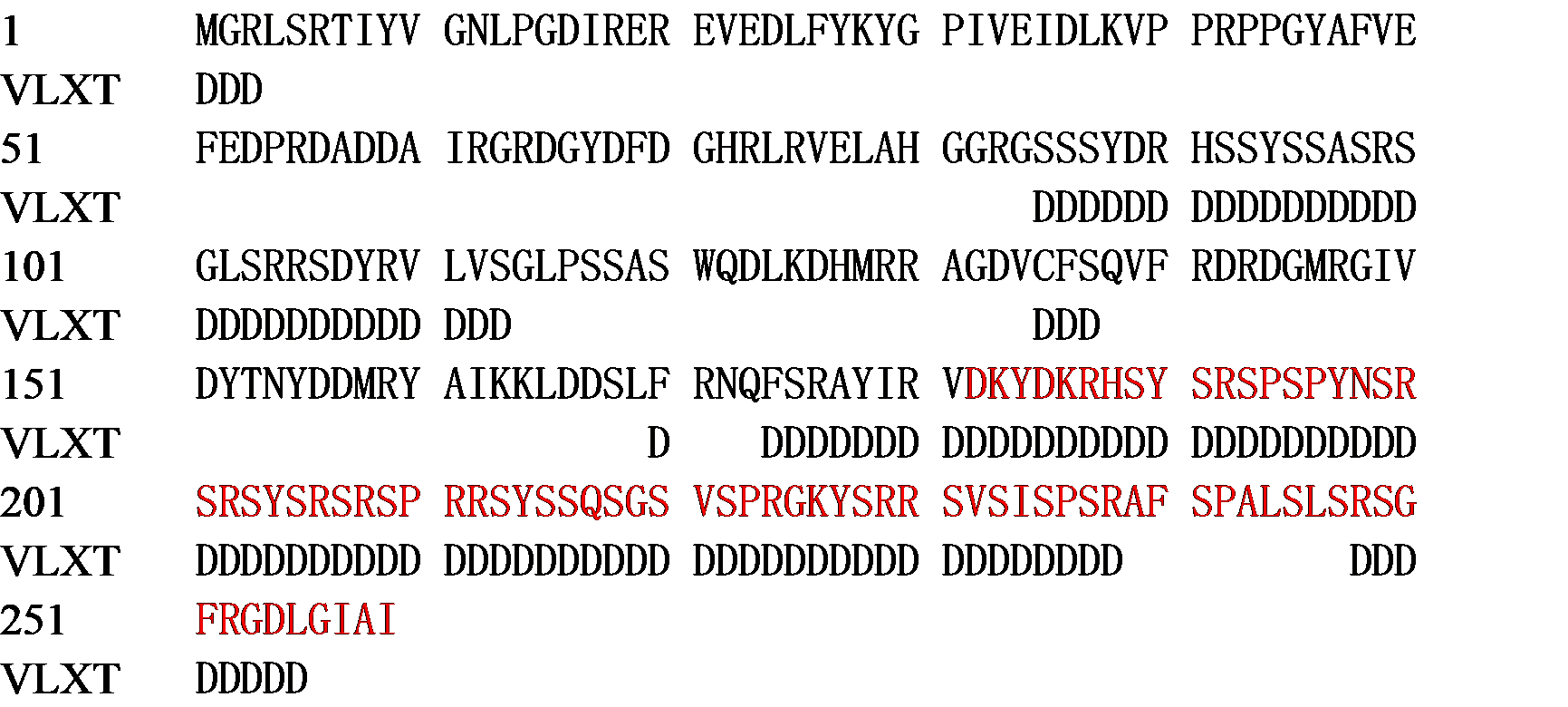


**Note:** D, disorder. Red protein sequence represents the putative region of tomato SR30 that is predicted to undergo phase separation by Emenecker et al (Emenecker et al., 2020).

**References**

**Emenecker, R.J., Holehouse, A.S., and Strader, L.C.** (2020). Emerging Roles for Phase Separation in Plants. Developmental Cell **55**:69-83. 10.1016/j.devcel.2020.09.010.
