# Supplementary material for: A Phase‐Separated SR Protein Reprograms Host Pre‐mRNA Splicing to Enhance Disease Susceptibility
Source: Adv Sci (Weinh). 2025 May 8;12(27):2500072. doi: 10.1002/advs.202500072 (PMC12279203; doi:10.1002/advs.202500072)
Supplement: Supplementary file 6 — Supplemental File S1 [file ADVS-12-2500072-s008.docx]

**SR subfamily** RRM ψRRM SR-rich

**>Solyc01g099810.3.1**

MSYSNMGRLSRTIYVGNLPGDIREREVEDLFYKYGPIVEIDLKVPPRPPGYAFVEFEDPRDADDAIRGRDGYDFDGHRLRVELAHGGRGSSSYDRHSSYSSASRSGLSRRSDYRVLVSGLPSSASWQDLKDHMRRAGDVCFSQVFRDRDGMRGIVDYTNYDDMRYAIKKLDDSLFRNQFSRAYIRVDKYDKRHSYSRSPSPYNSRSRSYSRSRSPRRSYSSQSGSVSPRGKYSRRSVSISPSRAFSPALSLSRSGFRGDLGIAI*

**>Solyc03g082380.4.1**

MSGRFSRSIYVGNLPADIKELEVEDLFYKYGRILDIELKIPPRPPCYCFVEFESSRDAEDAIRGRDGYNFDGCRLRVELAHGGRGPSSSSDRRGSYGSGGGGGGGRHGISRHSDYRVIIRGLPSSASWQDLKDHMRKAGDVCFAEVSRDSEGTFGLVDYTNYEDMKYAIRKLDDTEFRNPWTRTYIRVREYKGSPSRSRSRSRSRSRSRSRSRRSPARSISRSPPPKSRSASPVKSTRSRSLSRSMSRSRSRSRSRSRSKSRSPSRSRSASPQQARSNSG*

**>Solyc06g009060.4.1 correction**

MSRSSRTIYVGNLPGDIREREVEDLFYKYGPIAHIDLKIPPRPPGYAFVEFEEARDAEDAIRGRDGYEFDGHRLRVELAHGGRGNSSSDRYNSGNNSGHNGGRNNHKFGAPKRTEYRVLVTGLPHSASWQDLKDHMRRAGDVCFSQVFREGSGTTGIVDYTNYDDMKYAIKKLDESEFRNAFSRSTIRVKEHDSRSRSRSRSYSRGKSGSRSRSRSYSRSRSRSKSPKAKSSKRTRSRSRSVSSQPRSGLKGRSLSRSPSRSRSPVPSRPKRVSKSPKPRDSRRSQSLSKSPKPRDSRRSESPSKSPKLRDSRRSKSMSKSPEPRNSRRSPSRSKSPKPRNSRRSPSRSRSRSRSGSLSR*

**>Solyc09g075090.1.1**

MRNMLFASLYPARLHFLKTLIFKISSSSTLSALDLPSRSSRTLYVGNLPGDVREREVEDLFYKYGPIAHIELKIPPRPPGYAFVEFEEARDAEDAIRGRDGYDFDGHRLRVELAHGGRGNSSSNDRYGGGGGGRGQRGGGVSRRSDYRVLVTGLPHSASWQDLKDHMRRAGDVCFSQVFRDGSGTTGIIDYTNYDDMKYAIKKLDDSEFRNAFSRATIRVKEYDRSRSRSRSRSRSYSRGKSVSRSRSRSRSRSRSKSKSKSPKVKSSKRSRSRSRSVSSQSRSGSKGRPVSRFWICAAVCGFAY*

**SC subfamily** RRM SR-rich

>**Solyc01g105140.3.1**

MSHFGRTGPPDIADTYSLLVLNITFRTSADDLFPLFDKYGKVVDIFIPRDRRTGESRGFAFVRYKYAEEAQKAVDRLDGRVVDGREMAVQFAKYGPNAERIHQGRIIEKVPGFKGSSRSRSPRRRYRDDYHRDREYRRSRSRSVDRYERDRYRQRERDYRHRSRSRSLSPDYDRDRGRRRDRKHYRRSPSVDSASPSRRSPSPHRKESPPRSLSPTKGSPVRRVRNERSPTPRSRSPPGRAMDSRSPSPRVDED*

>**Solyc04g074040.3.1**

MSHFGRSGPPDIKDTYSLLVLNVTFRTTADDLFPLFDKYGKVVDVFIPRDRRTGDSRGFAFVRYKYQDEAQKAVEKLDGRVVDGREIMVRFAKYGPNAERIDKGRILEPVQRPKGRSRSRSPRPRHRDHRDKDSRRRSRSRSRSRSRGRYERDQYRGRDRDNRHRSRSRSPDYHRGRGRGKYDEDRRSRSRSHGRSASPARRSPSPRRSPSPRRTTPPRDASPDGRNHKDRSPTPKSISPRGRRAGSRSPLPRSDADD*

**RSZ subfamily** RRM ZF-CCHC SR-rich

**> Solyc08g069120.4.1 correction**

MHVEPHRRALSVERWVCLGRAIGRLGPHFLFQQNTKSSSFFFTALLFSARVSGISRCVLKMSRVYVGNLDPRVSERELEDEFRIFGVIRSVWVARRPPGYAFIDFDDRRDAQDAIKELDGKNGWRVELSHNSRGGGGGGRGGGRGRSGGSDLKCYECGESGHFARECRTRGGPGAGRRRSRSPPRYRRSPSYGRRSYSPRGRSPRRRSPSPRGRSYSRSPYRGREEAPYVNGNGLRECHRSRS*

**SCL subfamily** N-terminal extension RRM SR-rich

**>****Solyc01g005820.4.1**

MRRRSYSPSPPRGYGRRGRSPSPRGRYAGHGRDGPTSLLVRNLRHDCRPEDLRRPFGQFGPVKDIYLPKDYYTGEPRGFGFVQFVDPADAADAKYQMDGQGFQGRQLTVVFAEENRKKPTEMRSRERSGSHRSSRSYDRRRTPPSRYARPGSHSRDYSPKRRPYSRSVSPEEKRYSRERSYSRSPPRDLSPPPHNGSRSRSQTPVREHPPYNGSPRSRSRSPVRRERSPVRGHSRSPSRSRSRSPGCAPYSP*

**>Solyc01g080660.3.1**

MGRYRSRSRSLSRSYSPVRRKRHDEPRDRRRERRSPGPSGLLVRNIPLSARPEDLRVPFERYGPIRDVYLPKNYHTGEPRGFGFVKFRYAEDAAEAKAHLNNTVIGGRDIRIVFAEDNRKTPREMRKVLSTSGPSARGSYWRHSSPSRRYHSYSRSASPARRDSRC*

**RS2Z subfamily** RRM ZF-CCHC SR-rich

**>Solyc05g054920.5.1**

MPRYDDRYGGTRLYVGHLSSRTRSRDLEDVFSRYGRVRDVDMKRDYAFVEFSDPRDADDARYGLNGRDVDGSRVIVEFAKGVPRGPGGSREFGGRGPPPGTGRCFNCGIDGHWARDCKAGDWKNKCYRCGDRGHIERNCQNSPKKLKRDRSYSRSPSPRRGRSRSRSRSYSRGRSYSRSRSPVKRDRSIEREEKRSRSPRHHRSSPPPSKGRKHSLSPDERSPVERGTPSPRDDRATNGSDRSRSPKDDVRMDERGDISPVEENGRSRSNSPIHREDRSPVEDGSPTGDYENHGSPRGSPRGSESP*

**>Solyc09g005980.4.1**

MPRYDDRVGNSTRLYVGHLSSRTRSRDLERAFSKYGRVRDVDMKHDYAFVEFSDPRDADDARYYLDGRDIDGRRIIVEFAKGVPRGPGGSREYLGKGPAPGSGRCFNCGLEGHWARDCKAGDWKNKCYRCGERGHIERKCPNSPKKLSRRSYSRSPARSKSRSRSRSRSPRRSYSRSRSYSQSRSPPPKREQVDQVKRSRSYSRSPEPRKDSPSPPPKTRKRSPTPEEGSPMEAKSPSSPMREEGAYSQSPRERSVSPSSTRRDSPAPRKYDDDSPAEANGGSRSPSPKYQRNHEDDEDEGEFRNQRSGRESQSP*

**RS subfamily** RRM SR-rich

**>Solyc01g096180.4.1**

MRPLFVGNIEYDIRQPELERLFSKYGRIERLDMKSGLHILAGFAFVYFEDERDAADAIRCLDNMPFGYDKRRLSVEWAKGDRVQPRDDSKVSANQRPTRSLFVINFDPIRTRVRDIERHFEPYGKILNVRIRRNFAFVQFENLEDASKALECTHMSEILDRVVSVEYALRDDGERGDRYDSPRRDYIRHGDSPYRRSPSPMYRRGRPSPDYGRPGIPAYDKYNGSSYDRYRSPEYGSYRRFPVRR*

**>Solyc01g091750.3.1**

MRAIFCGNLEFDARQSDVERLFRRYGKVDRVDMKSGFAFIYMEDERDADDAIRRLDRIEFGKKGRRLRVEWTKDRGSRRPEISRKPAANTRPSKTLFVINFDPVHTQTRDIEKYFEPYGRISNVRIRKNFAFVQYESVDDASRALEATNMSKFMDRVISVEFAIRDDDDRRNGRSPDRRGRDMSPDRRGYDRRRSPSPYRRDRGSPDYGRGAPLNSRPQTRRSPEYGRAESPVNERYHSRSPPPRERSRS*

**>Solyc03g026240.4.1**

MRPIFCGNFEFETRQPELERLFKRYGKVDRVDMKSGFAFVYMDDERDAKDAIQGLDRIEFGRKGRRLRVEWSKEERSRKPEGSKKSSSSFRVSKTLFVINFDPYNTRTRDLERHFDPYGKILNIRIRRNFGFIQFETQEDATRALDATNMSKLMDRVITVEYAIRDDDDRKNGYGPGKTYNQSPRRGYDRGRSRSPRGRDRLSPDYGRGRDRPSPDYGRGRDRPSPDYGRGRDRPISDFDRGRDRPNSDFGRGRDQLSPDYGRGPSRSPKHREGNSEYGRGHSPAVGKERNPGHGNVRSPSPRRERTGPGNGLMSSPLNISPGYGDGPSPSAQRERRDKYSPDGHNRGSSPGPKPEPVGSPVRDGRGSSE*

**>Solyc10g009330.3.1**

MPTTTNKGYAFVYFEDDRDAADAIRGTDNMPFGYERRRLSVEWAKGERGRHHDGGPKSGGNQRPTKTLFVINFDPIRTRVRDIEKHFEPHGKVLHVRIRRNFAFVQFENQEEATRALECTHMSKVLDRVVSVEYALKDDDERGDKYNSPRRDYGRQRDSPYRRSPSPVYRRNRPSPDYGRPRSPVHNGPSYDRYRSPQYGRYRSRSPVRRS*

**>Solyc11g072340.2.1**

MRPIFCGNVEYNARQSELERLFRRYGRVDRVDMKSGFAFVYMDDERDAEDAIRGLDRIEFGRKGRRLRIEWSKEERNGRRPETSRKSSSSVKPSKTLFVINFDPYSTRSRDIERHFDPYGKILNIRIRRNFAFVQYETQEDATRALDATNMSKLMDQVITVEYANKDDDDRRNGFSPDRNRDRGLKRGYDRGRSRSPYGRERGSPDYGRGRARSPSPIRQGRSSPDYGRRPSPNPNHRERDSEYGSGRSPNMRKERNPDHGNGHSPNPRRLRAGSENGEVHSPPEEGLLESGPSPPRVGRRGKYSPDDYRGRSRSPSPRSKPEEIGSPRYGAAESPLPERHRSLSPPTRERSRS*

**SR-like subfamily** RRM SR-rich

**>Solyc10g005590.5.1**

MAKPGRGRAASPSGSSSRSRSRSRSRSRSYTPSNSRSSSSRSPSRSRSRSRSISSSSSASRSASSRSPSRRPPSQRKSPAGVSKRGRSPPPPPESKKASPPPRKVSPIPESRVIHVDQLSRNVNENHLKEIFGNFGEILHVQLAIDHVVNLPKGFAYVEFKTRIDAEKAQLHMDGAQIDGKVVHARFTLPERKKAASPPRAVATSSRRDAPRTDNAPVDVEKDGPKRQQELSPRRKPASSPRRSPIGRRGSPRRGPDSPVRRRANSPFRRGSPPPHRRRPASPMRRRSPSPPLRRHRTPPRGSPRRIRGSPVRRRSPLPPRRRSPRRARSPPRRSPIGRRSRSPIRRPLRSRSRSISPRRGRGAAARRGRSSSYSSSPSPRKGPRKISKSRSPKRRPLRGRSPSNSNSSSSPPRKP*

**>Solyc06g076670.3.1**

MSYSRRSRYSRSPSYDRYSKSVSRSRCVSRSRSRSCDSSDVENPGNNLYVTGLSTRVKERDIEKHFSAEGKVEDVRLVLDPWTHESRGFGFVTMSSVEEADRCIKSLNRSILEGRVITVEKARRRRGRTPTPGKYLGLRTVRVRRESRTYPHYSRNHSPCYSSESYRSRSRSYSPYYRQEHRSYSYYRGRQRSHSSYYSRHHCYSESPYSPYYSRGRSYSRSLSPYNGRDRSYSPDDCYYRRSRYHDYSPDNHRRDRSYSPDDRYYRRSRYRDYSPESHDLSDSPDVRDNRMSRYRDYSPNNSYYYRRNRYRSISRSISPRYRRSYSRSVSPRWSKRSYSRSVSRSSCSRSSYSPNQKKSSKKSRSVSASSRFVSRSVTPRSSPSS*
