# Supplementary material for: A Phase‐Separated SR Protein Reprograms Host Pre‐mRNA Splicing to Enhance Disease Susceptibility
Source: Adv Sci (Weinh). 2025 May 8;12(27):2500072. doi: 10.1002/advs.202500072 (PMC12279203; doi:10.1002/advs.202500072)
Supplement: Supplementary file 7 — Supplemental File S2 [file ADVS-12-2500072-s002.docx]

There is a 1 bp deletion in Solyc08g069120.4.1 of the current tomato SL4.0 genome compared to gene application results, the position was indicated with red.

>Solyc08g069120.4.1

ATGCATGTTGAACCCCACAGGCGAGCCCTGAGCGTTGAGCGTTGGGTGTGCTTAGGGCGTGCAATCGGACGCTTAGGGCCCCACTTTTTGTTTCAACAGAACACCAAATCGAGCTCTTTCTTCTTCACTGCACTGTTGTTTTCCGCTAGGGTTTCAGGTATTAGTAGGTGTGTATTGAAAATGTCAAGAGTCTATGTTGGAAATCTGGACCCTAGGGTCAGTGAAAGAGAGCTTGAAGATGAATTCCGCATCTTTGGAGTTATAAGAAGTGTTTGGGTTGCAAGACGCCCCCCTGGCTACGCTTTTATTGACTTCGATGATCGGCGGGATGCACAAGATGCAATCAAAGAGCTGGATGGTAAGAATGGATGGAGAGTGGAGCTTTCACATAATTCTAGAGGTGGAGGTGGTGGGGGCCGTGGAGGAGGTCGAGGTCGATCTGGAGGCTCTGATTTGAAGTGCTACGAATGTGGTGAATCAGGTCATTTTGCTCGTGAGTGCAGAACGCGTGGGGGTCCAGGAGCTGGAAGACGTAGAAGTCGGAGCCCTCCTAGATACCGCAGGAGCCCAAGTTATGGTCGTAGGAGTTACAGTCCACGTGGGCGTTCCCCTAGGCGCCGAAGCCCGTCACCACGTGGTCGCAGCTATAGCCGTTCTCCATATCGAGGTCGAGAAGAAGCTCCATATGTTAATGGAAATGGACTTAGAGAGTGTCACAGAAGCCGAAGCTGA

There are 4 bp insertions in Solyc06g009060.4.1 of the current tomato SL4.0 genome when compared to gene application results, the position was indicated with red.

>Solyc06g009060.4.1

ATGAGTCGTTCAAGTAGGACGATTTATGTTGGTAATCTTCCTGGTGATATTCGTGAGCGAGAAGTGGAGGATCTGTTCTACAAGTATGGCCCGATAGCACATATTGATCTGAAAATTCCACCAAGACCCCCAGGTTATGCTTTTGTTGAGTTTGAAGAGGCACGCGATGCTGAGGATGCTATTCGTGGGCGCGATGGCTATGAATTTGATGGGCATCGTTTGAGGGTTGAGCTTGCACATGGTGGGCGTGGTAACTCATCGTCAGATCGTTATAATAGTGGCAATAATAGTGGCCATAATGGTGGTCGTAATAATCACAAATTTGGAGCGCCCAAACGTACCGAGTATCGAGGCACTTTTAGTTACCGGATTGCCCCATTCAGCATCCTGGCAGGATCTCAAGGATCATATGCGTCGAGCTGGGGATGTTTGTTTCTCACAAGTTTTCCGTGAGGGGAGTGGGACCACTGGGATTGTGGATTATACCAACTATGACGACATGAAATATGCTATAAAAAAACTTGATGAATCTGAGTTTCGGAATGCTTTTTCTCGTTCAACAATTCGGGTGAAGGAACATGATTCTAGAAGCCGCAGCCGCAGCCGTTCTTACTCGAGAGGAAAGAGTGGTAGCCGTAGCCGCAGTCGAAGTTACAGTCGCAGCCGGAGCAGAAGCAAATCTCCTAAAGCTAAGTCGTCAAAGCGTACAAGATCTCGTTCTAGATCTGTCTCTTCTCAGCCCCGTTCTGGGTTAAAAGGACGCTCTTTGTCAAGATCTCCATCAAGATCCAGATCCCCAGTACCATCTCGCCCAAAACGTGTGAGCAAAAGCCCAAAACCTCGCGACTCCAGGAGAAGCCAGAGCTTGAGTAAAAGCCCAAAACCTCGTGATTCCAGGAGAAGCGAGAGTCCAAGCAAGAGCCCCAAACTGCGTGATTCCAGGAGAAGCAAGAGCATGAGTAAAAGCCCCGAACCACGCAATTCCAGGAGGAGCCCCAGCAGGAGCAAAAGCCCCAAACCACGCAATTCCAGGAGAAGCCCTAGCAGGAGCCGCAGCCGGAGTCGCAGTGGGAGTTTGTCGAGGTGA
