# Supplementary material for: A Phase‐Separated SR Protein Reprograms Host Pre‐mRNA Splicing to Enhance Disease Susceptibility
Source: Adv Sci (Weinh). 2025 May 8;12(27):2500072. doi: 10.1002/advs.202500072 (PMC12279203; doi:10.1002/advs.202500072)
Supplement: Supplementary file 8 — Supplemental File S3 [file ADVS-12-2500072-s003.docx]

**Information on SR30 IDRs in detail**


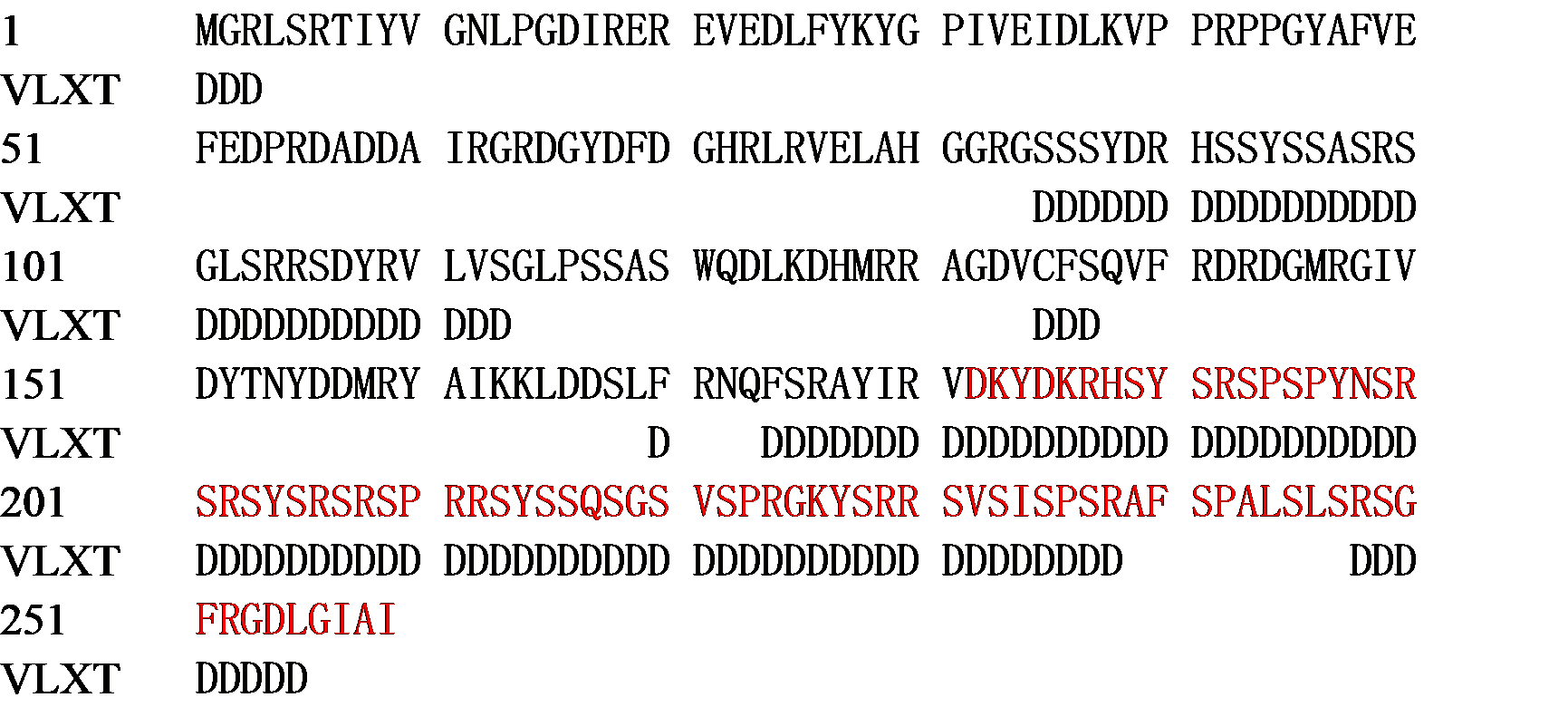


**Note:** D, disorder. Red protein sequence represents the putative region of tomato SR30 that is predicted to undergo phase separation by Emenecker et al (Emenecker et al., 2020).

**References**

**Emenecker, R.J., Holehouse, A.S., and Strader, L.C.** (2020). Emerging Roles for Phase Separation in Plants. Developmental Cell **55**:69-83. 10.1016/j.devcel.2020.09.010.
